# Repurposing CRISPR-Cas12b for mammalian genome engineering

**DOI:** 10.1038/s41421-018-0069-3

**Published:** 2018-11-27

**Authors:** Fei Teng, Tongtong Cui, Guihai Feng, Lu Guo, Kai Xu, Qingqin Gao, Tianda Li, Jing Li, Qi Zhou, Wei Li

**Affiliations:** 1 State Key Laboratory of Stem Cell and Reproductive Biology, Institute of Zoology, Chinese Academy of Sciences, Beijing, 100101 China; 2 Institute for Stem Cell and Regeneration, Chinese Academy of Sciences, Beijing, 100101 China; 3 University of Chinese Academy of Sciences, Beijing, 100049 China

## Abstract

The prokaryotic CRISPR-Cas adaptive immune systems provide valuable resources to develop genome editing tools, such as CRISPR-Cas9 and CRISPR-Cas12a/Cpf1. Recently, CRISPR-Cas12b/C2c1, a distinct type V-B system, has been characterized as a dual-RNA-guided DNA endonuclease system. Though being active in vitro, its cleavage activity at endogenous genome remains to be explored. Furthermore, the optimal cleavage temperature of the reported Cas12b orthologs is higher than 40 °C, which is unsuitable for mammalian applications. Here, we report the identification of a Cas12b system from the *Alicyclobacillus acidiphilus* (AaCas12b), which maintains optimal nuclease activity over a wide temperature range (31 °C–59 °C). AaCas12b can be repurposed to engineer mammalian genomes for versatile applications, including single and multiplex genome editing, gene activation, and generation of gene mutant mouse models. Moreover, whole-genome sequencing reveals high specificity and minimal off-target effects of AaCas12b-meditated genome editing. Our findings establish CRISPR-Cas12b as a versatile tool for mammalian genome engineering.

## Introduction

CRISPR-Cas (clustered regularly interspaced short palindromic repeats and CRISPR-associated protein) systems provide adaptive immunity in archaea and bacteria by employing a combination of Cas effector proteins and CRISPR RNAs (crRNAs)^1–4^. To date, two classes (class 1 and 2) including six types (type I–VI) CRISPR-Cas systems are characterized based on pronounced functional and evolutionary modularity^5^. Among class 2 CRISPR-Cas systems, type II Cas9 systems and type V-A Cas12a/Cpf1 systems have been harnessed for genome editing and hold tremendous promise for biomedical research^6–9^.

Recently, the type V-B CRISPR-Cas12b/C2c1 system has been identified as a dual-RNA-guided DNA endonuclease system with distinct features from Cas9 and Cas12a^10^. First, Cas12b was reported to generate staggered ends distal to the protospacer adjacent motif (PAM) site in vitro when reconstituted with the crRNA/tracrRNA duplex^10,11^. Second, although the RuvC domain of Cas12b is similar to that of Cas9 and Cas12a, its putative Nuc domain shares no sequence or structural similarity to the HNH domain of Cas9 and the Nuc domain of Cas12a^11,12^. Moreover, Cas12b possesses a smaller size than the most widely-used SpCas9 and Cas12a (AacCas12b: 1,129 amino acids (aa), SpCas9: 1,369 aa, AsCas12a: 1,353aa, LbCas12a: 1,274 aa)^9,10,13^, making it suitable for adeno-associated virus (AAV)-mediated in vivo delivery for gene therapy. Compared with the small-sized Cas9, such as the SaCas9 and CjCas9, Cas12b recognizes simpler PAM sequences (AacCas12b: 5′-TTN-3′, SaCas9: 5′-NNGRRT-3′, CjCas9: 5′-NNNNRYAC-3′)^8,10,14^, which can significantly increase the targeting range of the genome. Most importantly, Cas12b has minimal off-target effects^12^ and thus may serve as a safer choice for therapeutic and clinical applications. However, the previously identified Cas12b nuclease (AacCas12b) maintains optimal cleavage activity in vitro at the temperature ranging between 40 °C and 55 °C^10^, which is not suitable for mammalian genome editing. Given the broad applications of Cas9 and Cas12a as genome engineering tools^15–19^, and the diversity of CRISPR-Cas systems^5^, we sought to explore the potential applications of Cas12b systems in mammalian genome engineering.

Here, we report the identification and engineering of Cas12b from *Alicyclobacillus acidiphilus* (AaCas12b), which enables robust genome editing in mammalian cells and mice using a chimeric single-guide RNA (sgRNA). Meanwhile, AaCas12b has features, including relatively small size, increased stability in human plasma, and high specificity, which makes it suitable for therapeutic genome editing.

## Results

### In vitro characterization of AaCas12b

**Fig. 1:**
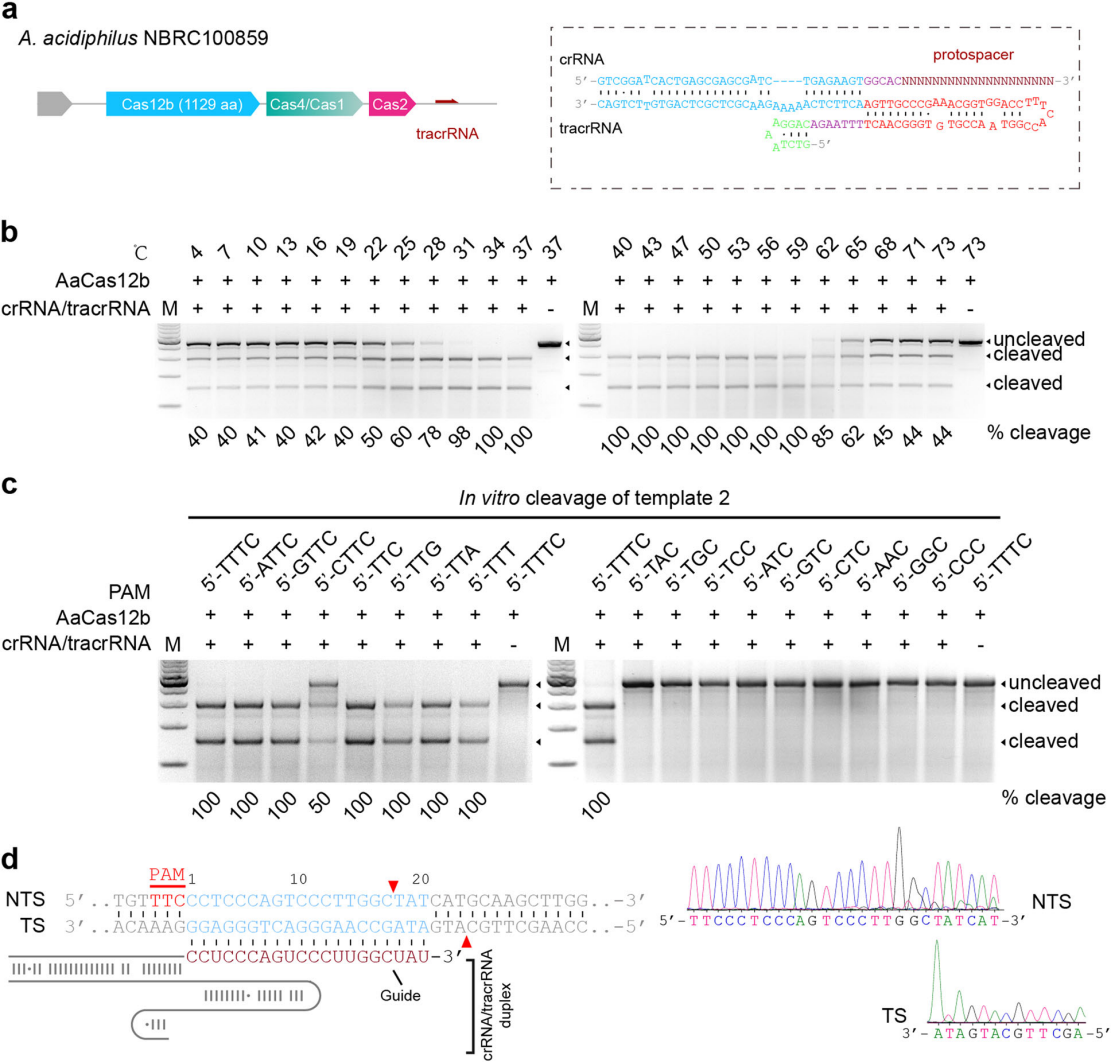
Cas12b nucleases cleave DNA in vitro guided by crRNA/tracrRNA. **a** Schematic illustration of the genomic architecture of CRISPR-Cas12b from *Alicyclobacillus acidiphilus* (NBRC 100859) (left) and the crRNA/tracrRNA duplex (right). **b** In vitro cleavage activity of AaCas12b at various temperatures. The cleavage rate is shown under the cleaved lanes. **c** In vitro validation of the PAM requirements of AaCas12b showing that PAMs matching the 5′-TTN sequence can be efficiently cleaved. The cleavage rate is shown under the cleaved lanes. **d** Cleavage site determination of AaCas12b by sequencing the cleavage products. The cleavage sites are indicated by red triangles in the left panel. TS target strand, NTS non-target strand

To seek Cas12b orthologs capable of mammalian genome editing, we synthesized the human codon-optimized Cas12b sequences, and their cognate crRNA and tracrRNA sequences from *Alicyclobacillus acidiphilus* (NBRC 100859, AaCas12b), *Alicyclobacillus kakegawensis* (NBRC 103104, AkCas12b), *Alicyclobacillus macrosporangiidus* (DSM 17980, AmCas12b), and *Bacillus sp*. (NSP2.1, BsCas12b) (Fig. 1a, Supplementary Fig. S1a and Supplementary Table S1 and Supplementary Sequences). Then we expressed and purified these Cas12b proteins from *E. coli*, and applied them to reconstitute Cas12b ribonucleoproteins (RNPs) with in vitro-transcribed crRNAs and tracrRNAs for in vitro DNA cleavage assay (Supplementary Fig. S1b and Supplementary Table S2 and Supplementary Sequences). Given that Cas12b mediates DNA interference by recognition of the T-rich PAM at the 5′-end of the protospacer sequence^10^, we used PCR-generated double-stranded DNAs (dsDNAs) bearing the 5′-NTTN PAM as templates (Supplementary Table S2). The in vitro DNA cleavage assay results showed that at 37 °C, only AaCas12b and AkCas12b RNPs could cleave the target DNAs, with AaCas12b being more potent than AkCas12b (Supplementary Fig. S1c).

Intriguingly, we found that as a thermoacidophilic bacterium-derived protein, AaCas12b could maintain the nuclease activity over a wide range of temperature and pH values (Fig. 1b and Supplementary Fig. S1d, e). AaCas12b could cleave target DNAs between 4 °C and 100 °C in vitro, and maintained the maximal cleavage activity between 31 °C and 59 °C (Fig. 1b and Supplementary Fig. S1d). For pH value, AaCas12b adapted to a wide range from pH 1.0 to pH 12.0, with highest cleavage activity between pH 1.0 and pH 8.0 (Supplementary Fig. S1e). Since the nuclease activity of Cas12b is metal-dependent, we further determined the metal ion dependence of AaCas12b. The results showed that Mg^2+^ as well as Ca^2+^, Mn^2+^, Sr^2+^, Ni^2+^, Fe^2+^, and Co^2+^, but not Zn^2+^ or Cu^2+^, enabled AaCas12b to cleave target DNAs (Supplementary Fig. S1f, g).

Next, we performed in vitro cleavage assay using DNA templates bearing various PAMs, and identified that AaCas12b utilized the 5′-TTN PAM for target DNA recognition (Fig. 1c and Supplementary Table S2), while AkCas12b recognized the 5′-TTTN PAM (Supplementary Fig. S1h and Supplementary Table S2). We further employed Sanger sequencing of the cleaved dsDNAs to map the cleavage sites of AaCas12b. The results showed that AaCas12b cleaved the complementary DNA strand at 23-base pair (bp) upstream of the PAM sequence, and the non-complementary DNA strand at 17-bp upstream of the PAM sequence, which generated a six-nucleotide (nt) 5′-overhang (Fig. 1d and Supplementary Fig. S1i). This cleavage pattern is distinct from the blunt ends generated by Cas9^13^ but similar to the Cas12a-mediated target cleavage^9^.

### AaCas12b can robustly edit the mammalian genomes in human and mouse cells

**Fig. 2:**
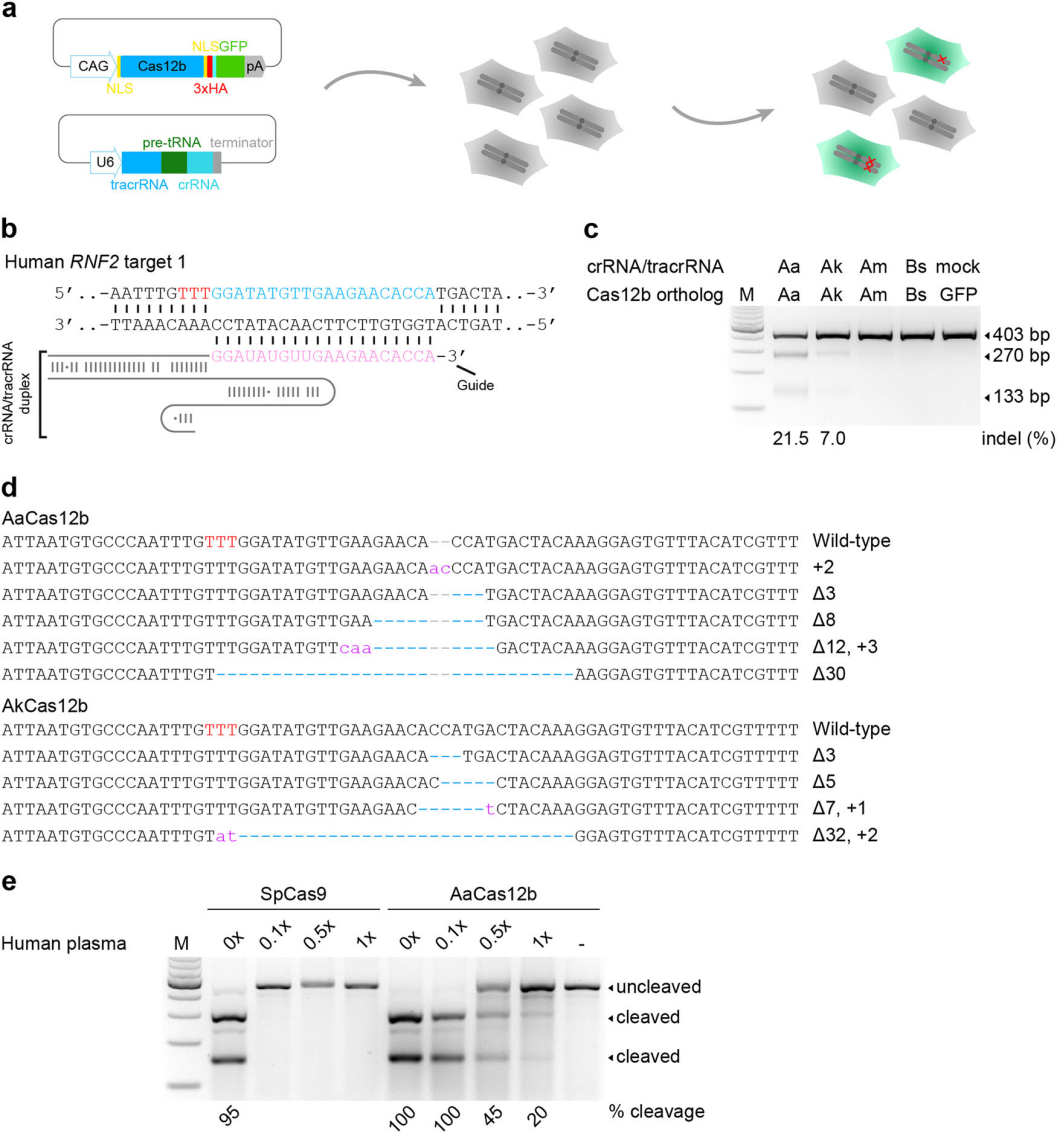
Genome editing by Cas12b nucleases in human 293FT cells. **a** Schematic illustration of the eukaryotic expression strategy of the Cas12b and its cognate guide RNA (gRNA). The pre-tRNA^Gly^, an endogenous RNA processing system for cleavage of transcripts, was hijacked to simultaneously express the tracrRNA and crRNA using a single human U6 promoter. **b** Schematic illustration of the human *RNF2* target site 1 and crRNA/tracrRNA duplex. Red letters indicate the PAM sequences. **c** T7EI analysis of the indels produced by Cas12b orthologs (AaCas12b, AkCas12b, AmCas12b, and BsCas12b) at the human *RNF2* target site 1. The indel rate is shown under the lanes with mutation. mock, an U6 empty vector without crRNA/tracrRNA expression. GFP, an empty backbone vector without Cas12b protein expression. **d** Sanger sequencing results showing the indels in human *RNF2* target site 1 produced by AaCas12b and AkCas12b. Blue dashes, deleted bases; purple lowercases, insertions or mutations; red uppercases, PAM. **e** Effects of human plasma incubation on the nuclease activity of SpCas9 and AaCas12b. After incubation in human plasma at indicated concentrations for 12 h at 37 °C, in vitro DNA cleavage assay was conducted. The cleavage rate is shown under the cleaved lanes

Next, we explored the capacity of the Cas12b systems to cleave endogenous genomic loci in mammalian cells. We cloned the four Cas12b sequences into eukaryotic expression vectors under the CAG promoter, and crRNA/tracrRNA within one polycistron gene hijacked by pre-tRNA^Gly^^20^ under human U6 polymerase III promoter (Fig. 2a and Supplementary Sequences). Two nuclear localization signals (NLSs) were attached to each end of the Cas12b proteins to ensure their nuclear compartmentalization in mammalian cells (Supplementary Fig. S2a). Then the vectors were transfected into human 293FT cells and mouse embryonic stem cells (ESCs) to target two sites of the human *RNF2* gene and one site of the mouse *Nrl* gene, respectively (Fig. 2b, Supplementary Fig. S2b, c and Supplementary Table S3). T7 endonuclease I (T7EI) assay showed that both AaCas12b and AkCas12b induced targeted mutations at site 1 of *RNF2* gene (Fig. 2c), but only AaCas12b induced targeted mutations at site 2 of *RNF2* gene and in *Nrl* gene (Supplementary Fig. S2b, c), suggesting that AaCas12b was more robust than AkCas12b. Consistently, sequence insertions and deletions (indels) at the target sites were detected by Sanger sequencing (Fig. 2d and Supplementary Fig. S2d, e), demonstrating that the engineered Cas12b systems could generate site-specific mutations in mammalian cells. The thermostable nuclease activity of AaCas12b suggested that it might be more stable in human blood. Indeed, although SpCas9 lost its activity even after incubation with low concentrations of human plasma, similarly as previously reported^21^, AaCas12b maintained a robust nuclease activity to cleave target DNAs even after 12 h-incubation with human plasma at 37 °C (Fig. 2e), suggesting that AaCas12b might be suitable for in vivo RNP delivery to achieve genome editing. Collectively, these results showed that AaCas12b could serve as a genome editing tool to engineer human and mouse cells.

### Multiplex genome engineering in mammalian cells using a chimeric single-guide RNA

**Fig. 3:**
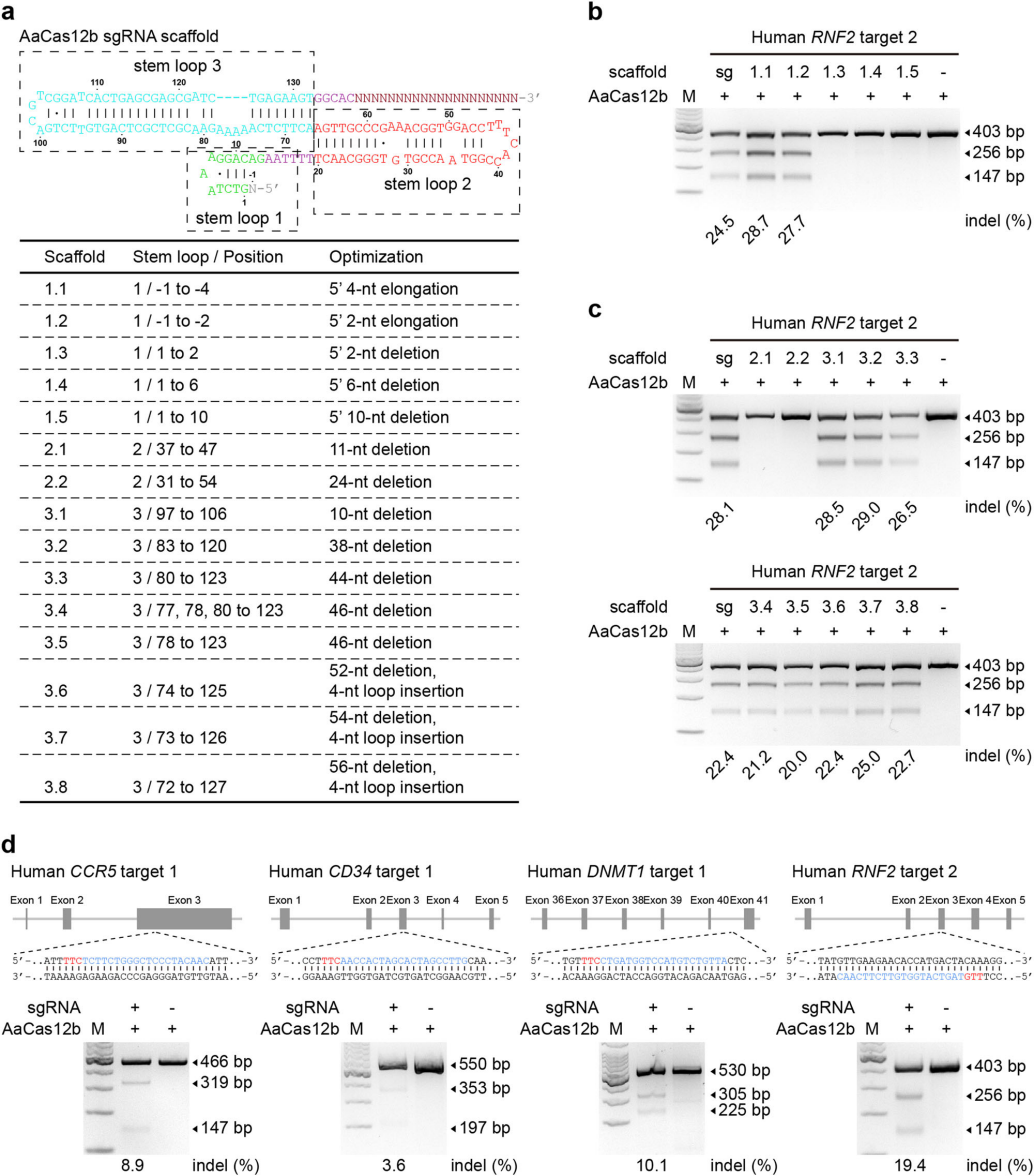
Engineering of the AaCas12b system for multiplex mammalian genome editing. **a** Schematic illustration of the sequences and structure of chimeric single guide (sgRNA) (top), and depiction of the truncation strategy of the sgRNA scaffold (bottom). **b** Effects of truncation of stem-loop 1 within the sgRNA scaffold on the nuclease activity of AaCas12b in human cells. The indel rate is shown under the lanes with mutation. **c** Effects of truncation of stem-loop 2 and stem-loop 3 within the sgRNA scaffold on the nuclease activity of AaCas12b in human cells. The indel rate is shown under the lanes with mutation. **d** AaCas12b facilitates multiplex genome editing by simultaneously targeting *CCR5*, *CD34*, *DNMT1*, and *RNF2*, using four individual sgRNAs containing spacers. (*Top*) Schematic illustration of the target sites in the human genome. (*Bottom*) All the four sgRNAs mediate efficient protospacer cleavage. The indel rate is shown under the lanes with mutation

Next, we characterized the guide RNA requirements for the AaCas12b-mediated genome editing. To simplify the AaCas12b-mediated genome editing, a chimeric single-guide RNA was designed by fusion of crRNA/tracrRNA transcripts (Fig. 3a), which contained three stem-loop structures according to previous reports^10^. T7EI assay showed that the sgRNA could direct AaCas12b to target sites for cleavage in human cells as efficiently as the dual-RNA guide (Supplementary Fig. S4a). To characterize the sequence requirements of the sgRNA, we truncated the sequences of each stem-loop (Fig. 3a and Supplementary Fig. S4b). We found that truncations in stem-loop 1 and stem-loop 2 completely abolished both the in vitro and in vivo endonuclease activity of AaCas12b (Fig. 3b, c and Supplementary Fig. S4c, d), suggesting that they were indispensable. However, truncations in stem-loop 3, which was formed by the crRNA/tracrRNA duplex, did not affect the AaCas12b nuclease activity (Fig. 3c and Supplementary Fig. S4c, d). We further explored the programmability of sgRNA modules by attaching a functional MS2 RNA hairpin to the sgRNA scaffold^22,23^ (Supplementary Fig. S5a-c). We showed that insertion of MS2 sequences into the 5′-end of the sgRNA or the stem-loop 3 had no effect on the target recognition and cleavage activity of the AaCas12b systems in human cells (Supplementary Fig. S5d), indicating that the sgRNA could be repurposed for other functions.

As a type of RNA-guided nucleases, we showed that simultaneous transfection of AaCas12b and paired sgRNAs targeting human *RNF2* gene resulted in large fragment deletions (Supplementary Fig. S6a, b). Moreover, by simultaneously transfecting human 293FT cells with four sgRNAs targeting the human *CCR5*, *CD34*, *DNMT1*, and *RNF2* genes, all the four genes were mutated as detected by the T7EI assay (Fig. 3d). Simultaneous mutations could also be generated in mouse ESCs by the CRISPR-AaCas12b system (Supplementary Fig. S6c). These results demonstrated that AaCas12b could enable robust multiplex genome editing in mammalian cells.

To further compare the frequencies of target mutations generated by AaCas12b, AsCas12a, and SpCas9, we conducted endogenous gene editing experiments at eight genomic sites. Each site contained three PAM sequences that can be recognized by AaCas12b (5′-TTN-3′), SpCas9 (5′-NGG-3′)^13^, and AsCas12a (5′-TTTN-3′)^9^, respectively (Supplementary Fig. S3 and Supplementary Table S3). T7EI assay results showed that each nuclease had a wide range of mutation frequencies at these sites tested, and the overall efficiency of AaCas12b was similar to or higher than AsCas12a, but was lower than SpCas9 (Supplementary Fig. S3). Moreover, AaCas12b recognized 5′-TTN PAM (Fig. 1c), instead of the 5′-TTTN PAM utilized by AsCas12a^9^, indicating that AaCas12b has wider genome coverage and may edit some genomic sites that AsCas12a couldn’t target (Supplementary Fig. S3).

### Gene activation based on deactivated AaCas12b (dAaCas12b)

**Fig. 4:**
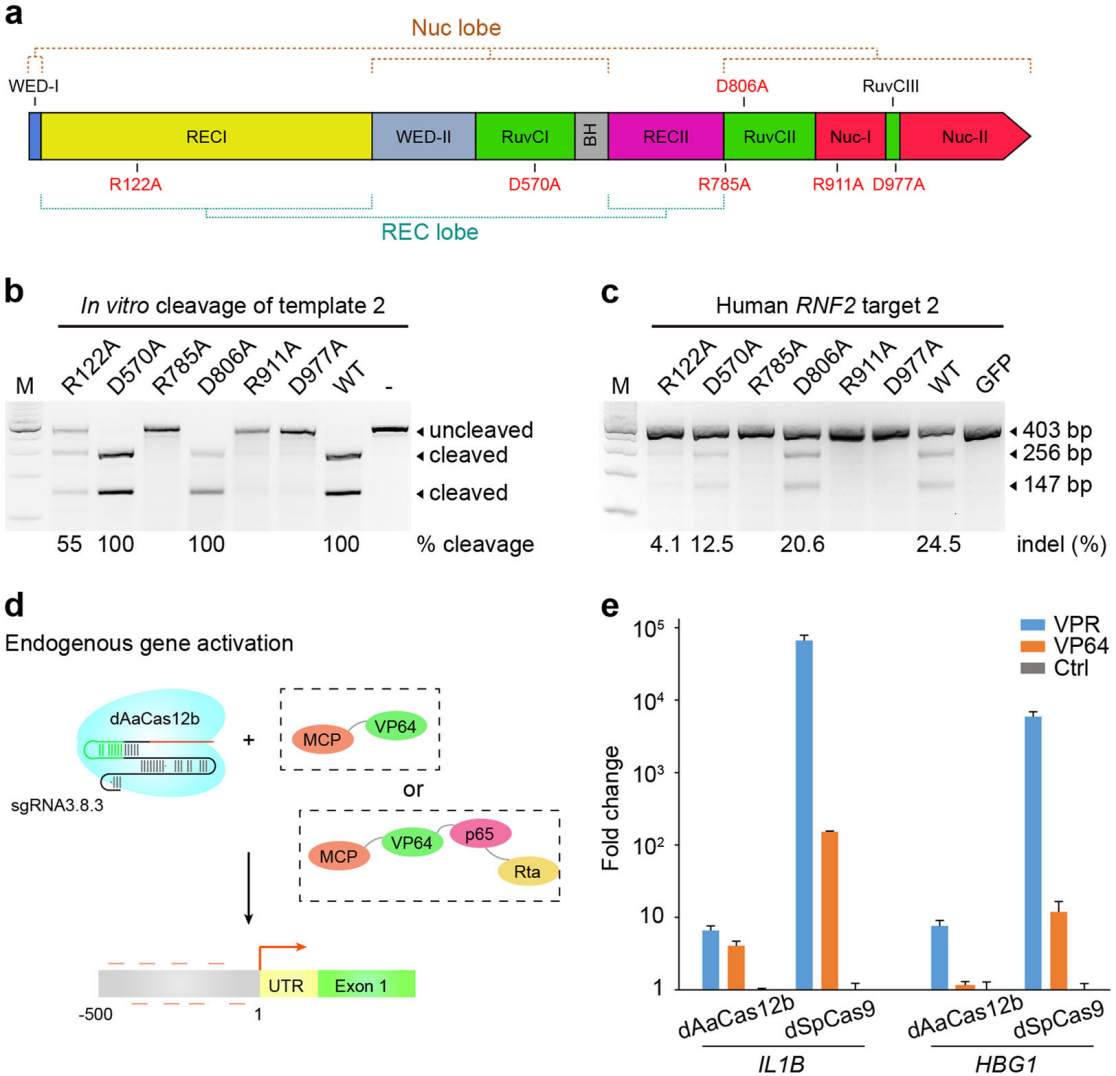
dAaCas12b-medaited gene activation in human cells. **a** Schematic illustration of AaCas12b domain structure showing the positions of catalytic residue mutations. The catalytic residues were identified based on sequence homology of AaCas12b and *A. acidoterrestris* Cas12b (AacCas12b) (PDB: 5WQE). **b** In vitro DNA cleavage analysis of mutation of indicated nuclease mutant or wild-type (WT) AaCas12b proteins. The cleavage rate is shown under the cleaved lanes. **c** T7EI analysis showing effects of mutation of AaCas12b catalytic residues on DNA targeting in 293FT cells. The indel rate is shown under the lanes with mutation. GFP, an empty backbone vector without Cas12b protein expression. **d** Schematic illustration of sgRNA scaffold-based recruitment enabling simultaneous activation of independent target genes. The sgRNA construct with MS2 RNA hairpin recruits MCP-VP64 or MCP-VP64-p65-Rta (VPR) to activate endogenous gene expression in human 293FT cells. **e** The sgRNA scaffold recruits MCP-VPR or MCP-VP64 to simultaneously activate endogenous expression of *IL1B* and *HBG1* in human 293FT cells combined with dAaCas12b or dSpCas9 expression, respectively. Cells transfected with only empty vectors were used as control (Ctrl)

We next intended to abolish the nuclease activity of AaCas12b, as the CRISPR effectors with abolished nuclease activity, can be repurposed for broad applications beyond genome editing. Using protein alignment (Supplementary Fig. S7), we identified six potential catalytic residues of AaCas12b in either the REC lobe or the Nuc lobe according to the structural annotations^11,12^. Then we constructed AaCas12b variants containing point mutations of these residues and tested their cleavage activity in mammalian cells (Fig. 4a). Three AaCas12b variants with catalytic residue mutation (R785A, R911A, and D977A) lost the ability to cleave dsDNAs in vitro, whereas three variants (R122A, D570A, and D806A) still partially retained the catalytic activities (Fig. 4b and Supplementary Fig. S8a). Subsequently, the T7EI assay showed that the three AaCas12b variants (R785A, R911A, and D977A) lost nuclease activities in human cells (Fig. 4c and Supplementary Fig. S8b). Furthermore, in vitro cleavage assays showed that AaCas12b(R785A) could not cleave the Nb.BtsI- and Nt.BstBNI-nicked double-stranded DNA templates, suggesting that the R785A mutation converted AaCas12b nuclease into a deactivated form (dAaCas12b) rather than a nickase (Supplementary Fig. S8c). As a proof-of-concept, we explored the potential of applying the dAaCas12b to targeted gene activation. After co-transfection of dAaCas12b, sgRNAs with MS2 fusion, and the MCP-VP64 or MCP-VPR (VP64-p65-Rta fusion) effectors in 293FT cells^22–25^ (Fig. 4d), the expression levels of two target genes, the *IL1B* and *HBG1* (Supplementary Table S3), were substantially elevated (Fig. 4e), proving the feasibility of dAaCas12b-mediated target gene activation. However, the gene activation level induced by dAaCas12b was still lower that by dSpCas9, suggesting that further improvement of the dAaCas12b-based gene activation platform is required (Fig. 4e).

### Targeted mutagenesis in mice by microinjection of AaCas12b RNPs

**Fig. 5:**
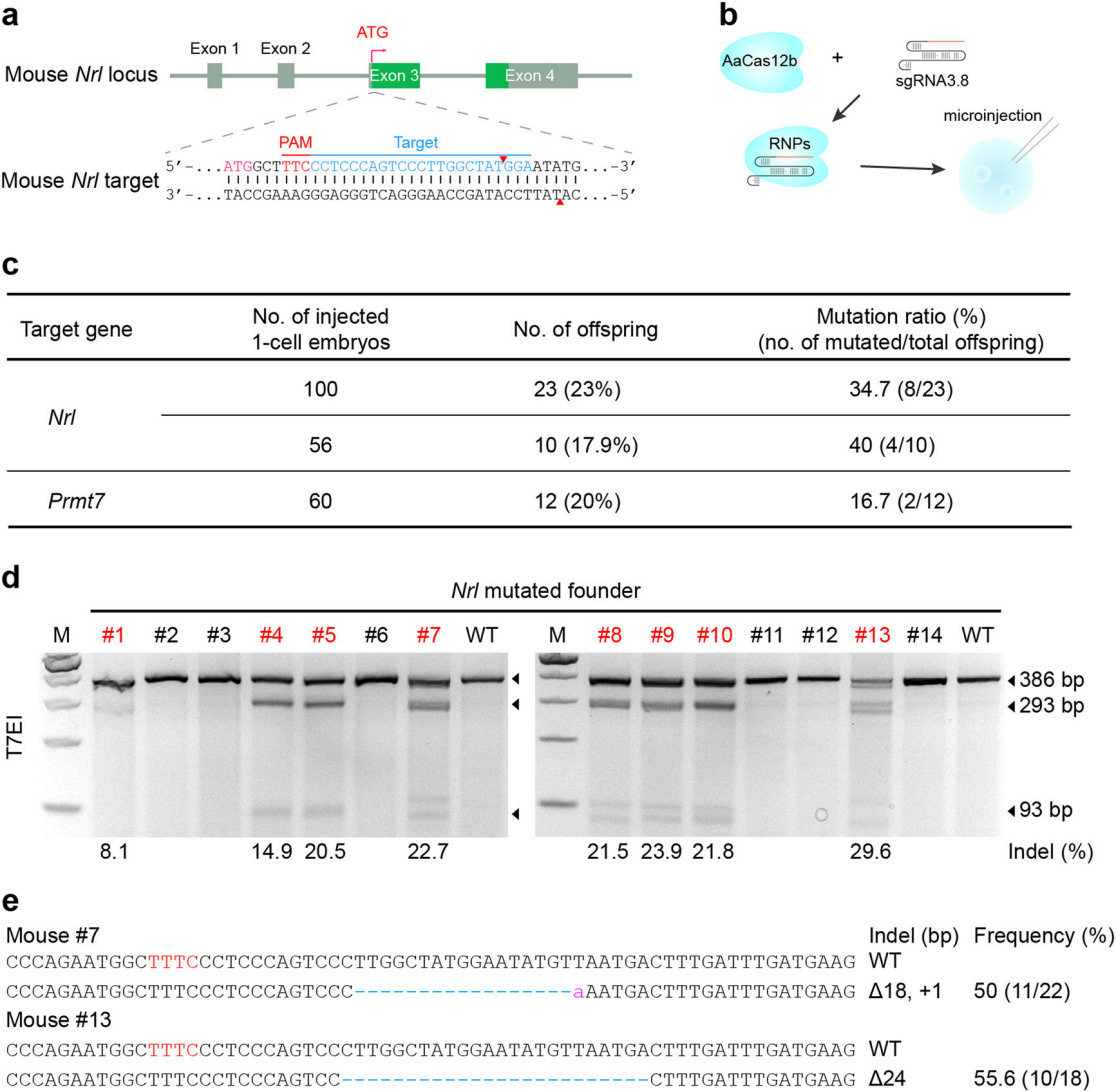
AaCas12b-mediated genome editing in mice. **a** Schematic illustration of AaCas12b sgRNA targeting exon 3 of the mouse *Nrl* gene. The PAM and target sequences are colored in red and blue, respectively. The cleavage sites are indicated with red triangles. **b** Schematic illustration of AaCas12b RNP delivery into 1-cell stage mouse embryos by microinjection. **c** Summary of the mutants generated via AaCas12b RNP microinjection with sgRNA targeting the *Nrl* and *Prmt7* genes, respectively. **d** T7EI-based genotyping assay identifying founder mice derived from embryos injected with AaCas12b RNPs targeting the mouse *Nrl* gene. The numbers in red denote newborn mice with induced indel mutations. The indel rate is shown under the lanes with mutation. **e** Mutated *Nrl* alleles observed in the founder mice in Fig. 5d. Blue dashes, deleted bases; purple lowercases, insertions or mutations; red uppercases, PAM. Indel frequencies are indicated

We next explored the potential of Cas12b in generation of targeted mutations in a whole organism. To generate mutated mice with the AaCas12b system, we conducted embryo microinjection of recombinant AaCas12b RNPs. First, we assessed the activity of preassembled AaCas12b RNPs in mouse embryos at target site 1 in the mouse *Nrl* gene (Fig. 5a). We microinjected the AaCas12b RNPs into 16 one-cell-stage mouse embryos (Fig. 5b), cultured them in vitro and obtained eight blastocysts (Supplementary Fig. S9a). T7EI assay and subsequent Sanger sequencing results showed that six out of eight (75%) blastocysts carried AaCas12b-medaited mutations in the *Nrl* gene (Supplementary Fig. S9b, c). To generate knockout mice, we further transplanted one-cell-stage embryos microinjected with the AaCas12b RNPs into surrogate mothers, and obtained 12 *Nrl* mutant mice and 2 *Prmt7* mutant mice in total as determined by T7EI assay (Fig. 5c, d and Supplementary Fig. S10a, b). Subsequently, Sanger sequencing analysis validated that most mutations were indels with the frequency at up to 66.7% (Fig. 5e, and Supplementary Fig. S10c, d).

Successful germline transmission is a trademark for establishing genetically-modified animal models, particularly for CRISPR-based methods. To assess the transmission of genetic mutations in *Nrl* knockout founders, we analyzed the genotypes of offspring produced from *Nrl* mutants crossed with wild-type (WT) mice. T7EI and Sanger sequencing results demonstrated that the gene mutations in the founders could be successfully transmitted to their offspring (Supplementary Fig. S11). Collectively, these results demonstrate that Cas12b-based genome editing is a useful tool for generating knockout mouse models.

### Minimal off-target effects of AaCas12b in cells and mice

**Fig. 6:**
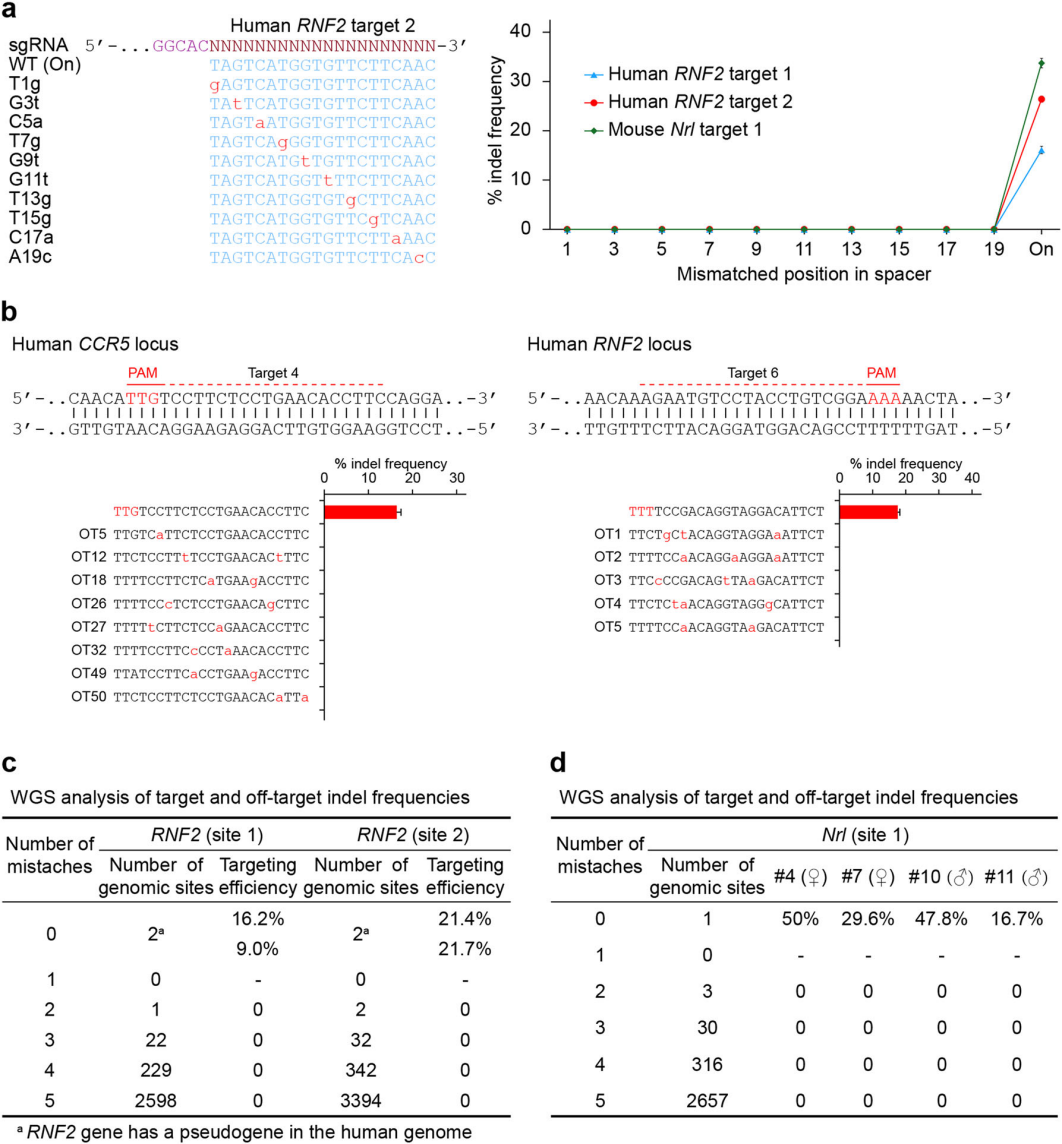
Cleavage specificity and off-target effects of AaCas12b in mammalian genomes. **a** Analysis of cleavage specificity of AaCas12b/sgRNA in human and mouse cells using sgRNAs carrying single base-pair mismatches in the guide sequence. Error bars indicate standard errors of the mean (s.e.m.), *n* = 3. **b** (Top) Schematic illustration of AaCas12b target sites in the human *CCR5* and *RNF2* loci, respectively. (Bottom) Indel frequencies induced by AaCas12b directed by sgRNAs targeting endogenous *CCR5* and *RNF2* sites and their corresponding off-target sites in human 293FT cells. Mutation frequencies were assessed by T7EI assay. Error bars indicate s.e.m., *n* = 2. **c** WGS analysis of genomic DNAs of *RNF2*-targeted 293FT cells. None of 2598 (*RNF2*-site 1) and 3394 (*RNF2*-site 2) sites identified with Cas-OFFinder in the reference genome (*hg38*) that differed from the on-target site by up to five mismatches harbored indels in the mutated genome. **d** Whole-genome sequencing (WGS) analysis of genomic DNAs of *Nrl*-mutated mouse gonads. None of the 2657 sites identified using Cas-OFFinder in the reference genome (*mm10*) that differed from the on-target site by up to five mismatches harbored indels in the mutated genome

Off-target effects pose a major challenge to the applications of genome editing tools. Next, we assessed the off-target effects of AaCas12b-mediated genome editing in mammalian cells. We found that at least 18 nt of the guide sequence was required to produce detectable indels, and a minimum of 20 nt of the guide sequence was required to achieve efficient mutagenesis in human and mouse genomes (Supplementary Fig. S12a). To further test the sensitivity of AaCas12b to Watson–Crick mismatches at the sgRNA-DNA interface, we used sgRNAs containing single substitutions or adjacent double substitutions at the position within 19 nt proximal to the PAM sequence to target the human *RNF2* and mouse *Nrl* genes (Supplementary Table S3). No off-target mutation by any mismatched sgRNAs was detected in these genome loci (Fig. 6a and Supplementary Fig. S12b), suggesting that AaCas12b could hardly tolerate any mismatches between sgRNA and target DNA sequences. To directly assess the off-target mutagenesis in the genome induced by AaCas12b, the potential off-target sites containing one, two, or three mismatches to the guide RNAs were computationally identified from the whole human genome (Supplementary Table S4). No off-target mutation in the 58 predicted genomic loci was detected in the AaCas12b group as shown by T7EI assay and targeted deep sequencing results (Fig. 6b and Supplementary Table S4). We next assessed the degree of genome-wide off-target mutagenesis in 293FT cells targeted with AaCas12b. By performing whole-genome sequencing (WGS) of two mutant cell lines targeted by AaCas12b complexed with sgRNAs, we detected no off-target effects induced by AaCas12b/sgRNA targeting the *RNF2* sites in the human genome (Fig. 6c). These results indicated that AaCas12b had a minimal off-target effect, consistent with the notion of a previous report^12^.

To further determine the specificity of AaCas12b RNPs in gene targeting in vivo, we assessed the potential off-target mutations in the *Nrl*-mutated founder mice. Potential off-targets were predicted in the mouse whole genome with similarity to the target sequence in *Nrl* gene (Supplementary Table S4). Five mutated founders and two WT siblings were analyzed by the T7EI assay and deep sequencing (Supplementary Fig. S12c, d). We found no detectable off-target effects at these 30 potential off-target sites in the five mutants (Supplementary Fig. S12c, d and Supplementary Table S4). WGS data further validated that no off-target effects were induced by AaCas12b RNPs in mice (Fig. 6d and Supplementary Fig. S13). These results demonstrated AaCas12b owned a high cleavage specificity and had minimal off-target effects in both mammalian cells and in mice.

### Fewer off-target effects of AaCas12b compared with SpCas9

**Fig. 7:**
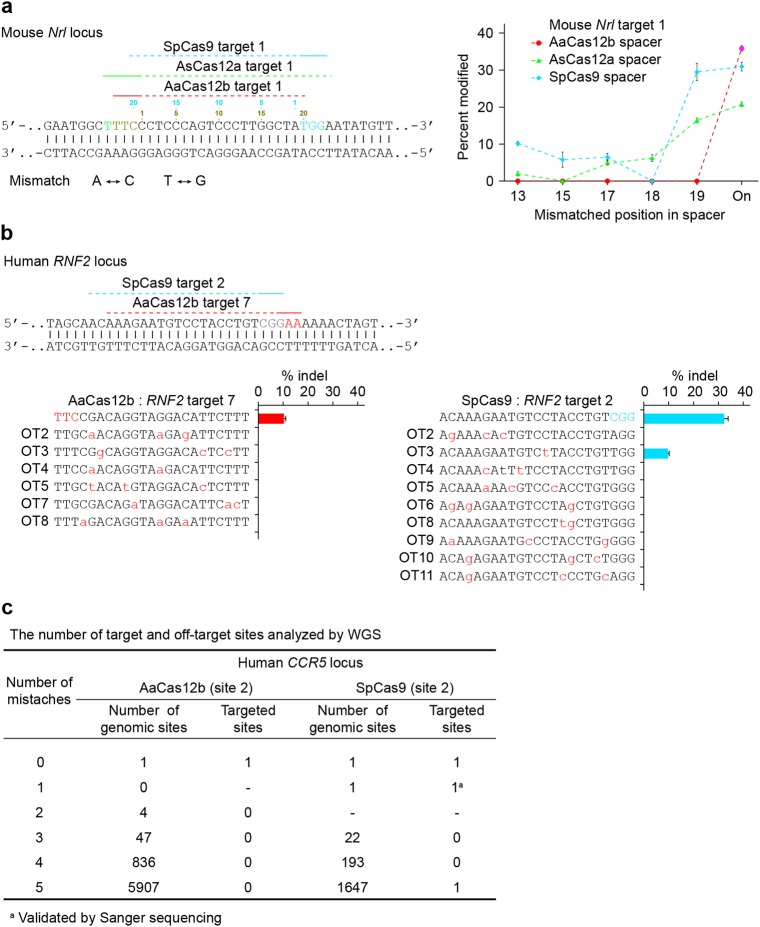
Off-target effects induced by AaCas12b, AsCas12a and SpCas9 in mammalian genomes. **a** (Left) Schematic showing the targeting sequences of AaCas12b, AsCas12a and SpCas9 in mouse *Nrl* locus. (Right) Activities of AaCas12b, AsCas12a and SpCas9 targeted to mouse *Nrl* locus using respective guide RNAs with single mismatches in mouse ES cells. Mutation frequencies were assessed by T7EI assay. Error bars indicate standard errors of the mean (s.e.m.), *n* = 3. **b** (Upper) Schematic showing AaCas12b and SpCas9 targeting sites in the human *RNF2* locus. (*Lower*) Frequencies of induced indels induced at on- and off-target sites by AaCas12b and SpCas9 in human 293FT cells. Mutation frequencies were assessed by T7EI assay. Error bars indicate s.e.m., *n* = 2. **c** Whole-genome sequencing (WGS) analysis potential off-target sites with one to five mismatches to gRNAs induced by AaCas12b or SpCas9 in the human genome and the amount of mutated sites observed by WGS

Next, we sought to directly compare the off-target effects of AaCas12b, SpCas9, and AsCas12a. We used guide RNAs (gRNAs) containing single mismatches within 19 nt from the PAMs to target the same genomic loci (Supplementary Table S3). Both SpCas9 and AsCas12a generated substantial mutations with mismatched gRNAs in mouse and human cells (Fig. 7a and Supplementary Fig. S14). However, AaCas12b did not generate any mutations with mismatched gRNAs (Fig. 7a and Supplementary Fig. S14), suggesting the requirement of a perfect match between the sgRNA and target sequence for the AaCas12b-mediated mammalian genome editing. To directly assess the off-target mutagenesis in the genome induced by AaCas12b and SpCas9, the potential off-target sites, containing one, two, or three mismatches to the gRNAs, were computationally identified from the whole human genome^26^ (Supplementary Table S5). No off-target mutation in the 88 predicted genomic loci was detected in the AaCas12b group as T7EI results showed (Fig. 7b, Supplementary Fig. S15 and Supplementary Table S5). However, 3 out of 82 predicted off-target sites in the SpCas9 group were mutated (Fig. 7b, Supplementary Fig. S15 and Supplementary Table S5). We also performed whole-genome sequencing (WGS) analysis. Two mutated off-targets were induced by SpCas9 when targeting *CCR5* gene in the human genome, however, no off-target mutation was detected by AaCas12b (Fig. 7c). These results indicated that AaCas12b possessed fewer off-target effects than SpCas9.

## Discussion

In this work, we demonstrated that type V-B CRISPR-Cas12b systems can enable robust engineering of mammalian genomes. Similar to Cas9, Cas12b is a dual-RNA-guided endonuclease^10^, in contrast to the single-RNA-guided Cas12a^9^. We showed that the crRNA/tracrRNA duplex can be engineered into a shorter chimeric RNA, which works as efficiently as the dual-RNA duplex. Also, we demonstrated that the 5′-end and the stem-loop 3 can be programmed as a scaffold to recruit effector proteins, together with the dAaCas12b, suggesting that the AaCas12b system can be repurposed for versatile applications, such as regulation of endogenous gene expression. Additionally, Cas12b generates a staggered DSB with a 5′ overhang, which is similar to Cas12a^9^. This structure of the cleavage products might be advantageous for facilitating non-homologous end joining (NHEJ)-based gene modifications^27^, particularly in nondividing cells^28^.

Importantly, we showed that AaCas12b RNPs can efficiently introduce targeted indel mutations in mouse embryos, which can be successfully transmitted into the next generation via germline. Our results also indicated that AaCas12b possess minimal off-target effects in cell lines compared with SpCas9 and AsCas12a. Through thorough assays by targeted deep sequencing and WGS, we identified no off-target effects in both cell lines and mice. This high specificity indicates that AaCas12b will be useful for therapeutic applications, though more detailed investigation is required.

Another potential promising feature of CRISPR-Cas12b is that AaCas12b possesses its nuclease activity over a wide range of temperatures and pH values, which will enhance the utility of CRISPR-Cas12b technology under both thermophilic and acidophilic conditions. Moreover, we have shown that AaCas12b possesses longer lifetime and improved stability as RNPs in human plasma, which will benefit the future clinical applications requiring delivery of Cas12b into the bloodstream.

## Materials and methods

### DNA manipulations

DNA manipulations, including DNA preparation, digestion, ligation, amplification, purification, agarose gel electrophoresis, etc. were conducted according to *Molecular Cloning: A Laboratory Manual* with some modifications. Briefly, PAM determination plasmids were constructed by ligating annealed oligonucleotides (oligos) (Supplementary Table S1) into the p11-LacY-wtx1 vector^29^ digested by EcoRI and SphI, and corresponding dsDNA fragments were PCR-generated (Supplementary Table S6). Targeting crRNA/tracrRNA duplexes or sgRNAs (thereafter guide RNAs or gRNAs) for cell transfection assay were constructed by ligating annealed oligos (Supplementary Table S3) into BasI-digested pUC19-U6-gRNA vectors (Supplementary Sequences). Templates for in vitro transcription of tracrRNAs and sgRNAs were PCR-amplified using primers containing a T7 promoter sequence (Supplementary Table S2 and S6). The crRNAs were transcribed from annealed oligos bearing a T7 promoter (Supplementary Table S1).

### De novo gene synthesis and plasmid construction

PSI-BLAST program^30^ were adopted to identify new type V-B CRISPR-Cas12b proteins. And their coding sequences were humanized^31^, and oligos for the synthetic Cas12b genes and their cognate gRNAs were designed by GeneDesign program^32^. For Cas12b coding genes which are > 3 kb were split into four “chunks” of ~800 bp (Supplementary Table S1 and Supplementary Sequences). For gRNAs which are < 300 bp were left as is (Supplementary Table S1). All oligos for gene synthesis were commercially purchased (Taihe Biotechnology Co., LTD). Oligos within one chunk were mixed into the final concentration at 300 nM and applied for fragment assembly without primers in a 25 μL PCR amplification system. Then a 2.5-μL product from the first-round PCR was used as a template with 2.5 μL, 3 μM primers added to amplify the designed fragments in a 25-μL system. The route of 1st and 2nd PCR-based gene assembly reaction: 95 °C 15 min; 95 °C 30 s, 59 °C 30 s, 72 °C 30 s/500 bp, 25 cycles; 72 °C 10 min^33^. Purified products were assembled into expression vectors via homologous recombination *i*n vitro using NEBuilder^®^ HiFi DNA Assembly Master Mix (NEB). The pCAG-2AeGFP vector (Supplementary Sequences) was applied for mammalian cell expression of Cas12b proteins. gRNAs were constructed in the pUC19-U6 vector (Supplementary Sequences) for mammalian cell expression.

### Protein purification

The synthetic Cas12b coding sequences were constructed into a BPK2014-*ccdB* expression vector (Supplementary Sequences) using ligation-dependent cloning. The resulting fusion construct containing a C-terminal fused His_10_ tag. The proteins were expressed in *E. coli* strain BL21 (λDE3), grown in Cm^R^ + LB medium at 37 °C to OD_600_ ~0.4, following induction with 0.5 mM IPTG at 16 °C for 16 h. In total, 300mL of induced cells were harvested for protein purification, and all subsequent steps were conducted at 4 °C. Cell pellets were lysed in 30 mL Lysis Buffer (NPI-10: 50 mM NaH_2_PO_4_, 300 mM NaCl, 10 mM imidazole, 5% glycerol, pH 8.0) supplemented with 1x protease inhibitors (Roche cOmplete, EDTA-free) before lysis by sonication. Lysates were clarified by centrifugation at 8000 rpm, 4 °C for 10 min, and the supernatants were incubated with His60 Ni Superflow Resin (Takara) in batch at 4 °C for 2 h. After the Resin was washed with each 20 mL Wash Buffer 1 (NPI-20: 50 mM NaH_2_PO_4_, 300 mM NaCl, 20 mM imidazole, 5% glycerol, pH 8.0), Wash Buffer 2 (NPI-40: 50 mM NaH_2_PO_4_, 300 mM NaCl, 40 mM imidazole, 5% glycerol, pH 8.0) and Wash Buffer 3 (NPI-100: 50 mM NaH_2_PO_4_, 300 mM NaCl, 100 mM imidazole, 5% glycerol, pH 8.0), expressed proteins were eluted with 5 mL Elution Buffer (NPI-500: 50 mM NaH_2_PO_4_, 300 mM NaCl, 500 mM imidazole, 5% glycerol, pH 8.0). Purified Cas12b proteins were dialyzed using 100 kDa dialyzer overnight with storage buffer (50 mM Tris-HCl, 200 mM KCl, 0.1 mM EDTA, 1 mM DTT, 20% glycerol, pH 8.0). Fractions were pooled and concentrated with 100 kDa Centrifugal Filter Unit (Millipore). The purity of enriched proteins was analyzed by SDS-PAGE and Coomassie staining, and the concentration was quantitated using BCA Protein Assay Kit (Thermo Fisher).

### In vitro RNA transcription

RNAs were in vitro-transcribed using HiSribe^TM^ T7 Quick High Yield RNA Synthesis Kit (NEB) and PCR-amplified DNA templates carrying a T7 promoter sequence. Transcribed RNAs were purified using Oligo Clean & Concentrator^TM^ (ZYMO Research) and quantitated on NanoDrop^TM^ 2000 (Thermo Fisher). Primers used for template preparation are listed in Supplementary Table S6.

### In vitro analysis of PAM sequences

To determine the PAM sequence of Cas12b, 100 nM Cas12b proteins, 400 ng in vitro-transcribed sgRNAs and 200 ng PCR-generated dsDNAs bearing different PAM sequences (Supplementary Table S2) were incubated at 37 °C for 1 h in the cleavage buffer (50 mM Tris-HCl, 100 mM NaCl, 10 mM MgCl_2_, pH 8.0). The reactions were stopped by adding RNase A to digest gRNAs at 37 °C for 20 min, and resolved by ~3% agarose gel electrophoresis and ethidium bromide staining.

### dsDNA cleavage assay

For dsDNA cleavage assay, 100 nM Cas12b proteins, 400 ng in vitro-transcribed gRNAs and 200 ng PCR-generated dsDNAs containing a 5′-TTN PAM sequence was conducted at 37 °C for 1 h in cleavage buffer (50 mM Tris-HCl, 100 mM NaCl, 10 mM MgCl_2_, pH 8.0), if not specified. To determine the thermostability of AaCas12b, the cleavage was reacted at a large-range temperature (4 °C – 100 °C) for 1 h in cleavage buffer (50 mM Tris-HCl, 100 mM NaCl, 10 mM MgCl_2_, pH 8.0). For pH tolerance assay, the cleavage reactions were performed at 37 °C for 1 h in cleavage buffer (50 mM Tris-HCl, 100 mM NaCl, 10 mM MgCl_2_) with pH value ranging from 1.0 to 13.0. In Mg^2+^-dependent assay, cleavage buffer (50 mM Tris-HCl, 100 mM NaCl, pH 8.0) was supplemented with EDTA (0 mM, 1 mM, 5 mM, 10 mM, 20 mN, and 40 mM) or Mg^2+^ (0 mM, 1 mM, 5 mM, 10 mM, 20 mN, and 40 mM), and the mixture was incubated at 37 °C for 1 h. Further metal-dependent cleavage reactions were conducted at 37 °C for 1 h in cleavage buffer (50 mM Tris-HCl, 100 mM NaCl, 10 mM MgCl_2_, 1 mM EDTA, pH 8.0) supplemented with 1 or 5 mM of CaCl_2_, MnCl_2_, SrCl_2_, NiCl_2_, FeCl_2_, CoCl_2_, ZnCl_2_, or CuCl_2_. The reactions were stopped by adding RNase A to digest sgRNAs at 37 °C for 20 min, and resolved by ~3% agarose gel electrophoresis and ethidium bromide staining.

### Thermostability assay

SpCas9 and AaCas12b nucleases were incubated in diluted concentrations of human plasma at 37 °C for 12 h. After incubation, DNA cleavage reactions were conducted as described above. For SpCas9, its target bears a 3′-NGG PAM. The reactions were stopped by adding RNase A to digest sgRNAs at 37 °C for 20 min, and resolved by ~3% agarose gel electrophoresis and ethidium bromide staining.

### Cell culture, transfection, and fluorescence-activated cell sorting (FACS)

Human embryonic kidney cell line 293FT and HeLa cells were maintained in Dulbecco’s Modified Eagle’s Medium (DMEM, Gibco) supplemented with 10% fetal bovine serum (FBS, Gibco) and 1% antibiotic–antimycotic (Gibco) at 37℃ with 5% CO_2_ incubation. Mouse embryonic stem (mES) cell line was maintained in N2B27 medium plus 1 μM PD0325901 (Stemgent), 3 μM CHIR99021 (Stemgent), and 1000 U/mL mLIF (Millipore). The N2B27 medium consists of DMEM/F12 (Gibco) and Neurobasal (Gibco) at a ratio of 1:1 and was supplemented with 1% N-2 supplement (Gibco), 0.5% B-27 supplement (Gibco), 20 ng/ml BSA (Sigma), 10 μg/ml insulin (Roche Applied Science), 1% GlutaMAX (Gibco), 5% knockout serum replacement (KOSR, Gibco), 0.1% 2-mercaptoethanol (Gibco) and 1% antibiotic–antimycotic (Gibco). 293FT cells were transfected using Lipofectamine LTX (Invitrogen) following the manufacturer’s recommended protocol. mES cells were transfected via electroporation using Neon^TM^ transfection system (Invitrogen) following the manufacturer’s recommended protocol. For each well of a 24-well-plate, a total of 750 ng plasmids (Cas12b: gRNA = 2: 1) were used. Then 48 h following transfection, GFP-positive cells were sorted using the MoFlo XDP (Beckman Coulter).

### T7 endonuclease I (T7EI) assay and Sanger sequencing analysis for genomic modifications

Harvested or FACS-sorted GFP-positive 293FT or mES cells post transfection were lysed for genomic DNA extraction. Briefly, cells were directly lysed with Buffer L and incubated at 55 °C for 3 h and 95 °C for 10 min. Genomic region surrounding the CRISPR-Cas12b target site for each gene was PCR-amplified (Supplementary Table S6). 200– 400 ng PCR products were mixed with ddH_2_O to a final volume of 10 μL, and subjected to re-annealing process to enable heteroduplex formation according to previous methods^6^. After re-annealing, products were treated with 1/10 volume of NEBuffer^TM^ 2.1 and 0.2 μL T7EI (NEB) at 37 °C for 30 min, and analyzed on 3% agarose gels. Indels were quantitated based on relative band intensities^6^. T7EI assay-identified mutated products were subjected to be cloned into TA-cloning vector and transformed into competent *E. coli* strain. After overnight culture, colonies were randomly picked out and sequenced.

### Site-directed Cas12b gene mutagenesis

Two pairs of primers containing the desired site-directed mutations and 5′-end overlaps were used for gene amplification (Supplementary Table S6). The two agarose gel-purified gene fragments were seamlessly assembled into XmaI and NheI double-digested mammalian expression vector (Supplementary Sequences) using NEBuilder^®^ HiFi DNA Assembly Master Mix (NEB) following the manufacture’s recommended protocol. And *E. coli* expression vectors were reconstructed using digestion- and ligation-dependent methods.

### Endogenous gene transcription activation

For dAaCas12b-based transcription activation of endogenous genes, dAaCas12b, targeting sgRNAs bearing MS2 RNA hairpin, and MCP-VP64 or MCP-VPR (VP64-p65-Rta fusion) were co-transfected into human 293FT cells. For each gene, eight target sgRNAs were constructed and mixed into a pool (Supplementary Table S3). All target sites were located within the ~ 500 bp upstream of the transcription start site (TSS).

### Animals

Mice were housed in the animal care facility of the Institute of Zoology, Chinese Academy of Sciences, according to the institutional guidelines for the care and use of laboratory animals. Specific pathogen-free (SPF) grade ICR mice (Stock No. 201) were purchased from Beijing Vital River Laboratory Animal Technology Co., Ltd. All animal experiments were conducted according to the guidelines for the care and use of laboratory animals established by the Beijing Association for Laboratory Animal Science and approved under the Animal Ethics Committee of the Institute of Zoology, Chinese Academy of Sciences (1 Beichen West Road, Chaoyang District, Beijing, P.R. China).

### Microinjection of AaCas12b RNPs

ICR female mice at 8 weeks of age were superovulated by intraperitoneal injection of PMSG and hCG hormones (Sigma) at a 48 -h interval. These mice were mated with ICR male mice at 10 to 16 weeks of age, and fertilized one-cell embryos were collected from the oviduct. Cumulus cells were removed from embryos by exposure to 0.1% hyaluronidase (Sigma) in PBS buffer. For microinjection, preassembled AaCas12b RNPs were injected into cytoplasm using a Nikon ECLIPSE T*i* micromanipulator and a *FemtoJet 4i* microinjector (Eppendorf). Embryos were cultured in microdrops of KSOM + AA containing D-glucose and phenol red (Millipore) under mineral oil at 37 °C for 3.5 days in a humidified atmosphere consisting of 5% CO_2_ in air. Two-cell stage embryos were transferred on the following day into the oviducts of 0.5 dpc pseudopregnant foster mothers to obtained mutated founders.

### Off-target prediction and detection

The potential off-target sites for CRISPR-Cas12b system in the human or mouse genome with individual on-target sequences were predicted using Cas-OFFinder^26^. The complementarity region bearing one, two, or three mismatches with requisite PAMs were assessed as potential off-targets by T7EI assay or targeted deep sequencing.

### Targeted deep sequencing

Target sites and potential off-target sites were amplified by barcoded PCR and pooled libraries were subjected to paired-end sequencing using HiSeq (Illumina). A reference genome was built using Picard Tools (http://broadinstitute.github.io/picard) and samtools^34^ from DNA sequences of the considered on-/off-target regions. Raw sequencing data (FASTQ files) were mapped against the created reference genone using BWA^35^ with standard parameters and resulting alignment files were sorted using smatools. Samples with fewer than 20 reads were excluded.

### Whole-genome sequencing (WGS)

Genomic DNA from cultured cells or whole gonad tissues were extracted using MicroElute Genomic DNA Kit (OMEGA) and subjected to quality assessment. The extracted DNA was sequenced using an Illumina NovaSeq sequencer at a sequencing depth of 30x diploid coverage. The pair-ends reads were aligned onto the *hg19* (GRCh38) human or *mm10* (GRCm38) mouse reference genome using Bowtie 2^36^ and BWA^35^, following manipulated using Picard Tools (http://broadinstitute.github.io/picard), respectively.

The WGS analysis was performed according to previous report^37^. Briefly, the Genome Analysis ToolKit (GATK4)^38^ or pysamstats (https://github.com/alimanfoo/pysamstats) were used for local realignment around indels, base score recalibration, variant calling across the human or mouse samples, and variant score recalibration. Candidate indels were filtered on several criteria using Python, PyVCF, and PyFasta packages. First, we removed indels near low-complexity regions as defined by RepeatMasker (http://repeatmasker.org) and annotated by softmasking in *hg19* or *mm10*. Second, we removed indels that caused expansions or compressions of long (one with > 6 bp or two with > = 5 bp) homopolymers.

## Electronic supplementary material

Supplementary Information

Supplementary Table S1

Supplementary Table S2

Supplementary Table S3

Supplementary Table S4

Supplementary Table S5

Supplementary Table S6

## Electronic supplementary material

**Supplementary Information** accompanies the paper at (10.1038/s41421-018-0069-3).
