# Supplementary material for: Repurposing CRISPR-Cas12b for mammalian genome engineering
Source: Cell Discov. 2018 Nov 27;4:63. doi: 10.1038/s41421-018-0069-3 (PMC6255809; doi:10.1038/s41421-018-0069-3)
Supplement: Supplementary file 1 — Supplementary Information [file 41421_2018_69_MOESM1_ESM.pdf]

Figure S1

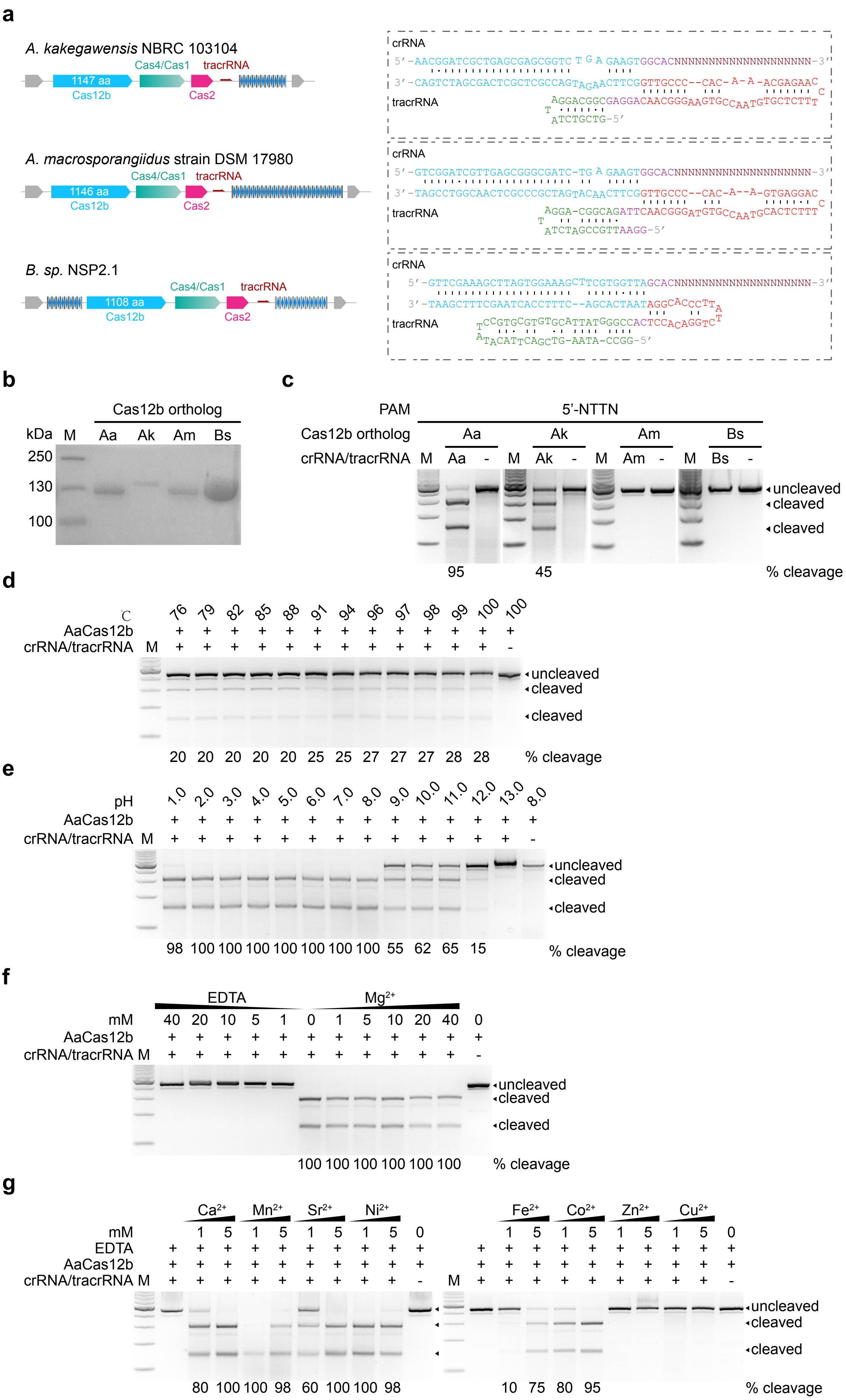

a
A. kakegawensis NBRC 103104
1147 aa
Cas12b
Cas4/Cas1
Cas2
tracrRNA
A. macrosporangiidus strain DSM 17980
1146 aa
B. sp. NSP2.1
1108 aa
crRNA
tracrRNA
b
Cas12b ortholog
kDa M Aa Ak Am Bs
250
130
100
c
PAM 5'-NTTN
Cas12b ortholog Aa Ak Am Bs
crRNA/tracrRNA M Aa - M Ak - M Am - M Bs -
uncleaved
cleaved
cleaved
95 45 % cleavage
d
°C 76 79 82 85 88 91 94 96 97 98 99 100 100
AaCas12b
crRNA/tracrRNA M
20 20 20 20 20 25 25 27 27 27 28 28 % cleavage
e
pH 1.0 2.0 3.0 4.0 5.0 6.0 7.0 8.0 9.0 10.0 11.0 12.0 13.0 8.0
98 100 100 100 100 100 100 100 55 62 65 15 % cleavage
f
EDTA
Mg2+
mM 40 20 10 5 1 0 1 5 10 20 40 0
100 100 100 100 100 100 % cleavage
g
Ca2+ Mn2+ Sr2+ Ni2+
Fe2+ Co2+ Zn2+ Cu2+
mM
EDTA
AaCas12b
crRNA/tracrRNA M
80 100 100 98 60 100 100 98
10 75 80 95 % cleavage

h

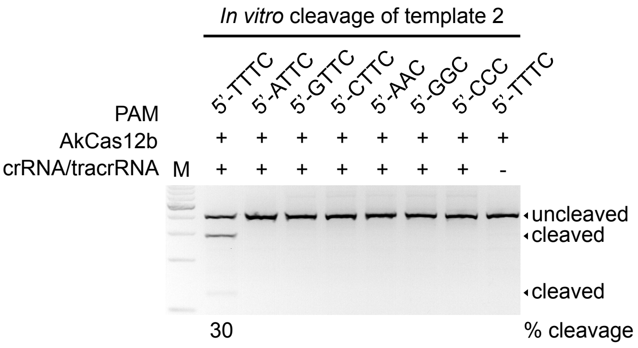

In vitro cleavage of template 2
PAM
AkCas12b
crRNA/tracrRNA
M
5'-TTTC
5'-ATTC
5'-GTTC
5'-CTTC
5'-AAC
5'-GGC
5'-CCC
5'-TTTC
+ + + + + + + +
+ + + + + + + -
uncleaved
cleaved
cleaved
30
% cleavage

i

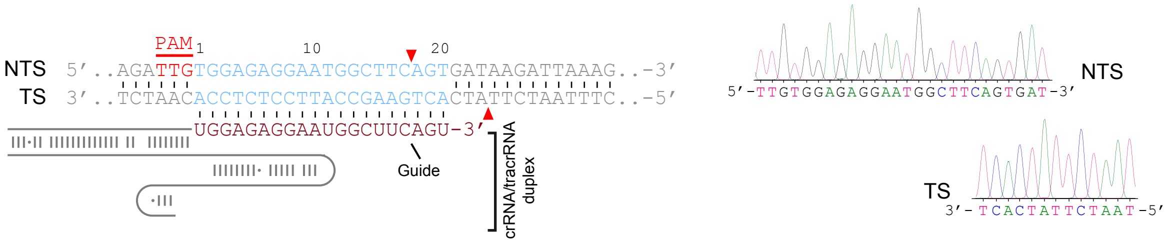

PAM
1
10
20
NTS 5'..AGATTGTGGAGAGGAATGGCTTCAGTGATAAGATTAAAG..-3'
TS 3'..TCTAACACCTCTCCTTACCGAAGTCACTATTCTAATTTC..-5'
UGGAGAGGAAUGGCUUCAGU-3'
Guide
crRNA/tracrRNA duplex
5'-TTGTGGAGAGGAATGGCTTCAGTGAT-3'
NTS
TS
3'-TCACTATTCTAAT-5'

Fig. S1. *In vitro* DNA cleavage assay of Cas12b proteins. a Schematic ilustration of the genomic architecture of CRISPR-Cas12b from *Alicyclobacillus kakegawensis* (NBRC 103104), *Alicyclobacillus macrosporangiidus* (strain DSM 17980), *Bacillus sp. (NSP2.1)* (*left*), and the crRNA/tracrRNA duplex from each strain (*right*). **b** Coomassie blue staining of AaCas12b, AkCas12b, AmCas12b and BsCas12b purified from *E. coli*. **c** *In vitro* cleavage of double-stranded DNAs containing the 5'-NTTN PAMs by the purified AaCas12b, AkCas12b, AmCas12b and BsCas12b proteins and their cognate crRNA/tracrRNA duplexes. The cleavage rate is shown under the cleaved lanes. **d** *In vitro* cleavage activity of AaCas12b at various temperatures. The cleavage rate is shown under the cleaved lanes. **e** *In vitro* cleavage activity of AaCas12b under various pH conditions. The cleavage rate is shown under the cleaved lanes. **f** *In vitro* cleavage assay showing the Mg^{2+}-dependent endonuclease activity of AaCas12b. The cleavage rate is shown under the cleaved lanes. **g** *In vitro* cleavage assay of AaCas12b in the presence of indicated metals, Ca^{2+}, Mn^{2+}, Sr^{2+}, Ni^{2+}, Fe^{2+}, Co^{2+}, Zn^{2+}, Cu^{2+}. The cleavage rate is shown under the cleaved lanes. **h** *In vitro* validation of the PAM requirements of AkCas12b showing that PAMs matching the 5'-TTTN sequence can be efficiently cleaved. The cleavage rate is shown under the cleaved lanes. **i** Cleavage site determination of AaCas12b by sequencing the cleavage products. The cleavage sites are indicated by red triangles in the left panel. TS, target strand; NTS, non-target strand.

Figure S2

a

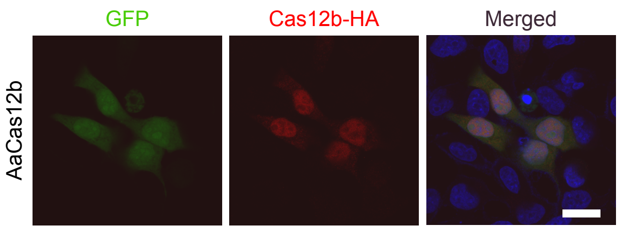

b

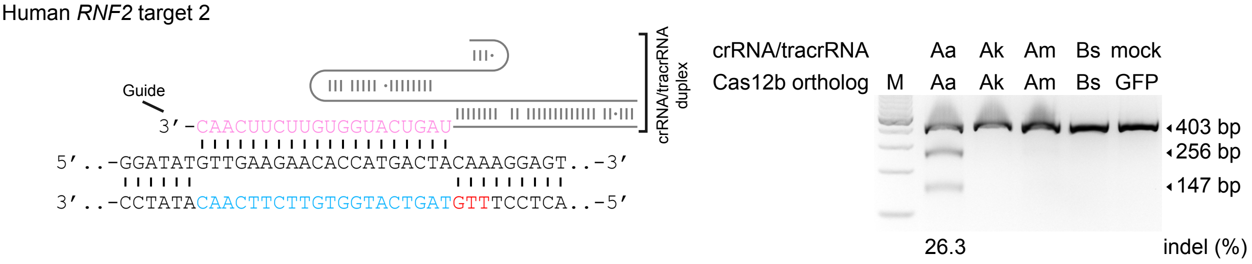

c

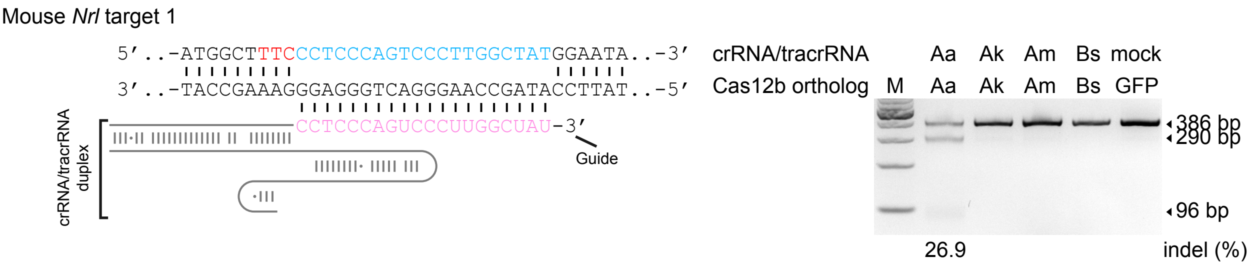

d

Human *RNF2* target 2

```
TGAATTAATGTGCCCAATTTGTTTGGATATGTTGAAGAACACCATGACTACAAAGGAGTGTTTACATCGTT  Wild-type
TGAATTAATGTGCCCAATTTGTTTG----TGTTGAAGAACACCATGACTACAAAGGAGTGTTTACATCGTT  Δ4
TGAATTAATGTGCCCAATTTGTTTGGATA-----AAGAACACCATGACTACAAAGGAGTGTTTACATCGTT  Δ5
TGAATTAATGTGCCCAATTTGTTTGGATATGT-----AACACCATGACTACAAAGGAGTGTTTACATCGTT  Δ5
TGAATTAATGTGCCCAATTTGTTT--------TGAAGAACACCATGACTACAAAGGAGTGTTTACATCGTT  Δ8
TGAATTAATGTGCCCAATTTGTTTGGATA--------AACACCATGACTACAAAGGAGTGTTTACATCGTT  Δ8
TGAATTAATGTGCCCAATTTGTTTGGATA-----------------------------TGTTTACATCGTT  Δ29
```

e

Mouse *Nrl* target 1

```
CTCAGTCCCAGAATGGCTTTCCCTCCCAGTCCCTTG-GCTATGGAATATGTTAATGACTTTGATTTGATGA  Wild-type
CTCAGTCCCAGAATGGCTTTCCCTCCCAGTCCCTTGcGCTATGGAATATGTTAATGACTTTGATTTGATGA  +1
CTCAGTCCCAGAATGGCTTTCCCTCCCAGTCCCTTG-GC--TGGAATATGTTAATGACTTTGATTTGATGA  Δ2
CTCAGTCCCAGAATGGCTTTCCCTCCCAGTCCCTTG-------GAATATGTTAATGACTTTGATTTGATGA  Δ6
CTCAGTCCCAGAATGGCTTTCCCTCCCAGTCCCTT-------------TGTTAATGACTTTGATTTGATGA  Δ12
CTCAGTCCCAGAATGGCTTTCCCTCCCAGTCCCTT--------------GTTAATGACTTTGATTTGATGA  Δ13
CTCAGTCCCAGAATGGCTTTCCCTCCCAGTCCCTT------------------------------TGATGA  Δ29
```

Fig. S2 Cas12b nucleases mediate robust genome editing in mammalian cells. a Immunofluorescence staining showing two nuclear localization signals (NLSs) ensured nuclear compartmentalization of AaCas12b in HeLa cells. Scale bar, 20 μm. **b** (*Left*) Schematic illustration of the human *RNF2* target site 2 of AaCas12b and crRNA/tracrRNA duplex. Red letters indicate the PAM sequence. (*Right*) T7EI analysis of indels produced by Cas12b orthologues (AaCas12b, AkCas12b, AmCas12b and BsCas12b) at the human RNF2 gene target site 2. The indel rate is shown under the lane with mutation. mock, an U6 empty vector without crRNA/tracrRNA expression. GFP, an empty backbone vector without Cas12b protein expression. **c** (*Left*) Schematic illustration of the mouse *Nrl* target site 1 of AaCas12b and crRNA/tracrRNA duplex. Red letters indicate the PAM sequences. (*Right*) T7EI analysis of indels produced by Cas12b orthologues (AaCas12b, AkCas12b, AmCas12b and BsCas12b) at the mouse *Nrl* gene target site 1. The indel rate is shown under the lane with mutation. mock, an U6 empty vector without crRNA/tracrRNA expression. GFP, an empty backbone vector without Cas12b protein expression. **d** Sanger sequencing results showing the indels in human *RNF2* target site 2 produced by AaCas12b. Blue dashes, deleted bases; red uppercases, PAM. **e** Sanger sequencing results showing the indels in mouse *Nrl* target site 1 produced by AaCas12b. Blue dashes, deleted bases; purple lowercases, insertions or mutations; red uppercases, PAM.

Figure S3

Gene	Sequence (5' - 3')	% indel frequency		
		AaCas12b	AsCas12a	SpCas9
CCR5	GAATTCTTTGGCCTGAATAATTGCAGTAGCTCTAACAGGTTGGACCAA	19.4 ± 0.5	21.1 ± 1.0	18.8 ± 0.8
CCR5	TTCTCCTGAACACCTTCCAGGAATTCTTTGGCCTGAATAATTGCAGTA	10.4 ± 0.9	ND	35.1 ± 1.3
DNMT1	GACTTTTCCTTTTATTTCCCTTCAGCTAAAATAAAGGAGGAGGAAGCT	28.1 ± 2.8	20.1 ± 2.0	16.7 ± 1.1
DNMT1	CGTTAATGTTTCCTGATGGTCCATGTCTGTTACTCGCCTGTCAAGTGG	13.0 ± 0.9	15.6 ± 0.8	21.5 ± 1.2
DYRK1A	CATGAGGTGACCCATTTCCATTCAAGGGTTTTAGAAGCACATCAAGGA	22.0 ± 1.8	ND	24.3 ± 1.5
EMX1	CGATGTCACCTCCAATGACTAGGGTGGGCAACCACAAACCCACGAGGG	5.2 ± 0.8	11.9 ± 1.1	20.1 ± 1.3
RNF2	CCCAATTTGTTTGGATATGTTGAAGAACACCATGACTACAAAGGAGTG	26.7 ± 0.4	12.7 ± 2.1	29.5 ± 3.0
Nrl	ATGGCTTTCCCTCCCAGTCCCTTGGCTATGGAATATGTTAATGACTTT	35.9 ± 0.7	20.9 ± 0.6	30.8 ± 1.2

ND, not detectable

Fig. S3 Frequency of AaCas12b-, AsCas12a- and SpCas9-mediated targeted indel mutations at on-target sites in mammalian cells. Target genomic DNA sequences and the resulted indel frequencies of AaCas12b, AsCas12a and SpCas9 are shown. Target sites for AaCas12b, AsCas12a and SpCas9 are shown and underlined in red, green and blue, respectively. Mutation frequencies were assessed by T7EI assay. Error bars indicate standard errors of the mean (s.e.m.), n = 2.

Figure S4

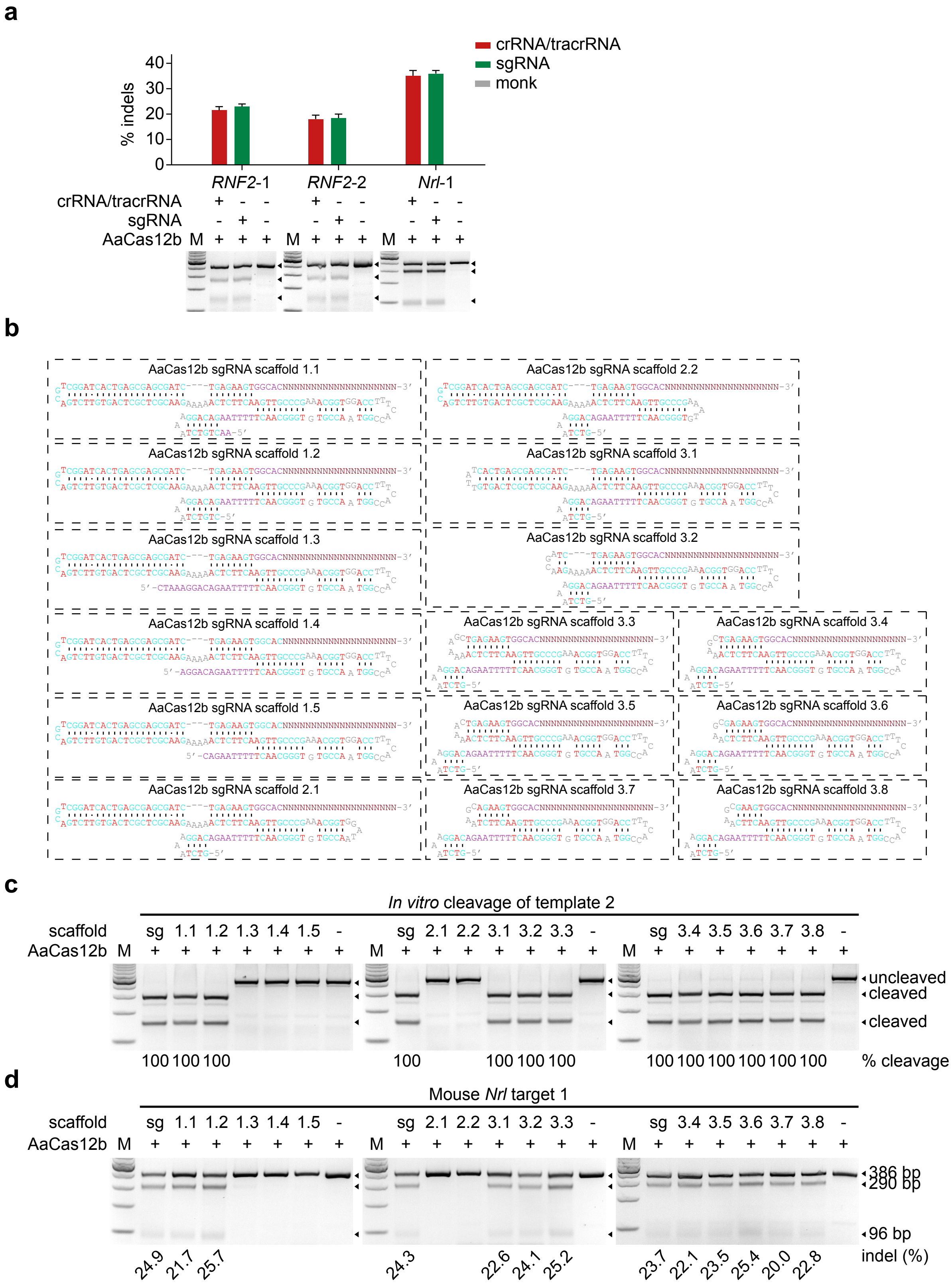

a
crRNA/tracrRNA
sgRNA
monk
% indels
40
30
20
10
0
RNF2-1
RNF2-2
Nrl-1
crRNA/tracrRNA + - - + - - + - -
sgRNA - + - - + - - + -
AaCas12b M + + + M + + + M + + +
b
AaCas12b sgRNA scaffold 1.1
AaCas12b sgRNA scaffold 2.2
AaCas12b sgRNA scaffold 1.2
AaCas12b sgRNA scaffold 3.1
AaCas12b sgRNA scaffold 1.3
AaCas12b sgRNA scaffold 3.2
AaCas12b sgRNA scaffold 1.4
AaCas12b sgRNA scaffold 3.3
AaCas12b sgRNA scaffold 3.4
AaCas12b sgRNA scaffold 1.5
AaCas12b sgRNA scaffold 3.5
AaCas12b sgRNA scaffold 3.6
AaCas12b sgRNA scaffold 2.1
AaCas12b sgRNA scaffold 3.7
AaCas12b sgRNA scaffold 3.8
c
In vitro cleavage of template 2
scaffold sg 1.1 1.2 1.3 1.4 1.5 - sg 2.1 2.2 3.1 3.2 3.3 - sg 3.4 3.5 3.6 3.7 3.8 -
AaCas12b M + + + + + + + M + + + + + + + M + + + + + + +
uncleaved
cleaved
cleaved
100 100 100 100 100 100 100 100 100 100 100 100 100 % cleavage
d
Mouse Nrl target 1
scaffold sg 1.1 1.2 1.3 1.4 1.5 - sg 2.1 2.2 3.1 3.2 3.3 - sg 3.4 3.5 3.6 3.7 3.8 -
AaCas12b M + + + + + + + M + + + + + + + M + + + + + + +
386 bp
290 bp
96 bp
24.9 21.7 25.7 24.3 22.6 24.1 25.2 23.7 22.1 23.5 25.4 20.0 22.8
indel (%)

Fig. S4 Engineered AaCas12b chimeric sgRNAs for genome editing. a Indel frequencies induced by AaCas12b directed by crRNA/tracrRNA duplexes and sgRNAs. Indel frequencies are calculated by T7EI assay. Error bars indicate standard errors of the mean (s.e.m.), n = 3. **b** Schematic illustration of 5' truncated sgRNAs on stem loop 1, 2 and 3. **c** *In vitro* DNA cleavage assay of AaCas12b complexed with the optimized sgRNAs. The cleavage rate is shown under the cleaved lanes. **d** Targeting of mouse *Nrl* gene by AaCas12b complexed with the optimized sgRNAs. The indel rate is shown under the lanes with mutation.

Figure S5

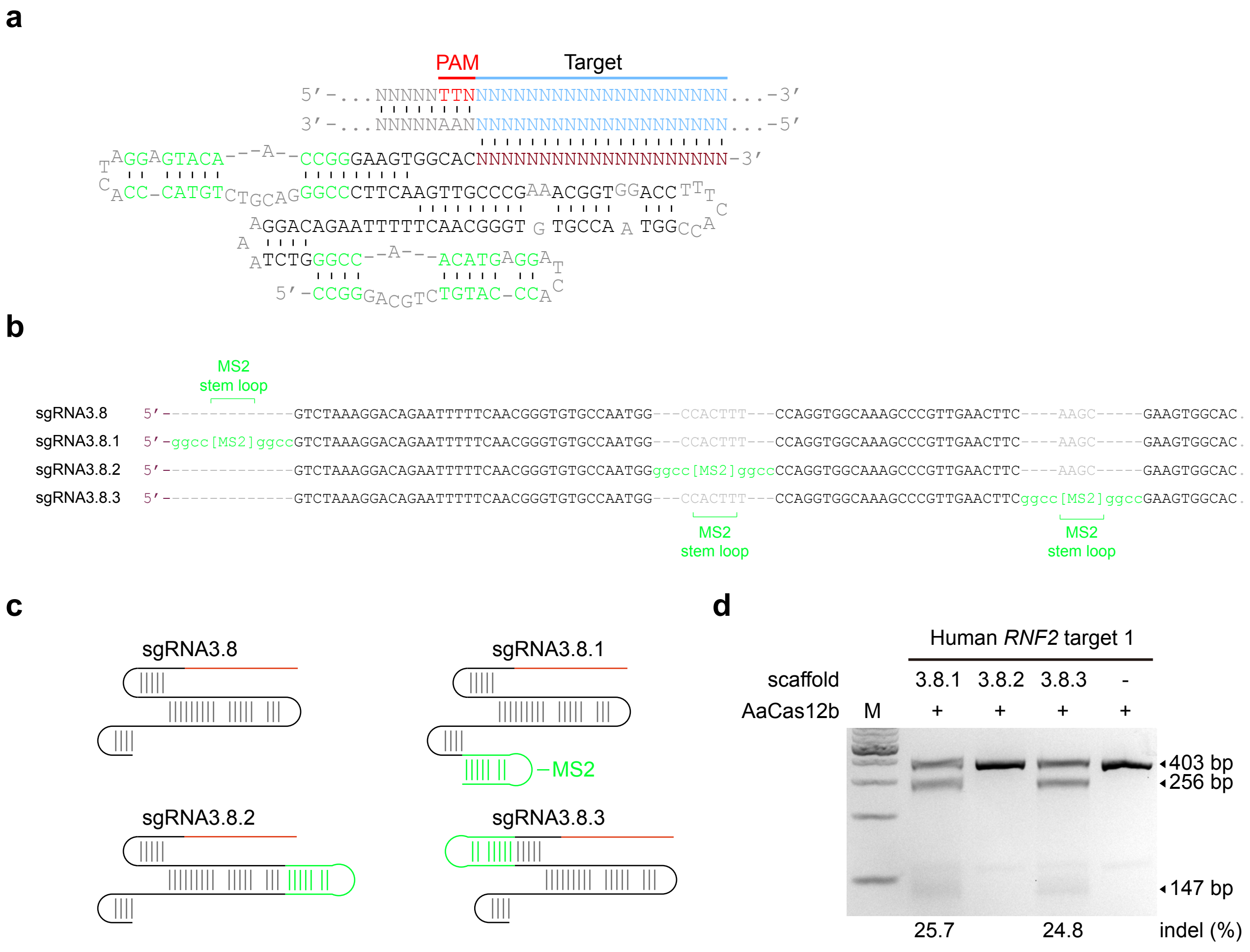

a
PAM
Target
b
MS2 stem loop
sgRNA3.8
sgRNA3.8.1
sgRNA3.8.2
sgRNA3.8.3
MS2 stem loop
MS2 stem loop
c
sgRNA3.8
sgRNA3.8.1
MS2
sgRNA3.8.2
sgRNA3.8.3
d
Human RNF2 target 1
scaffold
3.8.1
3.8.2
3.8.3
-
AaCas12b
M
+
+
+
+
403 bp
256 bp
147 bp
25.7
24.8
indel (%)

Fig. S5 Engineer MS2 hairpin into sgRNA scaffold. a Schematic illustration of the sgRNA3.8 scaffold constructed with the MS2 RNA hairpin. **b** Alignment of the sequences of sgRNA scaffolds engineered with MS2 RNA hairpin. **c** Schematic illustration of AaCas12b sgRNA scaffolds inserted with the MS2 RNA hairpin. **d** Insertion of the MS2 RNA hairpin into AaCas12b sgRNA stem loop 1 and 3, but not the stem loop 2, maintains the cleavage activity of AaCas12b in human cells. The indel rate is shown under the lanes with mutation.

Figure S6

a

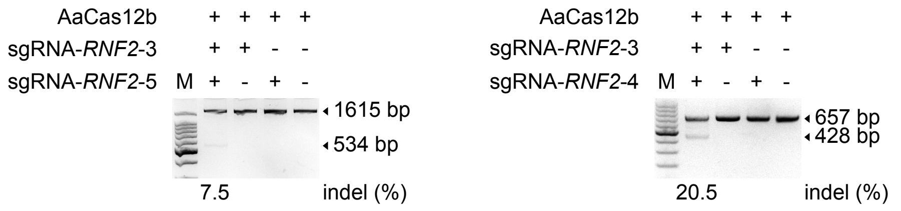

b

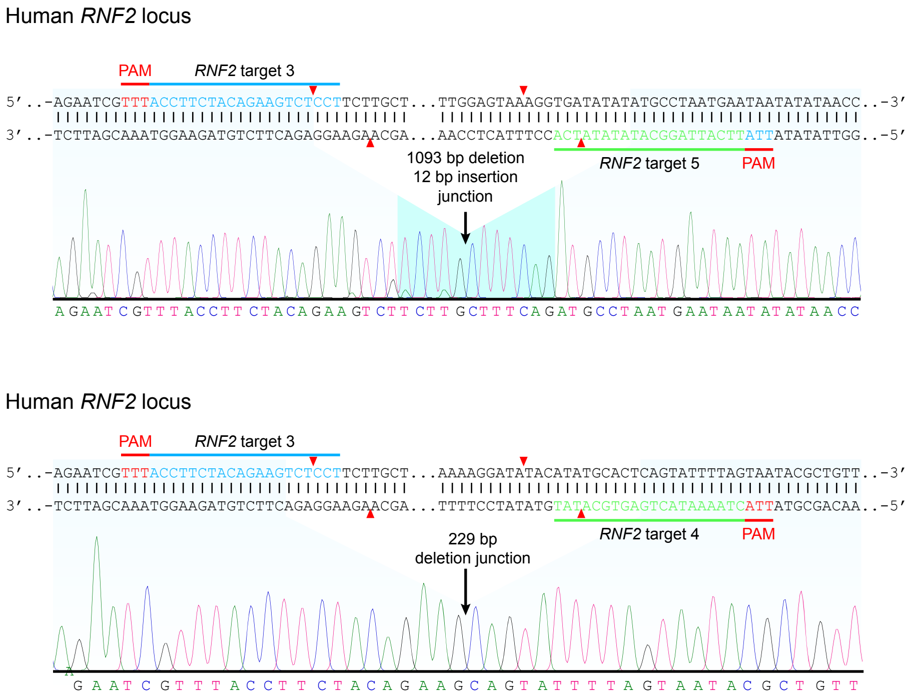

c

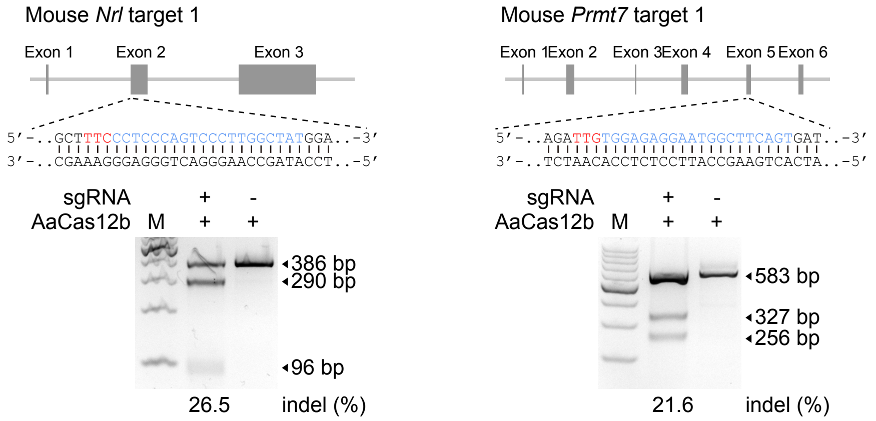

Fig. S6 Multiplex genome editing using AaCas12b combined with sgRNA. **a** AaCas12b-mediated large genomic deletions by simultaneously targeting human *RNF2* target sites 3 and 5 (~1093 bp deletion) (*left*), and human *RNF2* target sites 3 and 4 (~229 bp deletion) (*right*) in 293FT cells. The indel rate is shown under the lanes with mutation. **b** Sanger sequencing results showing the AaCas12b-mediated large genomic deletion by simultaneously targeting human *RNF2* target sites 3 and 5 (~1093 bp deletion) (*left*), and human *RNF2* target sites 3 and 4 (~229 bp deletion) (*right*) in 293FT cells in Fig. S6a. **c** AaCas12b facilitated multiplex genome editing by simultaneously targeting the mouse *Nrl* and *Prmt7* genes using two sgRNAs in the mouse genome. The indel rate is shown under the lanes with mutation.

Figure S7

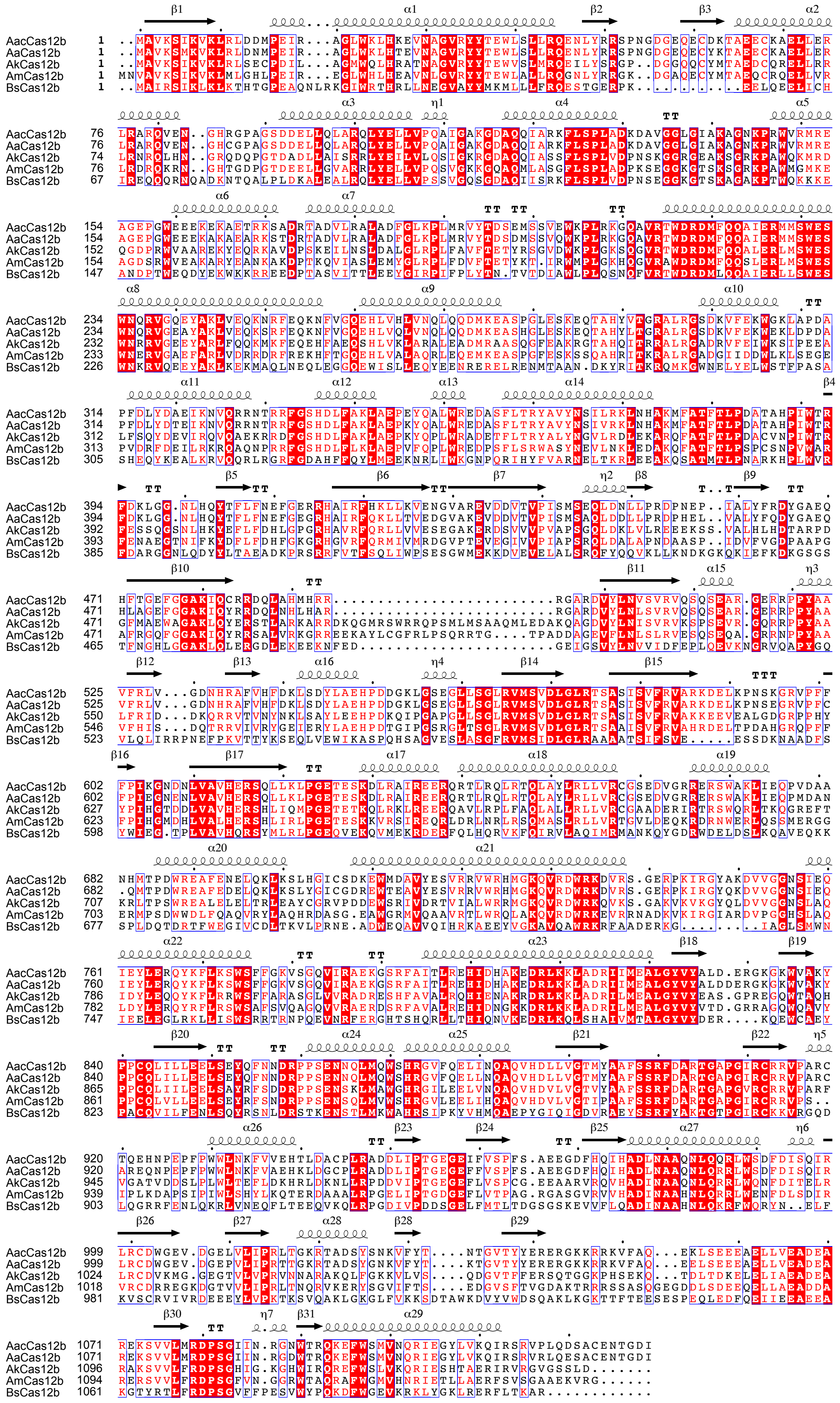

Fig. S7 Protein alignment of Cas12b orthologs. Multiple sequence alignment of amino acid sequences of AaCas12b, AkCas12b, AmCas12b and BsCas12b shows highly conserved residues. Strict identical residues are highlighted with the red background and conserved mutations are highlighted with an outline and red font.

Figure S8

a

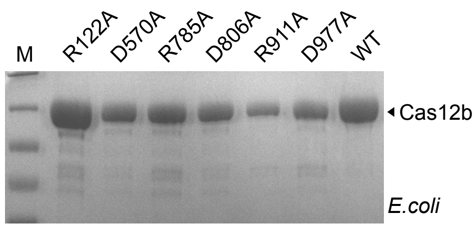

b

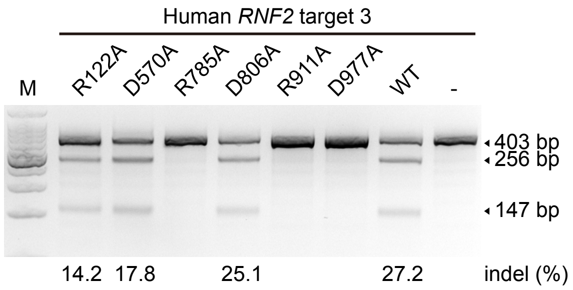

c

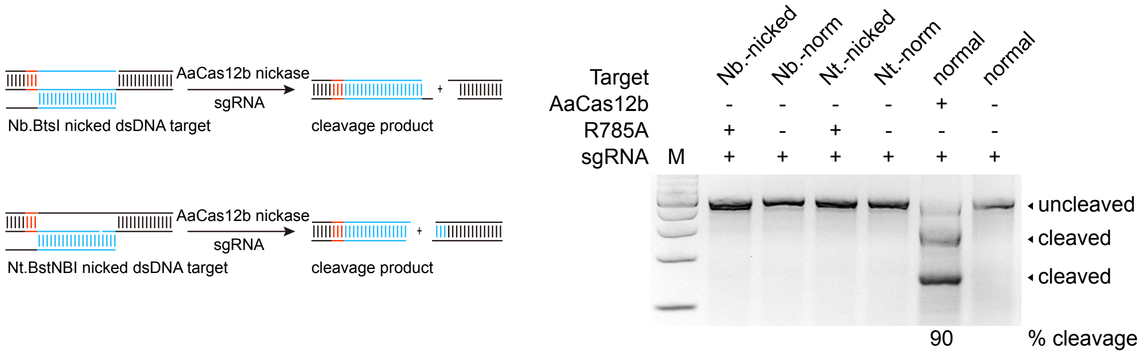

Fig. S8 Catalytic residues required for AaCas12b-mediated DNA cleavage. a Coomassie blue staining of AaCas12b variants (R122A, D570A, R785A, D806A, R911A and D977A) and WT purified from *E. coli*. **b** Effect of mutation of catalytic residues of AaCas12b on DNA targeting in 293FT cells. The indel rate is shown under the lanes with mutation. GFP, an empty backbone vector without Cas12b protein expression. **c** (*Left*) Schematic of *in vitro* cleavage of Nb.BtsI- and Nt.BstNBI-nicked dsDNA fragments using site-directed mutated AaCas12b. (*Right*) *In vitro* nicked dsDNA cleavage analysis of the catalytic residue R785A of AaCas12b. The cleavage rate is shown under the cleaved lanes.

Figure S9

a

Target gene	No. of injected embryos (%)	No. of 2-cell stage embryos (%)	No. of blastocysts (%)	Mutation ratio (%) (no. of mutated/total blastocysts)
Nrl	16	12 (75)	8 (50)	75 (6/8)

b

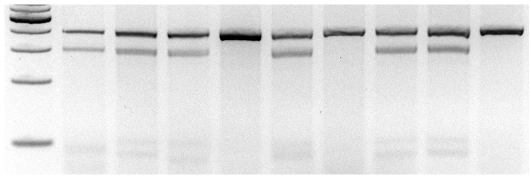

c

Embryo # 1 — Indel (bp)

```
CAGTCCCAGAATGGCTTTCCCTCCCAGTCCCTTGGCTATGGAATATGTTAATGACTTTGATTTGATGAAGTTCGAAAT  WT
CAGTCCCAGAATGGCTTTCCCTCCCAGTCCCTTGGCTATG-------------ACTTTGATTTGATGAAGTTCGAAAT  Δ13
CAGTCCCAGAATGGCTTTCCCTCCCAGTC--------------------attaACTTTGATTTGATGAAGTTCGAAAT  Δ24, +4
CAGTCCCAGAATGGCTTT----------------------------------------GATTTGATGAAGTTCGAAAT  Δ40
CAGTCCCAGAATG-------------------------(Δ 233 bp)------------------------------  Δ233
```

Embryo # 2

```
CAGTCCCAGAATGGCTTTCCCTCCCAGTCCCTTGGCTATGGAATATGTTAATGACTTTGATTTGATGAAGTTCGAAAT  WT
CAGTCCCAGAATGGCTTTCCCTCCCAGTCCCTTGGg---GGAATATGTTAATGACTTTGATTTGATGAAGTTCGAAAT  Δ4, +1
CAGTCCCAGAATGGCTTTCCCTCCCAGTCCC-----TATGGAATATGTTAATGACTTTGATTTGATGAAGTTCGAAAT  Δ5
CAGTCCCAGAATGGCTTTCCCTCCCAGTCCCTTGG-----------GTTAATGACTTTGATTTGATGAAGTTCGAAAT  Δ11
```

Embryo # 3

```
CAGTCCCAGAATGGCTTTCCCTCCCAGTCCCTTGGCTATGGAATATGTTAATGACTTTGATTTGATGAAGTTCGAAAT  WT
CAGTCCCAGAATGGCTTTCCCTCCCAGTCCCTTG--TATGGAATATGTTAATGACTTTGATTTGATGAAGTTCGAAAT  Δ2
CAGTCCCAGAATGGCTTTCCCTCCCAGTCCCTTGGCTA---AATATGTTAATGACTTTGATTTGATGAAGTTCGAAAT  Δ3
CAGTCCCAGAATGGCTTTCCCTCCCAGTCCCTTGtt-----AATATGTTAATGACTTTGATTTGATGAAGTTCGAAAT  Δ7, +2
CAGTCCCAGAATGGCTTTCCCTCCCAGTCCCT--------agATATGTTAATGACTTTGATTTGATGAAGTTCGAAAT  Δ10, +2
```

Embryo # 5

```
CAGTCCCAGAATGGCTTTCCCTCCCAGTCCCTTGGCTATGGAATATGTTAATGACTTTGATTTGATGAAGTTCGAAAT  WT
CAGTCCCAGAATGGCTTTCCCTCCCAGTCCCTT--------AATATGTTAATGACTTTGATTTGATGAAGTTCGAAAT  Δ8
CAGTCCCAGAATGGCTTTCCCTCCCAGTCCCT----------ATATGTTAATGACTTTGATTTGATGAAGTTCGAAAT  Δ10
CAGTCtctg-----------------------------(Δ 95 bp)-------------------------------  Δ95
CAGTCCCAGAATGGCTTTCCCTCCCAGT----------------(Δ 132 bp)------------------------  Δ132
```

Embryo # 7

```
CAGTCCCAGAATGGCTTTCCCTCCCAGTCCCTTGGCTATGGAATATGTTAATGACTTTGATTTGATGAAGTTCGAAAT  WT
CAGTCCCAGAATGGCTTTCCCTCCCAGTCCCTT-----TGGAATATGTTAATGACTTTGATTTGATGAAGTTCGAAAT  Δ5
CAGTCCCAGAATGGCTTTCCCTCCCAGTCCCTT------GGAATATGTTAATGACTTTGATTTGATGAAGTTCGAAAT  Δ6
CAGTCCCAGAATGGCTTTCCCTCCCAGTCCCTTG--------cTATGTTAATGACTTTGATTTGATGAAGTTCGAAAT  Δ9, +1
CAGTCCCAGAATGGCTTTCCCTCCCAGTCCCT---------AATATGTTAATGACTTTGATTTGATGAAGTTCGAAAT  Δ9
CAGTCCCAGAATGGCTTTCCCTCCCAGTCCCTT-----------ATGTTAATGACTTTGATTTGATGAAGTTCGAAAT  Δ11
```

Embryo # 8

```
CAGTCCCAGAATGGCTTTCCCTCCCAGTCCCTTGGCTATGGAATATGTTAATGACTTTGATTTGATGAAGTTCGAAAT  WT
CAGTCCCAGAATGGCTTTCCCTCCCAGTCCCTTG-CTATGGAATATGTTAATGACTTTGATTTGATGAAGTTCGAAAT  Δ1
CAGTCCCAGAATGGCTTTCCCTCCCAGTCCC------ATGGAATATGTTAATGACTTTGATTTGATGAAGTTCGAAAT  Δ6
CAGTCCCAGAATGGCTTTCCCTCCCAGTCCCT-----------TATGTTAATGACTTTGATTTGATGAAGTTCGAAAT  Δ11
CAGTCCCAGAATGGCT---------------------ATGGAATATGTTAATGACTTTGATTTGATGAAGTTCGAAAT  Δ21
```

Figure S9. Mutated embryos generated by AaCas12b RNP microinjection. a Summary of mutated embryos generated by preassembled AaCas12b RNP microinjection. **b** T7EI-based genotyping analysis of mouse embryos with AaCas12b RNP injected. The red font highlighted numbers denote induced mutants. **c** Representative indels in mutated embryos in Fig. S9b. Blue dashes, deleted bases; purple lowercases, insertions or mutations; red uppercases, PAM.

Figure S10

a

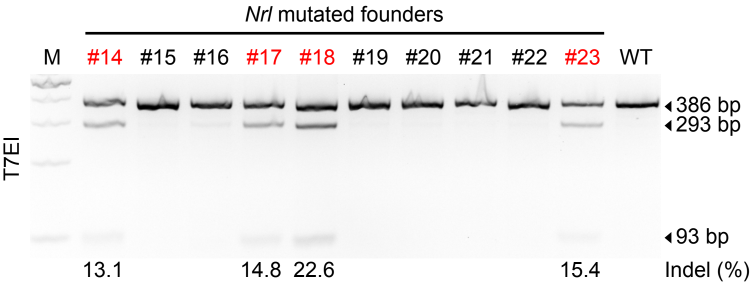

b

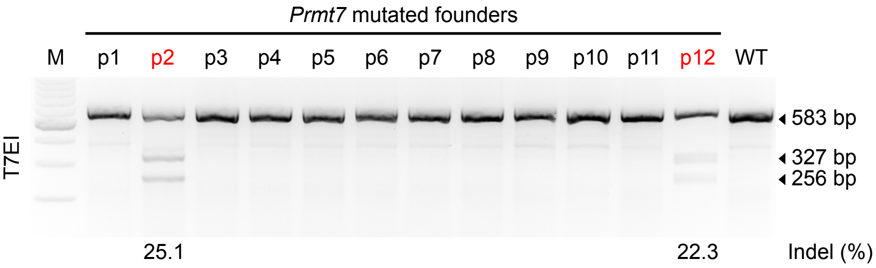

c

Mouse #1	Indel (bp)	Frequency (%)
CAGTCCCAGAATGGCTTTCCCTCCCAGTCCCTTGGCTATGGAATATGTTAATGACTTTGATTTGATGAAGTTCGAAAT	WT	
CAGTCCCAGAATGGCTTTCCCTCCCAGTC-------------------TAATGACTTTGATTTGATGAAGTTCGAAAT	Δ19	4.8 (1/21)
CAGTCCCAGAATGGCTTTCCC--------------------------aaAATGACTTTGATTTGATGAAGTTCGAAAT	Δ28, +2	19.0 (4/23)
**Mouse #4**		
CAGTCCCAGAATGGCTTTCCCTCCCAGTCCCTTGGCTATGGAATATGTTAATGACTTTGATTTGATGAAGTTCGAAAT	WT	
CAGTCCCAGAATGGCTTTCCCTCCCAGTCCCTTGG-----GAATATGTTAATGACTTTGATTTGATGAAGTTCGAAAT	Δ5	28.6 (6/21)
**Mouse #5**		
CAGTCCCAGAATGGCTTTCCCTCCCAGTCCCTTGGCTATGGAATATGTTAATGACTTTGATTTGATGAAGTTCGAAAT		
CAGTCCCAGAATGGCTTTCCCTCCCAGTCC--------------ATGTTAATGACTTTGATTTGATGAAGTTCGAAAT	Δ14	17.2 (5/19)
CAGTCCCAGAATGGCTTTCCCTCCCAGTCCC---------------GTTAATGACTTTGATTTGATGAAGTTCGAAAT	Δ15	10.3 (3/19)
**Mouse #8**		
CAGTCCCAGAATGGCTTTCCCTCCCAGTCCCTTGGCTATGGAATATGTTAATGACTTTGATTTGATGAAGTTCGAAAT	WT	
CAGTCCCAGAATGGCTTTCCCTCCCAGTC---------------ATGTTAATGACTTTGATTTGATGAAGTTCGAAAT	Δ15	26.0 (7/26)
**Mouse #9**		
CAGTCCCAGAATGGCTTTCCCTCCCAGTCCCTTGGCTATGGAATATGTTAATGACTTTGATTTGATGAAGTTCGAAAT	WT	
CAGTCCCAGAATGGCTTTCCCTCCCAGTCCCT-------------TGTTAATGACTTTGATTTGATGAAGTTCGAAAT	Δ13	28 (7/25)
**Mouse #10**		
CAGTCCCAGAATGGCTTTCCCTCCCAGTCCCTTGGCTATGGAATATGTTAATGACTTTGATTTGATGAAGTTCGAAAT	WT	
CAGTCCCAGAATGGCTTTCCCTCCCAGTCCCTT-------GAATATGTTAATGACTTTGATTTGATGAAGTTCGAAAT	Δ7	10.3 (3/29)
CAGTCCCAGAATGGCTTTCCCTCCCAGTCC------------ATATGTTAATGACTTTGATTTGATGAAGTTCGAAAT	Δ12	6.7 (2/29)
CAGTCCCAGAATGGCTTTCCCTCCCAGTCCC------------TATGTTAATGACTTTGATTTGATGAAGTTCGAAAT	Δ12	17.3 (5/29)
**Mouse #14**	**Indel (bp)**	**Frequency (%)**
CAGTCCCAGAATGGCTTTCCCTCCCAGTCCCTTGGCTATGGAATATGTTAATGACTTTGATTTGATGAAGTTCGAAAT	WT	
CAGTCCCAGAATGGCTTTCCCTCCCAGTCCCTTGGCTATt---TATGTTAATGACTTTGATTTGATGAAGTTCGAAAT	Δ4, +1	15 (3/20)
**Mouse #17**		
CAGTCCCAGAATGGCTTTCCCTCCCAGTCCCTTGGCTATGGAATATGTTAATGACTTTGATTTGATGAAGTTCGAAAT	WT	
CAGTCCCAGAATGGCTTTCCCTCCCAGTCCCTTGtt-----AATATGTTAATGACTTTGATTTGATGAAGTTCGAAAT	Δ7, +2	21.7 (5/23)
**Mouse #18**		
CAGTCCCAGAATGGCTTTCCCTCCCAGTCCCTTGGCTATGGAATATGTTAATGACTTTGATTTGATGAAGTTCGAAAT	WT	
CAGTCCCAGAATGGCTTTCCCTCCCAGTCCCTTG--------ATATGTTAATGACTTTGATTTGATGAAGTTCGAAAT	Δ8	54.5 (12/22)
**Mouse #23**		
CAGTCCCAGAATGGCTTTCCCTCCCAGTCCCTTGGCTATGGAATATGTTAATGACTTTGATTTGATGAAGTTCGAAAT	WT	
CAGTCCCAGAATGGCTTTCCCTCCCAGTCCC-------------ATGTTAATGACTTTGATTTGATGAAGTTCGAAAT	Δ13	50 (12/24)

d

Mouse p4	Indel (bp)	Frequency (%)
GAGGCTGCTGTGAAGATTGTGGAGAGGAATGGCTTCAGTGATAAGATTAAAGTCATTAACAAGCACTCCACTGAGGTG	WT	
GAGGCTGCTGTGAAGATTGTGGAGAGGA-------------gggaaaatgagcCATTAACAAGCACTCCACTGAGGTG	Δ25, +12	37.8 (14/37)
**Mouse p12**		
GAGGCTGCTGTGAAGATTGTGGAGAGGAATGGCTTCAGTGATAAGATTAAAGTCATTAACAAGCACTCCACTGAGGTG	WT	
GAGGCTGCTGTGAAGATTGTGGAGAGGAATGGCT-----GATAAGATTAAAGTCATTAACAAGCACTCCACTGAGGTG	Δ5	66.7 (16/24)

Fig. S10 Mutated mice generated by AaCas12b RNP microinjection. a *Nrl* mutated founder mice generated by microinjection of AaCas12b RNPs. The numbers in red denote newborn mice with induced indel mutations. The indel rate is shown under the lanes with mutation. **b** *Prmt7* mutated founder mice generated by microinjection of AaCas12b RNPs. The numbers in red denote newborn mice with induced indel mutations. The indel rate is shown under the lanes with mutation. **c** Sanger sequencing of targeted *Nrl* alleles in mutated mice induced by injection of AaCas12b RNPs in Fig. 5d and S10a. Blue dashes, deleted bases; purple lowercases, insertions or mutations; red uppercases, PAM. Indel frequencies are indicated. **d** Sanger sequencing of targeted *Prmt7* alleles in mutated mice induced by injection of AaCas12b RNPs in Fig. S10b. Blue dashes, deleted bases; purple lowercases, insertions or mutations; red uppercases, PAM. Indel frequencies are indicated.

Figure S11

a

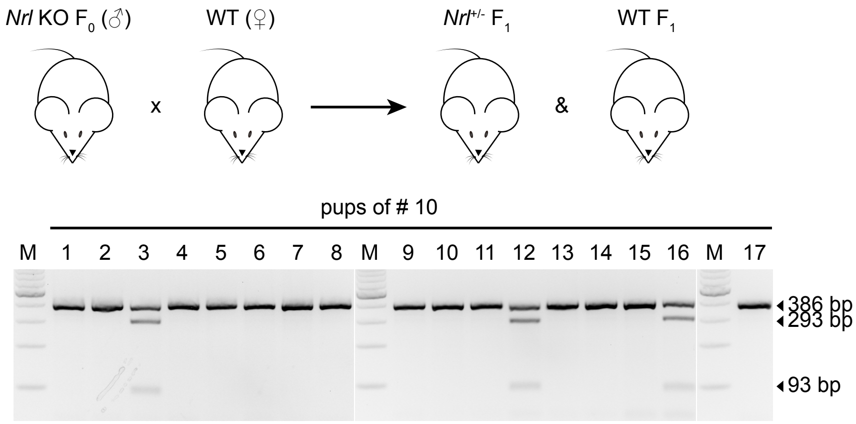


```
WT  CCAGAATGGCTTTCCCTCCCAGTCCCTTGGCTATGGAATATGTTAATGACTTTGATTTGATGAAGTTCG
3   CCAGAATGGCTTTCCCTCCCAGTCCCTT-------GAATATGTTAATGACTTTGATTTGATGAAGTTCG  Δ7
12  CCAGAATGGCTTTCCCTCCCAGTCCCTT-------GAATATGTTAATGACTTTGATTTGATGAAGTTCG  Δ7
16  CCAGAATGGCTTTCCCTCCCAGTCCCTT-------GAATATGTTAATGACTTTGATTTGATGAAGTTCG  Δ7
```

b

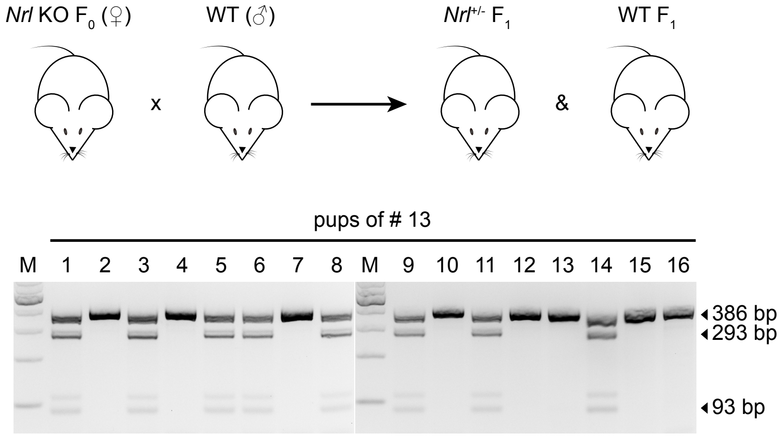


```
WT  CCAGAATGGCTTTCCCTCCCAGTCCCTTGGCTATGGAATATGTTAATGACTTTGATTTGATGAAGTTCG
1   CCAGAATGGCTTTCCCTCCCAGTCCCTT------------------------TGATTTGATGAAGTTCG  Δ24
3   CCAGAATGGCTTTCCCTCCCAGTCCCTT------------------------TGATTTGATGAAGTTCG  Δ24
5   CCAGAATGGCTTTCCCTCCCAGTCCCTT------------------------TGATTTGATGAAGTTCG  Δ24
6   CCAGAATGGCTTTCCCTCCCAGTCCCTT------------------------TGATTTGATGAAGTTCG  Δ24
8   CCAGAATGGCTTTCCCTCCCAGTCCCTT------------------------TGATTTGATGAAGTTCG  Δ24
9   CCAGAATGGCTTTCCCTCCCAGTCCCTT------------------------TGATTTGATGAAGTTCG  Δ24
11  CCAGAATGGCTTTCCCTCCCAGTCCCTT------------------------TGATTTGATGAAGTTCG  Δ24
14  CCAGAATGGCTTTCCCTCCCAGTCCCTT------------------------TGATTTGATGAAGTTCG  Δ24
```

Fig. S11 Successful germline transmission of mutated founders. a *Nrl*-mutant male founder #10 crossed with WT female mouse (*top*) and the genotypes of the pups were determined by T7EI assay (*middle*). Sanger sequencing reads represent genotypes of mutants (*bottom*). **b** *Nrl*-mutant female founder #13 crossed with WT male mouse (*top*) and the genotypes of the pups were determined by T7EI assay (*middle*). Sanger sequencing reads represent genotypes of mutants (*bottom*).

Figure S12

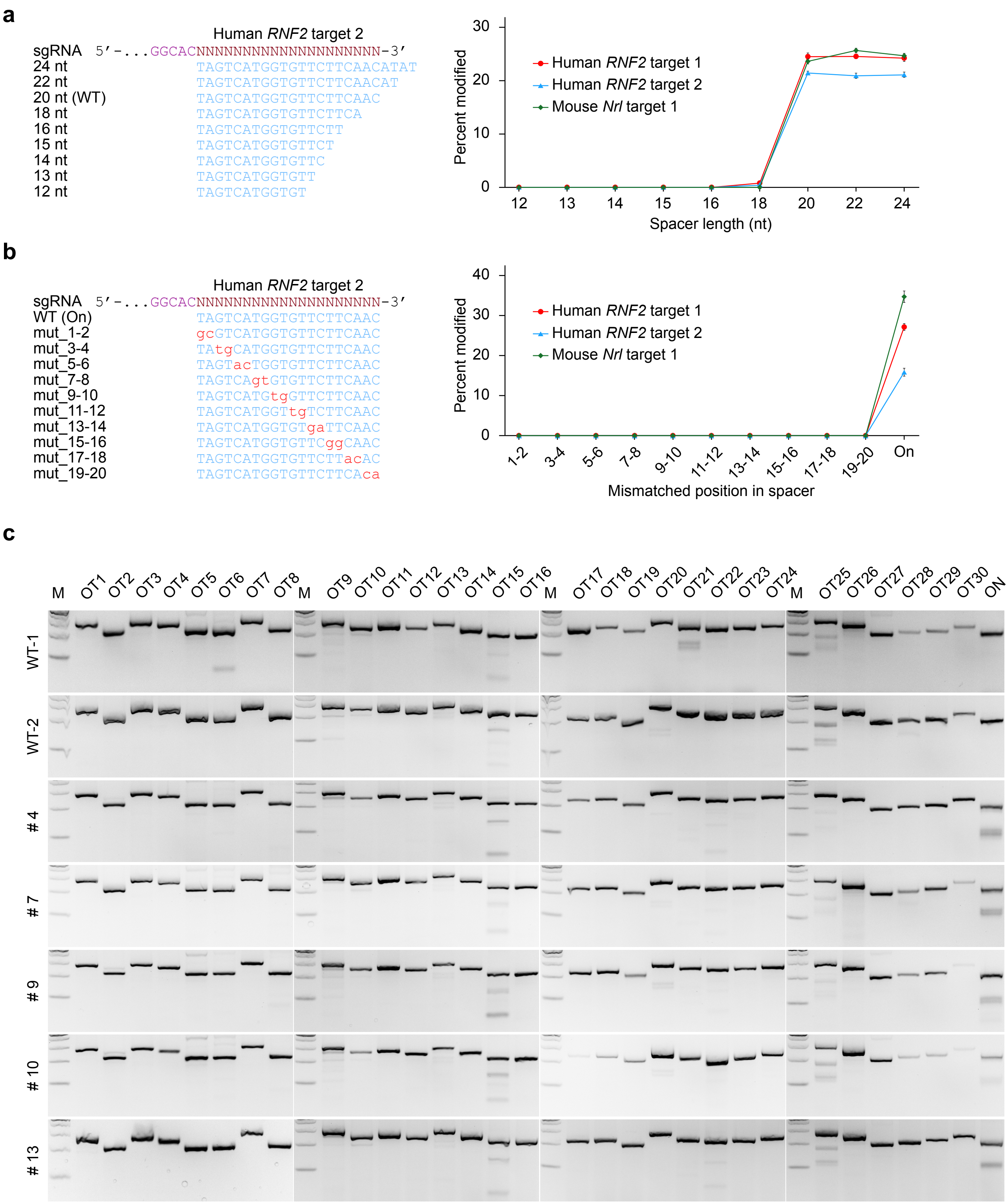

a
Human RNF2 target 2
sgRNA 5′-...GGCACNNNNNNNNNNNNNNNNNNNNN-3′
24 nt TAGTCATGGTGTTCTTCAACATAT
22 nt TAGTCATGGTGTTCTTCAACAT
20 nt (WT) TAGTCATGGTGTTCTTCAAC
18 nt TAGTCATGGTGTTCTTCA
16 nt TAGTCATGGTGTTCTT
15 nt TAGTCATGGTGTTCT
14 nt TAGTCATGGTGTTC
13 nt TAGTCATGGTGTT
12 nt TAGTCATGGTGT
Percent modified
Human RNF2 target 1
Human RNF2 target 2
Mouse Nrl target 1
Spacer length (nt)
b
Human RNF2 target 2
sgRNA 5′-...GGCACNNNNNNNNNNNNNNNNNNNNN-3′
WT (On) TAGTCATGGTGTTCTTCAAC
mut_1-2 gcGTCATGGTGTTCTTCAAC
mut_3-4 TAtgCATGGTGTTCTTCAAC
mut_5-6 TAGTacTGGTGTTCTTCAAC
mut_7-8 TAGTCAgtGTGTTCTTCAAC
mut_9-10 TAGTCATGtgGTTCTTCAAC
mut_11-12 TAGTCATGGTtgTCTTCAAC
mut_13-14 TAGTCATGGTGTgaTTCAAC
mut_15-16 TAGTCATGGTGTTCggCAAC
mut_17-18 TAGTCATGGTGTTCTTacAC
mut_19-20 TAGTCATGGTGTTCTTCAca
Percent modified
Human RNF2 target 1
Human RNF2 target 2
Mouse Nrl target 1
Mismatched position in spacer
c
M OT1 OT2 OT3 OT4 OT5 OT6 OT7 OT8 M OT9 OT10 OT11 OT12 OT13 OT14 OT15 OT16 M OT17 OT18 OT19 OT20 OT21 OT22 OT23 OT24 M OT25 OT26 OT27 OT28 OT29 OT30 ON
WT-1
WT-2
4
7
9
10
13

d

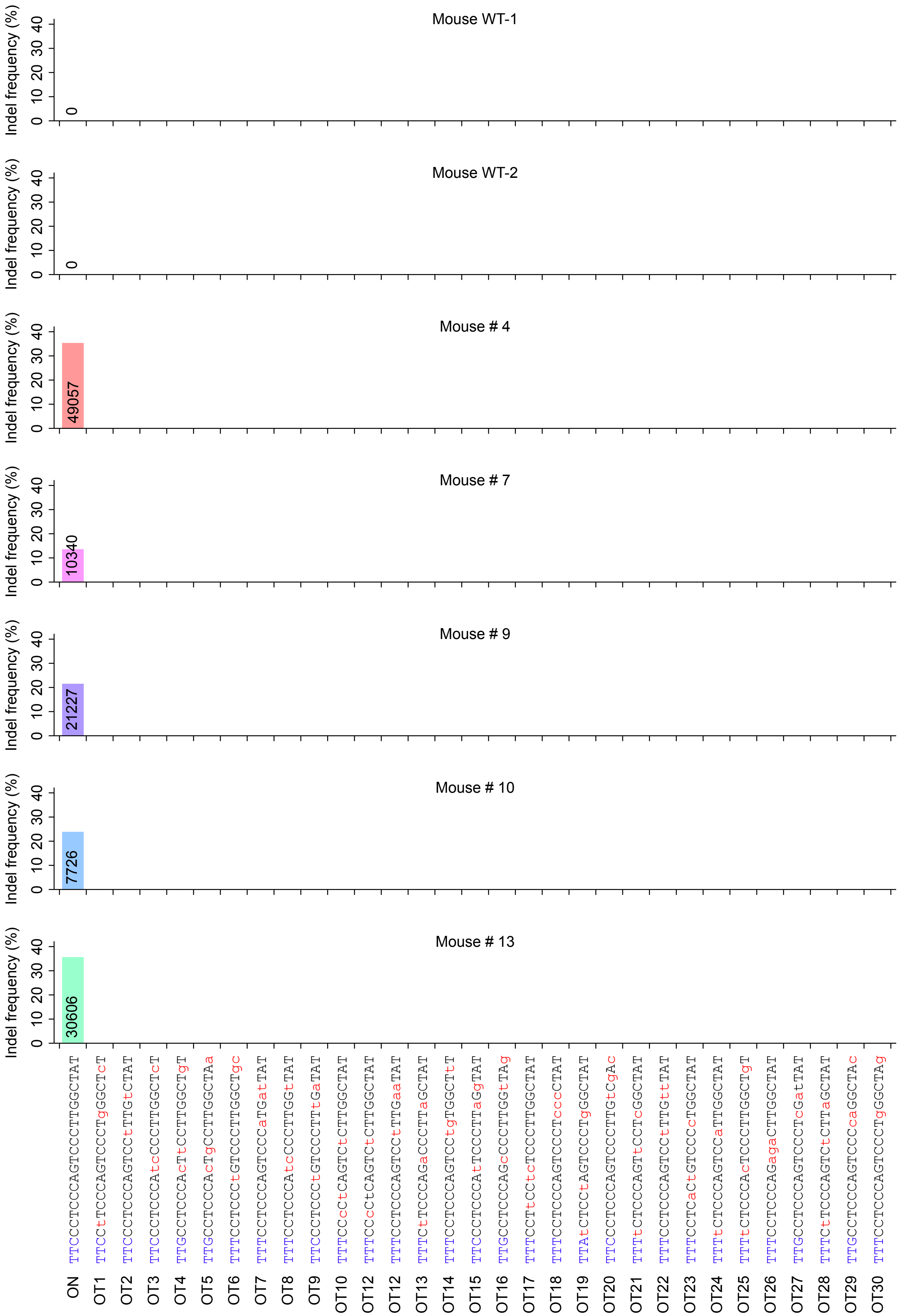

Mouse WT-1
Mouse WT-2
Mouse # 4
Mouse # 7
Mouse # 9
Mouse # 10
Mouse # 13
Indel frequency (%)
0
49057
10340
21227
7726
30606
ON
OT1
OT2
OT3
OT4
OT5
OT6
OT7
OT8
OT9
OT10
OT12
OT12
OT13
OT14
OT15
OT16
OT17
OT18
OT19
OT20
OT21
OT22
OT23
OT24
OT25
OT26
OT27
OT28
OT29
OT30

Fig. S12 Analysis of off-target effects in mammalian genomes. a Effects of spacer length on AaCas12b/sgRNA cleavage activity in human and mouse cells. Error bars indicate standard errors of the mean (s.e.m.), n = 3. **b** Analysis of cleavage specificity of AaCas12b/sgRNA in human and mouse cells using sgRNAs carrying double base-pair mismatches in the guide sequence. Error bars indicate s.e.m., n = 3. **c** T7EI analysis of the 30 potential off-targets related to *Nrl* target in the mouse genome. Two WT mice and five indicated founder mice were applied for detection and no detectable off-target effects existed by T7EI assay. **d** Pooled PCR products containing the 30 off-target sites per mice were subjected to deep sequencing. Sequences containing insertions and deletions around the cleavage site were considered to be AaCas12b-induced mutations.

Figure S13

a

Sample-specific variant observed in WGS data

Chromosome		2
Position		151978463
Reference		ATTCTCACCCAGACCCTTTGCT-AGTCATC
Alternate		ATTCTCACCCAGACCCTTTGCTcAGTCATC
Frequency	#4 (♀)	41.0% (16 / 39)
	# 7 (♀)	43.2% (16 / 37)
	# 9 (♂)	40% (14 / 35)
	# 10 (♂)	100% (19 / 19)

b

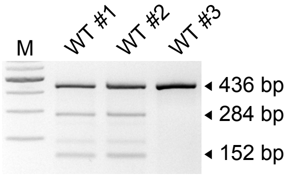


```
Reference TGTGTAATTCTCACCCAGACCCTTTGCT-AGTCATCTATGTT
WT #1     TGTGTAATTCTCACCCAGACCCTTTGCTcAGTCATCTATGTT
WT #2     TGTGTAATTCTCACCCAGACCCTTTGCTcAGTCATCTATGTT
WT #3     TGTGTAATTCTCACCCAGACCCTTTGCT-AGTCATCTATGTT
```

Fig. S13 Sample-specific variant is not induced by off-target effects. a Sample-specific variant observed in WGS data. All the four *Nrl*-mutated founders contain the same variant and the indel frequencies are shown. **b** T7EI assay (*top*) and Sanger sequencing (*bottom*) showing the sample-specific variant in Fig. 6e also observed in wild-type siblings (WT #1 and #2).

Figure S14

a

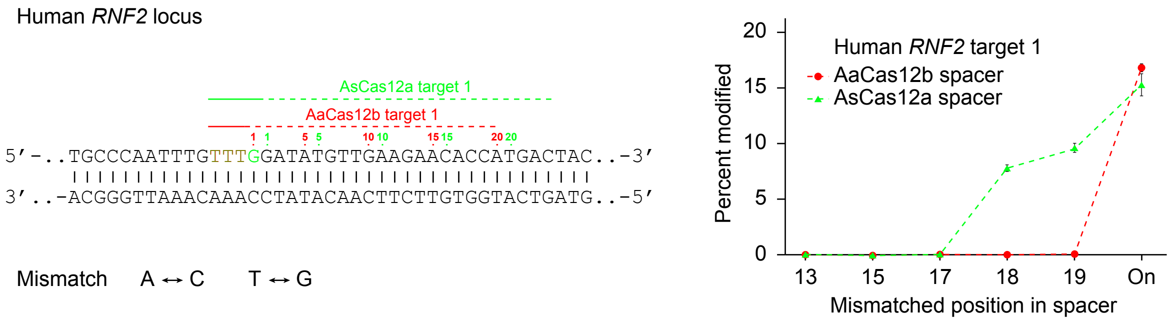

b

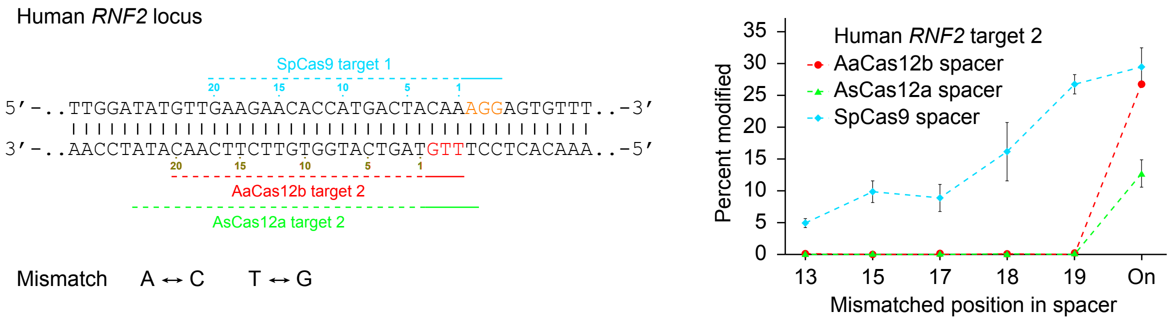

Fig. S14 Comparison of off-target effects of AaCas12b, AsCas12a and SpCas9 in mammalian cells. a-b (*Left*) Schematic showing the targeting sequences of AaCas12b, AsCas12a and SpCas9 in human *RNF2* locus. (*Right*) Activities of AaCas12b, AsCas12a and SpCas9 targeted to the human *RNF2* locus using respective guide RNAs with single mismatches in human 293FT cells. Mutation frequencies were assessed by T7EI assay. Error bars indicate standard errors of the mean (s.e.m.), n = 3.

Figure S15

a

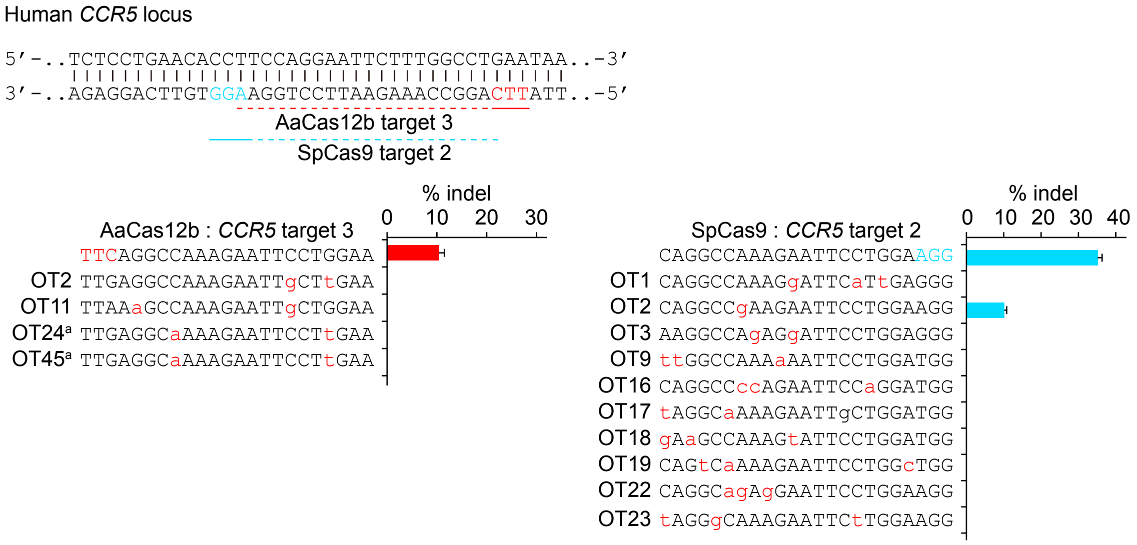

b

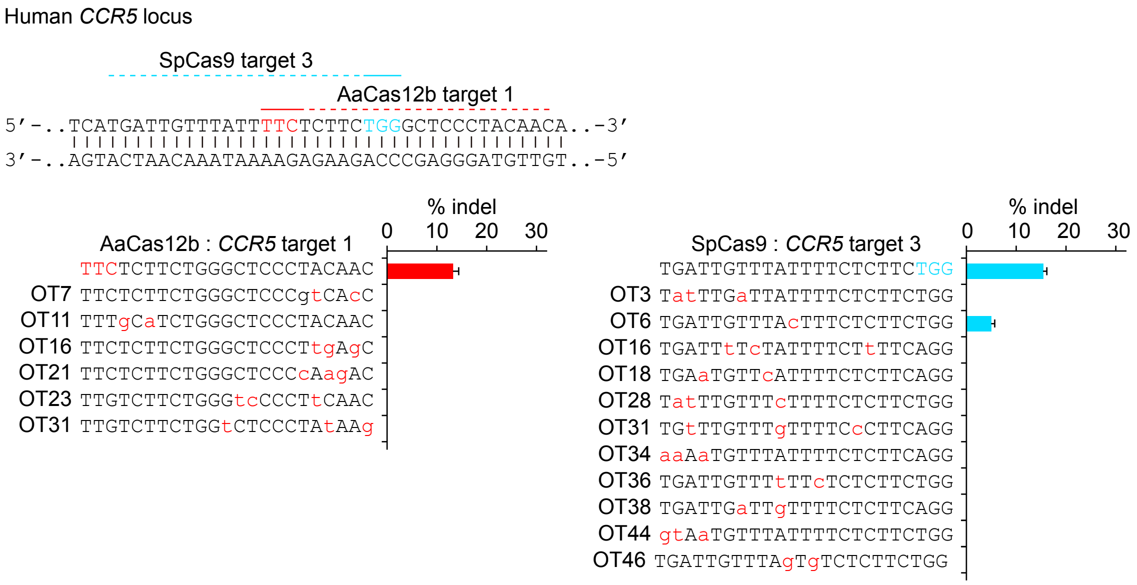

Fig. S15 AaCas12b generates minimal genome-wide off-target effects compared to SpCas9. a-b (*Upper*) Schematic showing the targeting sequences of AaCas12b and SpCas9 in the human *CCR5* locus. (*Lower*) Frequencies of induced indels at on- and off-target sites by AaCas12b and SpCas9 in human 293FT cells. Mutation frequencies were assessed by T7EI assay. Error bars indicate standard errors of the mean (s.e.m.), n = 2.

Table S1. Oligonucleotides (oligos) used for Cas12b coding gene and guide RNA synthesis.
See separate Excel file

Table S2. Target sequences bearing various 5' PAM sequences used for *in vitro* DNA cleavage assay. Target sequences used for in vitro DNA cleavage analysis of PAM sequences were commercially synthesized with EcoRI 5' and SphI 3' overhangs highlighted with yellow and green backgrounds, respectively. Annealed oligos were constructed into EcoRI and SphI double-digested p11-LacY-wtx1 vector.
See separate Excel file

Table S3. Protospacer sequences of mammalian genomic targets. Protospacer targets designed based on CRISPR-Cas12b loci with their requisite PAMs against different genes in human and mouse genomes. The PAMs are highlighted in blue uppercase and mismatches in red lowercase.
See separate Excel file

Table S4. Frequency of AaCas12b-induced indel mutations at potential off-target sites in mammalian genomes. Sites in the human and mouse genomes bearing 1 to 3 mismatches from the *CCR5*, *RNF2* and *Nrl* on-target sites for AaCas12b were detected by targeted deep sequencing. PAMs were highlighted in red, and mismatches were within lowercase.
See separate Excel file

Table S5. Frequency of AaCas12b- and SpCas9-induced indel mutations at potential off-target sites in human 293FT cells. Sites in the human genome bearing 1 to 3 mismatches from the *CCR5* and *RNF2* on-target sites for AaCas12b and SpCas9 were detected by T7EI assay. PAMs were highlighted in red, and mismatches were within lowercase.
See separate Excel file

Table S6. List of primer sequences used in this study.
See separate Excel file

Supplementary Sequences

Accession information for the four Cas12b orthologs and vectors used in this study.

Cas12b coding sequence from *Alicyclobacillus acidiphilus* NBRC 100859 (GeneBank ID: NZ_BCQI01000053.1)

ATGGCCGTTAAATCCATGAAAGTGAAACTTCGCCTCGATAATATGCCGGAGATTCGGGCTGG
TTTATGGAAACTCCATACGGAGGTCAACGCGGGGGTTCGATATTACACGGAATGGCTGAGTC
TTCTGCGTCAAGAGAATTTGTATCGAAGAAGTCCGAATGGGGACGGAGAGCAAGAATGTTAT
AAGACTGCAGAAGAATGCAAAGCCGAATTGTTGGAGCGGCTGCGCGCGCGTCAAGTGGAGAA
TGGACACTGTGGTCCGGCGGGATCGGACGATGAATTGCTGCAGTTGGCTCGTCAACTTTATG
AACTGTTGGTTCCGCAGGCGATAGGTGCGAAAGGCGATGCGCAGCAAATTGCGCGCAAGTTT
TTGAGCCCCTTAGCCGACAAGGATGCAGTGGGTGGGCTTGGAATCGCGAAGGCGGGGAACAA
ACCGCGGTGGGTTCGCATGCGCGAAGCGGGAGAACCTGGCTGGGAAGAGGAGAAGGCGAAGG
CTGAGGCGAGGAAATCTACGGATCGAACTGCGGATGTTTTGCGCGCGCTCGCGGATTTTGGG
TTAAAGCCACTGATGCGCGTGTACACCGATTCTGACATGTCATCTGTTCAGTGGAAACCGCT
TCGGAAGGGCCAAGCGGTTCGGACGTGGGACAGGGATATGTTCCAACAGGCCATCGAGCGGA
TGATGTCGTGGGAGTCGTGGAATCAGCGCGTTGGCGAAGCGTACGCGAAACTGGTAGAGCAA
AAAAGTCGATTTGAGCAGAAGAACTTCGTCGGCCAGGAACATTTGGTTCAACTCGTCAATCA
GTTGCAACAAGATATGAAAGAAGCATCGCACGGGCTCGAATCGAAAGAGCAAACCGCACATT
ATCTGACGGGACGGGCATTGCGCGGATCGGACAAAGTGTTTGAGAAGTGGGAGAAACTCGAC
CCTGATGCGCCATTCGATTTGTACGACACCGAAATCAAGAACGTGCAGAGACGTAACACGAG
GCGATTCGGCTCACACGACTTGTTCGCGAAATTGGCGGAACCGAAGTATCAGGCCCTGTGGC
GCGAAGATGCTTCGTTTCTCACGCGTTACGCGGTGTACAACAGCATCGTTCGCAAACTGAAT
CACGCCAAAATGTTCGCGACGTTTACTTTACCGGATGCAACTGCGCATCCGATTTGGACTCG
CTTTGATAAATTGGGCGGGAATTTGCACCAGTACACCTTTTTGTTCAACGAATTCGGAGAAG
GCAGGCACGCGATTCGTTTTCAAAAGCTGTTGACCGTCGAAGATGGTGTCGCAAAAGAAGTT
GATGATGTAACGGTGCCCATTTCCATGTCAGCGCAATTGGATGATCTGCTGCCAAGAGATCC
CCATGAACTGGTTGCACTATATTTTCAAGATTATGGAGCCGAACAGCATTTGGCGGGTGAAT
TCGGTGGCGCGAAGATTCAGTACCGTCGGGATCAACTAAATCATTTGCACGCACGCAGAGGG
GCGAGGGATGTTTATCTCAATCTCAGCGTACGTGTGCAGAGCCAGTCTGAGGCACGGGGAGA
ACGCCGCCCGCCGTATGCCGCAGTATTCCGCCTGGTCGGGGACAACCATCGTGCGTTTGTCC
ATTTTGATAAATTATCGGATTATCTTGCGGAACATCCGGATGATGGGAAGCTTGGATCGGAG
GGGCTGCTTTCCGGGCTACGGGTGATGAGTGTCGATCTCGGCCTTCGCACATCGGCATCGAT
TTCCGTTTTTCGCGTTGCCCGGAAGGACGAGTTGAAGCCGAACTCGGAAGGGCGTGTCCCAT
TCTGTTTTCCGATTGAAGGGAATGAAAATCTCGTCGCGGTTCATGAACGATCTCAACTTTTG
AAGCTGCCTGGCGAAACAGAGTCAAAGGACCTGCGGGCTATCCGAGAAGAGCGCCAACGGAC
CCTGCGGCAGCTGCGGACGCAACTGGCGTATTTGCGGCTGCTCGTGCGGTGTGGGTCGGAAG
ATGTGGGACGGCGTGAACGGAGTTGGGCAAAGCTTATTGAGCAGCCCATGGATGCCAATCAG
ATGACACCGGATTGGCGCGAAGCCTTTGAAGACGAACTTCAGAAGCTTAAGTCACTCTATGG
TATCTGTGGCGACAGGGAATGGACGGAGGCTGTCTACGAGAGCGTTCGCCGCGTGTGGCGCC
ATATGGGCAAACAGGTTCGCGATTGGCGAAAGGACGTACGGAGTGGAGAGCGGCCGAAGATT
CGCGGCTATCAAAAAGATGTGGTCGGCGGAAATTCGATTGAGCAAATTGAGTATCTTGAACG

GCAGTACAAGTTTCTCAAGAGTTGGAGCTTTTTTGGCAAGGTATCGGGACAAGTGATTCGTG
CGGAGAAGGGATCCCGATTTGCGATCACGCTGCGTGAACACATTGATCACGCGAAGGAAGAC
CGGCTGAAGAAATTGGCGGATCGCATCATTATGGAGGCGCTCGGTTATGTGTACGCGTTGGA
TGATGAGCGCGGCAAAGGAAAGTGGGTTGCGAAGTATCCGCCGTGCCAGCTCATCCTGCTGG
AGGAATTGAGCGAGTACCAGTTCAATAACGACAGGCCTCCGAGTGAAAACAATCAGTTGATG
CAATGGAGCCATCGCGGCGTGTTCCAGGAGTTGTTGAATCAGGCCCAAGTCCACGATTTACT
CGTTGGGACGATGTATGCAGCGTTCTCGTCGCGATTCGACGCGCGAACCGGGGCACCGGGTA
TCCGCTGTCGCAGGGTACCGGCGCGTTGCGCTCGGGAGCAGAATCCAGAACCATTTCCTTGG
TGGCTGAACAAGTTTGTGGCGGAACACAAGTTGGATGGTTGTCCCTTACGGGCAGACGACCT
CATCCCCACGGGTGAAGGAGAGTTTTTTGTCTCGCCGTTCAGTGCGGAGGAAGGGGACTTTC
ATCAGATTCATGCCGACCTGAATGCGGCGCAAAACCTGCAGCGGCGACTCTGGTCTGATTTT
GATATCAGTCAAATTCGGTTGCGGTGTGATTGGGGTGAAGTGGACGGTGAACCCGTTCTGAT
CCCAAGGACCACAGGAAAGCGAACGGCGGATTCATATGGCAACAAGGTGTTTTATACCAAAA
CAGGTGTCACCTATTATGAGCGAGAGCGGGGGAAGAAGCGGAGAAAGGTTTTCGCGCAAGAG
GAATTGTCGGAGGAAGAGGCGGAGTTGCTTGTGGAAGCAGACGAGGCAAGGGAGAAATCGGT
CGTTTTGATGCGTGATCCGTCCGGCATTATCAATCGTGGCGACTGGACCAGGCAAAAGGAGT
TTTGGTCGATGGTGAACCAGCGGATTGAAGGATACTTGGTCAAGCAGATTCGCTCGCGCGTT
CGCTTACAAGAAAGTGCGTGTGAAAACACGGGGGATATT

Humanized AaCas12b coding sequence

ATGGCCGTGAAGAGCATGAAGGTGAAGCTGCGCCTGGACAACATGCCCGAGATCCGCGCCGG
CCTGTGGAAGCTGCACACCGAGGTGAACGCCGGCGTGCGCTACTACACCGAGTGGCTGAGCC
TGCTGCGCCAGGAGAACCTGTACCGCCGCAGCCCCAACGGCGACGGCGAGCAGGAGTGCTAC
AAGACCGCCGAGGAGTGCAAGGCCGAGCTGCTGGAGCGCCTGCGCGCCCGCCAGGTGGAGAA
CGGCCACTGCGGCCCCGCCGGCAGCGACGACGAGCTGCTGCAGCTGGCCCGCCAGCTGTACG
AGCTGCTGGTGCCCCAGGCCATCGGCGCCAAGGGCGACGCCCAGCAGATCGCCCGCAAGTTC
CTGAGCCCCCTGGCCGACAAGGACGCCGTGGGCGGCCTGGGCATCGCCAAGGCCGGCAACAA
GCCCCGCTGGGTGCGCATGCGCGAGGCCGGCGAGCCCGGCTGGGAGGAGGAGAAGGCCAAGG
CCGAGGCCCGCAAGAGCACCGACCGCACCGCCGACGTGCTGCGCGCCCTGGCCGACTTCGGC
CTGAAGCCCCTGATGCGCGTGTACACCGACAGCGACATGAGCAGCGTGCAGTGGAAGCCCCT
GCGCAAGGGCCAGGCCGTGCGCACCTGGGACCGCGACATGTTCCAGCAGGCCATCGAGCGCA
TGATGAGCTGGGAGAGCTGGAACCAGCGCGTGGGCGAGGCCTACGCCAAGCTGGTGGAGCAG
AAGAGCCGCTTCGAGCAGAAGAACTTCGTGGGCCAGGAGCACCTGGTGCAGCTGGTGAACCA
GCTGCAGCAGGACATGAAGGAGGCCAGCCACGGCCTGGAGAGCAAGGAGCAGACCGCCCACT
ACCTGACCGGCCGCGCCCTGCGCGGCAGCGACAAGGTGTTCGAGAAGTGGGAGAAGCTGGAC
CCCGACGCCCCCTTCGACCTGTACGACACCGAGATCAAGAACGTGCAGCGCCGCAACACCCG
CCGCTTCGGCAGCCACGACCTGTTCGCCAAGCTGGCCGAGCCCAAGTACCAGGCCCTGTGGC
GCGAGGACGCCAGCTTCCTGACCCGCTACGCCGTGTACAACAGCATCGTGCGCAAGCTGAAC
CACGCCAAGATGTTCGCCACCTTCACCCTGCCCGACGCCACCGCCCACCCCATCTGGACCCG
CTTCGACAAGCTGGGCGGCAACCTGCACCAGTACACCTTCCTGTTCAACGAGTTCGGCGAGG
GCCGCCACGCCATCCGCTTCCAGAAGCTGCTGACCGTGGAGGACGGCGTGGCCAAGGAGGTG
GACGACGTGACCGTGCCCATCAGCATGAGCGCCCAGCTGGACGACCTGCTGCCCCGCGACCC
CCACGAGCTGGTGGCCCTGTACTTCCAGGACTACGGCGCCGAGCAGCACCTGGCCGGCGAGT
TCGGCGGCGCCAAGATCCAGTACCGCCGCGACCAGCTGAACCACCTGCACGCCCGCCGCGGC

GCCCGCGACGTGTACCTGAACCTGAGCGTGCGCGTGCAGAGCCAGAGCGAGGCCCGCGGCGA
GCGCCGCCCCCCCTACGCCGCCGTGTTCCGCCTGGTGGGCGACAACCACCGCGCCTTCGTGC
ACTTCGACAAGCTGAGCGACTACCTGGCCGAGCACCCCGACGACGGCAAGCTGGGCAGCGAG
GGCCTGCTGAGCGGCCTGCGCGTGATGAGCGTGGACCTGGGCCTGCGCACCAGCGCCAGCAT
CAGCGTGTTCCGCGTGGCCCGCAAGGACGAGCTGAAGCCCAACAGCGAGGGCCGCGTGCCCT
TCTGCTTCCCCATCGAGGGCAACGAGAACCTGGTGGCCGTGCACGAGCGCAGCCAGCTGCTG
AAGCTGCCCGGCGAGACCGAGAGCAAGGACCTGCGCGCCATCCGCGAGGAGCGCCAGCGCAC
CCTGCGCCAGCTGCGCACCCAGCTGGCCTACCTGCGCCTGCTGGTGCGCTGCGGCAGCGAGG
ACGTGGGCCGCCGCGAGCGCAGCTGGGCCAAGCTGATCGAGCAGCCCATGGACGCCAACCAG
ATGACCCCCGACTGGCGCGAGGCCTTCGAGGACGAGCTGCAGAAGCTGAAGAGCCTGTACGG
CATCTGCGGCGACCGCGAGTGGACCGAGGCCGTGTACGAGAGCGTGCGCCGCGTGTGGCGCC
ACATGGGCAAGCAGGTGCGCGACTGGCGCAAGGACGTGCGCAGCGGCGAGCGCCCCAAGATC
CGCGGCTACCAGAAGGACGTGGTGGGCGGCAACAGCATCGAGCAGATCGAGTACCTGGAGCG
CCAGTACAAGTTCCTGAAGAGCTGGAGCTTCTTCGGCAAGGTGAGCGGCCAGGTGATCCGCG
CCGAGAAGGGCAGCCGCTTCGCCATCACCCTGCGCGAGCACATCGACCACGCCAAGGAGGAC
CGCCTGAAGAAGCTGGCCGACCGCATCATCATGGAGGCCCTGGGCTACGTGTACGCCCTGGA
CGACGAGCGCGGCAAGGGCAAGTGGGTGGCCAAGTACCCCCCCTGCCAGCTGATCCTGCTGG
AGGAGCTGAGCGAGTACCAGTTCAACAACGACCGCCCCCCCAGCGAGAACAACCAGCTGATG
CAGTGGAGCCACCGCGGCGTGTTCCAGGAGCTGCTGAACCAGGCCCAGGTGCACGACCTGCT
GGTGGGCACCATGTACGCCGCCTTCAGCAGCCGCTTCGACGCCCGCACCGGCGCCCCCGGCA
TCCGCTGCCGCCGCGTGCCCGCCCGCTGCGCCCGCGAGCAGAACCCCGAGCCCTTCCCCTGG
TGGCTGAACAAGTTCGTGGCCGAGCACAAGCTGGACGGCTGCCCCCTGCGCGCCGACGACCT
GATCCCCACCGGCGAGGGCGAGTTCTTCGTGAGCCCCTTCAGCGCCGAGGAGGGCGACTTCC
ACCAGATCCACGCCGACCTGAACGCCGCCCAGAACCTGCAGCGCCGCCTGTGGAGCGACTTC
GACATCAGCCAGATCCGCCTGCGCTGCGACTGGGGCGAGGTGGACGGCGAGCCCGTGCTGAT
CCCCCGCACCACCGGCAAGCGCACCGCCGACAGCTACGGCAACAAGGTGTTCTACACCAAGA
CCGGCGTGACCTACTACGAGCGCGAGCGCGGCAAGAAGCGCCGCAAGGTGTTCGCCCAGGAG
GAGCTGAGCGAGGAGGAGGCCGAGCTGCTGGTGGAGGCCGACGAGGCCCGCGAGAAGAGCGT
GGTGCTGATGCGCGACCCCAGCGGCATCATCAACCGCGGCGACTGGACCCGCCAGAAGGAGT
TCTGGAGCATGGTGAACCAGCGCATCGAGGGCTACCTGGTGAAGCAGATCCGCAGCCGCGTG
CGCCTGCAGGAGAGCGCCTGCGAGAACACCGGCGACATC

AaCas12b protein sequence

MAVKSMKVKLRLDNMPEIRAGLWKLHTEVNAGVRYYTEWLSLLRQENLYRRSPNGDGEQECY
KTAEECKAELLERLRARQVENGHCGPAGSDDELLQLARQLYELLVPQAIGAKGDAQQIARKF
LSPLADKDAVGGLGIAKAGNKPRWVRMREAGEPGWEEEKAKAEARKSTDRTADVLRALADFG
LKPLMRVYTDSDMSSVQWKPLRKGQAVRTWDRDMFQQAIERMMSWESWNQRVGEAYAKLVEQ
KSRFEQKNFVGQEHLVQLVNQLQQDMKEASHGLESKEQTAHYLTGRALRGSDKVFEKWEKLD
PDAPFDLYDTEIKNVQRRNTRRFGSHDLFAKLAEPKYQALWREDASFLTRYAVYNSIVRKLN
HAKMFATFTLPDATAHPIWTRFDKLGGNLHQYTFLFNEFGEGRHAIRFQKLLTVEDGVAKEV
DDVTVPISMSAQLDDLLPRDPHELVALYFQDYGAEQHLAGEFGGAKIQYRRDQLNHLHARRG
ARDVYLNLSVRVQSQSEARGERRPPYAAVFRLVGDNHRAFVHFDKLSDYLAEHPDDGKLGSE
GLLSGLRVMSVDLGLRTSASISVFRVARKDELKPNSEGRVPFCFPIEGNENLVAVHERSQLL
KLPGETESKDLRAIREERQRTLRQLRTQLAYLRLLVRCGSEDVGRRERSWAKLIEQPMDANQ

MTPDWREAFEDELQKLKSLYGICGDREWTEAVYESVRRVWRHMGKQVRDWRKDVRSGERPKI
RGYQKDVVGGNSIEQIEYLERQYKFLKSWSFFGKVSGQVIRAEKGSRFAITLREHIDHAKED
RLKKLADRIIMEALGYVYALDDERGKGKWVAKYPPCQLILLEELSEYQFNNDRPPSENNQLM
QWSHRGVFQELLNQAQVHDLLVGTMYAAFSSRFDARTGAPGIRCRRVPARCAREQNPEPFPW
WLNKFVAEHKLDGCPLRADDLIPTGEGEFFVSPFSAEEGDFHQIHADLNAAQNLQRRLWSDF
DISQIRLRCDWGEVDGEPVLIPRTTGKRTADSYGNKVFYTKTGVTYYERERGKKRRKVFAQE
ELSEEEAELLVEADEAREKSVVLMRDPSGIINRGDWTRQKEFWSMVNQRIEGYLVKQIRSRV
RLQESACENTGDI

Cas12b coding sequence from *Alicyclobacillus kakegawensis* NBRC 103104 (GeneBank ID: NZ_BCRP01000027.1)

ATGGCTGTAAAATCTATTAAGGTCAAGTTGCGGTTGTCAGAGTGCCCAGACATCCTGGCTGG
CATGTGGCAGCTCCACCGGGCGACAAACGCGGGGGTTCGATACTACACAGAATGGGTGAGCT
TGATGCGCCAGGAGATCCTCTACTCGCGCGGGCCGGACGGCGGTCAGCAGTGCTACATGACC
GCGGAGGATTGCCAACGCGAGCTGCTGCGGCGGCTGCGCAATCGCCAGCTCCATAATGGCCG
CCAGGACCAGCCCGGTACAGATGCAGACCTACTGGCAATCAGTAGGAGACTCTATGAAATTC
TGGTCCTGCAATCCATCGGCAAGAGGGGGGACGCCCAGCAGATAGCGAGCAGCTTCCTCAGC
CCTCTGGTCGATCCGAACTCCAAAGGTGGGCGGGGTGAAGCCAAGTCCGGTCGAAAGCCTGC
GTGGCAGAAGATGCGCGATCAAGGTGATCCTCGTTGGGTTGCGGCAAGGGAAAAGTACGAGC
AACGCAAGGCGGTTGATCCATCTAAAGAAATCCTGAATTCATTGGACGCCCTGGGTCTCAGG
CCGCTATTTGCGGTCTTCACGGAGACCTACAGGTCGGGAGTCGATTGGAAGCCGCTCGGCAA
AAGCCAAGGTGTGCGCACATGGGACCGTGACATGTTCCAGCAGGCCCTCGAGCGCCTGATGT
CCTGGGAGTCTTGGAACCGCCGCGTGGGCGAGGAGTACGCCCGTCTTTTCCAACAGAAGATG
AAGTTCGAGCAGGAACACTTCGCGGAACAGTCTCATCTGGTTAAACTGGCGCGCGCGTTGGA
GGCGGACATGCGCGCCGCTTCACAGGGCTTCGAAGCCAAACGCGGCACTGCGCACCAGATCA
CAAGACGGGCGCTGCGCGGGGCGGATCGGGTATTTGAGATATGGAAGAGTATTCCAGAGGAA
GCTTTGTTCTCCCAATATGATGAAGTGATTCGACAGGTCCAGGCGGAGAAAAGACGGGACTT
TGGGTCCCATGATCTGTTCGCCAAGTTGGCGGAACCGAAGTATCAGCCCCTGTGGCGCGCCG
ACGAGACCTTTTTGACGCGCTACGCCCTGTACAATGGAGTCTTGCGGGATTTAGAGAAAGCG
AGACAGTTCGCCACGTTCACGCTGCCGGATGCCTGCGTCAATCCAATTTGGACGCGTTTTGA
AAGCAGCCAGGGGAGCAATCTGCATAAATATGAATTTCTCTTTGACCACCTGGGACCCGGAC
GGCACGCGGTGCGTTTTCAGAGGCTGCTGGTGGTAGAGAGCGAAGGTGCGAAGGAGAGGGAC
TCGGTGGTGGTGCCAGTCGCGCCATCCGGGCAACTGGACAAGCTTGTCCTGCGTGAAGAAGA
GAAATCAAGCGTTGCCTTACACCTTCATGACACAGCCCGGCCGGACGGTTTCATGGCAGAAT
GGGCGGGGGCGAAGCTGCAATATGAACGCAGTACCTTGGCACGCAAGGCGCGCCGTGATAAG
CAAGGGATGCGGTCGTGGCGTAGGCAGCCGTCTATGCTGATGTCTGCGGCACAGATGTTGGA
AGACGCAAAGCAAGCCGGAGACGTGTATCTGAACATCAGTGTGCGTGTGAAGAGCCCCAGTG
AAGTCCGCGGCCAGAGGCGGCCTCCTTACGCGGCCCTGTTTCGGATAGACGATAAACAGCGG
CGTGTGACCGTAAATTACAACAAACTGTCGGCTTACCTAGAGGAACATCCGGATAAACAGAT
TCCAGGCGCACCTGGGCTCCTTTCCGGTCTTCGGGTAATGAGCGTCGACCTTGGGTTGCGCA
CCTCCGCTTCCATCAGTGTGTTCCGTGTGGCAAAGAAGGAAGAGGTGGAAGCGCTGGGCGAC
GGTCGTCCCCCTCATTATTATCCCATCCATGGCACTGACGACCTGGTGGCGGTGCACGAGCG
CTCACATTTGATTCAAATGCCAGGCGAAACCGAAACGAAACAGCTGCGCAAGTTGCGTGAGG

AACGGCAGGCTGTCTTGCGTCCACTGTTCGCTCAACTGGCCCTGCTACGGTTGCTGGTCCGG
TGTGGTGCAGCCGACGAGCGGATTCGTACACGCAGTTGGCAGCGCTTGACGAAGCAGGGGCG
TGAGTTTACGAAGCGATTGACGCCGTCCTGGCGGGAGGCGTTGGAATTGGAGTTAACTCGCT
TGGAGGCGTATTGCGGTAGGGTTCCAGACGACGAATGGAGCCGCATCGTTGATAGAACGGTA
ATCGCTTTGTGGCGTCGCATGGGAAAACAGGTGCGCGATTGGCGTAAACAGGTGAAATCCGG
TGCGAAAGTCAAGGTCAAGGGGTACCAGCTGGATGTAGTCGGCGGCAACTCGCTGGCGCAAA
TCGATTATCTCGAACAGCAGTACAAGTTTCTGCGGCGCTGGAGCTTCTTTGCGCGGGCCAGC
GGTCTGGTTGTGCGGGCGGATCGCGAATCGCATTTCGCAGTCGCTTTACGCCAGCACATTGA
AAATGCCAAGCGGGATCGGCTGAAAAAGTTGGCGGACCGCATCCTGATGGAGGCGCTGGGCT
ACGTGTATGAAGCTTCCGGGCCGCGCGAAGGACAGTGGACGGCGCAGCATCCGCCGTGCCAG
TTGATTATCTTGGAGGAATTAAGCGCGTACCGGTTCAGTGACGACCGTCCGCCGAGCGAGAA
CAGTAAATTGATGGCTTGGGGGCATCGGGGAATTTTGGAGGAGTTGGTCAACCAAGCACAGG
TTCACGACGTGTTAGTGGGGACGGTGTACGCCGCTTTTTCGTCCCGCTTCGATGCCCGCACA
GGCGCCCCTGGAGTGCGCTGCCGCCGGGTACCCGCACGTTTTGTCGGCGCGACGGTGGATGA
TTCACTGCCGCTTTGGCTCACAGAGTTTCTGGACAAGCACAGGCTGGATAAAAACCTCCTGC
GGCCTGACGATGTGATTCCGACCGGAGAGGGTGAGTTTTTGGTTTCTCCGTGTGGCGAGGAA
GCGGCTCGGGTTCGGCAGGTGCACGCCGACATCAACGCGGCGCAAAACCTGCAGCGGAGGCT
GTGGCAGAATTTTGACATTACAGAGCTGCGTCTGCGCTGCGATGTGAAGATGGGTGGCGAAG
GAACGGTGCTGGTACCAAGGGTCAACAACGCCCGCGCCAAACAACTGTTTGGAAAGAAGGTG
TTGGTTTCGCAAGATGGCGTGACGTTCTTTGAACGCAGTCAAACAGGTGGGAAACCGCACAG
CGAGAAGCAGACGGATTTGACCGACAAGGAACTAGAACTAATTGCGGAGGCGGACGAGGCGC
GCGCCAAGTCGGTCGTCCTCTTTCGCGATCCGTCCGGGCACATCGGCAAGGGCCACTGGATT
CGCCAAAGGGAGTTTTGGTCGTTGGTGAAGCAAAGGATTGAATCGCACACGGCGGAAAGGAT
ACGGGTTCGCGGCGTCGGTAGCTCGCTGGAT

Humanized AkCas12b coding sequence

ATGGCCGTGAAGAGCATCAAGGTGAAGCTGCGCCTGAGCGAGTGCCCCGACATCCTGGCCGG
CATGTGGCAGCTGCACCGCGCCACCAACGCCGGCGTGCGCTACTACACCGAGTGGGTGAGCC
TGATGCGCCAGGAGATCCTGTACAGCCGCGGCCCCGACGGCGGCCAGCAGTGCTACATGACC
GCCGAGGACTGCCAGCGCGAGCTGCTGCGCCGCCTGCGCAACCGCCAGCTGCACAACGGCCG
CCAGGACCAGCCCGGCACCGACGCCGACCTGCTGGCCATCAGCCGCCGCCTGTACGAGATCC
TGGTGCTGCAGAGCATCGGCAAGCGCGGCGACGCCCAGCAGATCGCCAGCAGCTTCCTGAGC
CCCCTGGTGGACCCCAACAGCAAGGGCGGCCGCGGCGAGGCCAAGAGCGGCCGCAAGCCCGC
CTGGCAGAAGATGCGCGACCAGGGCGACCCCCGCTGGGTGGCCGCCCGCGAGAAGTACGAGC
AGCGCAAGGCCGTGGACCCCAGCAAGGAGATCCTGAACAGCCTGGACGCCCTGGGCCTGCGC
CCCCTGTTCGCCGTGTTCACCGAGACCTACCGCAGCGGCGTGGACTGGAAGCCCCTGGGCAA
GAGCCAGGGCGTGCGCACCTGGGACCGCGACATGTTCCAGCAGGCCCTGGAGCGCCTGATGA
GCTGGGAGAGCTGGAACCGCCGCGTGGGCGAGGAGTACGCCCGCCTGTTCCAGCAGAAGATG
AAGTTCGAGCAGGAGCACTTCGCCGAGCAGAGCCACCTGGTGAAGCTGGCCCGCGCCCTGGA
GGCCGACATGCGCGCCGCCAGCCAGGGCTTCGAGGCCAAGCGCGGCACCGCCCACCAGATCA
CCCGCCGCGCCCTGCGCGGCGCCGACCGCGTGTTCGAGATCTGGAAGAGCATCCCCGAGGAG
GCCCTGTTCAGCCAGTACGACGAGGTGATCCGCCAGGTGCAGGCCGAGAAGCGCCGCGACTT
CGGCAGCCACGACCTGTTCGCCAAGCTGGCCGAGCCCAAGTACCAGCCCCTGTggcgcgccG
ACGAGACCTTCCTGACCCGCTACGCCCTGTACAACGGCGTGCTGCGCGACCTGGAGAAGGCC

CGCCAGTTCGCCACCTTCACCCTGCCCGACGCCTGCGTGAACCCCATCTGGACCCGCTTCGA
GAGCAGCCAGGGCAGCAACCTGCACAAGTACGAGTTCCTGTTCGACCACCTGGGCCCCGGCC
GCCACGCCGTGCGCTTCCAGCGCCTGCTGGTGGTGGAGAGCGAGGGCGCCAAGGAGCGCGAC
AGCGTGGTGGTGCCCGTGGCCCCCAGCGGCCAGCTGGACAAGCTGGTGCTGCGCGAGGAGGA
GAAGAGCAGCGTGGCCCTGCACCTGCACGACACCGCCCGCCCCGACGGCTTCATGGCCGAGT
GGGCCGGCGCCAAGCTGCAGTACGAGCGCAGCACCCTGGCCCGCAAGGCCCGCCGCGACAAG
CAGGGCATGCGCAGCTGGCGCCGCCAGCCCAGCATGCTGATGAGCGCCGCCCAGATGCTGGA
GGACGCCAAGCAGGCCGGCGACGTGTACCTGAACATCAGCGTGCGCGTGAAGAGCCCCAGCG
AGGTGCGCGGCCAGCGCCGCCCCCCCTACGCCGCCCTGTTCCGCATCGACGACAAGCAGCGC
CGCGTGACCGTGAACTACAACAAGCTGAGCGCCTACCTGGAGGAGCACCCCGACAAGCAGAT
CCCCGGCGCCCCCGGCCTGCTGAGCGGCCTGCGCGTGATGAGCGTGGACCTGGGCCTGCGCA
CCAGCGCCAGCATCAGCGTGTTCCGCGTGGCCAAGAAGGAGGAGGTGGAGGCCCTGGGCGAC
GGCCGCCCCCCCCACTACTACCCCATCCACGGCACCGACGACCTGGTGGCCGTGCACGAGCG
CAGCCACCTGATCCAGATGCCCGGCGAGACCGAGACCAAGCAGCTGCGCAAGCTGCGCGAGG
AGCGCCAGGCCGTGCTGCGCCCCCTGTTCGCCCAGCTGGCCCTGCTGCGCCTGCTGGTGCGC
TGCGGCGCCGCCGACGAGCGCATCCGCACCCGCAGCTGGCAGCGCCTGACCAAGCAGGGCCG
CGAGTTCACCAAGCGCCTGACCCCCAGCTGGCGCGAGGCCCTGGAGCTGGAGCTGACCCGCC
TGGaggcctACTGCGGCCGCGTGCCCGACGACGAGTGGAGCCGCATCGTGGACCGCACCGTG
ATCGCCCTGTGGCGCCGCATGGGCAAGCAGGTGCGCGACTGGCGCAAGCAGGTGAAGAGCGG
CGCCAAGGTGAAGGTGAAGGGCTACCAGCTGGACGTGGTGGGCGGCAACAGCCTGGCCCAGA
TCGACTACCTGGAGCAGCAGTACAAGTTCCTGCGCCGCTGGAGCTTCTTCGCCCGCGCCAGC
GGCCTGGTGGTGCGCGCCGACCGCGAGAGCCACTTCGCCGTGGCCCTGCGCCAGCACATCGA
GAACGCCAAGCGCGACCGCCTGAAGAAGCTGGCCGACCGCATCCTGATGGAGGCCCTGGGCT
ACGTGTACGAGGCCAGCGGCCCCCGCGAGGGCCAGTGGACCGCCCAGCACCCCCCCTGCCAG
CTGATCATCCTGGAGGAGCTGAGCGCCTACCGCTTCAGCGACGACCGCCCCCCCAGCGAGAA
CAGCAAGCTGATGGCCTGGGGCCACCGCGGCATCCTGGAGGAGCTGGTGAACCAGGCCCAGG
TGCACGACGTGCTGGTGGGCACCGTGTACGCCGCCTTCAGCAGCCGCTTCGACGCCCGCACC
GGCGCCCCCGGCGTGCGCTGCCGCCGCGTGCCCGCCCGCTTCGTGGGCGCCACCGTGGACGA
CAGCCTGCCCCTGTGGCTGACCGAGTTCCTGGACAAGCACCGCCTGGACAAGAACCTGCTGC
GCCCCGACGACGTGATCCCCACCGGCGAGGGCGAGTTCCTGGTGAGCCCCTGCGGCGAGGAG
GCCGCCCGCGTGCGCCAGGTGCACGCCGACATCAACGCCGCCCAGAACCTGCAGCGCCGCCT
GTGGCAGAACTTCGACATCACCGAGCTGCGCCTGCGCTGCGACGTGAAGATGGGCGGCGAGG
GCACCGTGCTGGTGCCCCGCGTGAACAACGCCCGCGCCAAGCAGCTGTTCGGCAAGAAGGTG
CTGGTGAGCCAGGACGGCGTGACCTTCTTCGAGCGCAGCCAGACCGGCGGCAAGCCCCACAG
CGAGAAGCAGACCGACCTGACCGACAAGGAGCTGGAGCTGATCGCCGAGGCCGACGAGGCCC
GCGCCAAGAGCGTGGTGCTGTTCCGCGACCCCAGCGGCCACATCGGCAAGGGCCACTGGATC
CGCCAGCGCGAGTTCTGGAGCCTGGTGAAGCAGCGCATCGAGAGCCACACCGCCGAGCGCAT
CCGCGTGCGCGGCGTGGGCAGCAGCCTGGAC

AkCas12b protein sequence

MAVKSIKVKLRLSECPDILAGMWQLHRATNAGVRYYTEWVSLMRQEILYSRGPDGGQQCYMT
AEDCQRELLRRLRNRQLHNGRQDQPGTDADLLAISRRLYEILVLQSIGKRGDAQQIASSFLS
PLVDPNSKGGRGEAKSGRKPAWQKMRDQGDPRWVAAREKYEQRKAVDPSKEILNSLDALGLR
PLFAVFTETYRSGVDWKPLGKSQGVRTWDRDMFQQALERLMSWESWNRRVGEEYARLFQQKM

KFEQEHFAEQSHLVKLARALEADMRAASQGFEAKRGTAHQITRRALRGADRVFEIWKSIPEE
ALFSQYDEVIRQVQAEKRRDFGSHDLFAKLAEPKYQPLWRADETFLTRYALYNGVLRDLEKA
RQFATFTLPDACVNPIWTRFESSQGSNLHKYEFLFDHLGPGRHAVRFQRLLVVESEGAKERD
SVVVPVAPSGQLDKLVLREEEKSSVALHLHDTARPDGFMAEWAGAKLQYERSTLARKARRDK
QGMRSWRRQPSMLMSAAQMLEDAKQAGDVYLNISVRVKSPSEVRGQRRPPYAALFRIDDKQR
RVTVNYNKLSAYLEEHPDKQIPGAPGLLSGLRVMSVDLGLRTSASISVFRVAKKEEVEALGD
GRPPHYYPIHGTDDLVAVHERSHLIQMPGETETKQLRKLREERQAVLRPLFAQLALLRLLVR
CGAADERIRTRSWQRLTKQGREFTKRLTPSWREALELELTRLEAYCGRVPDDEWSRIVDRTV
IALWRRMGKQVRDWRKQVKSGAKVKVKGYQLDVVGGNSLAQIDYLEQQYKFLRRWSFFARAS
GLVVRADRESHFAVALRQHIENAKRDRLKKLADRILMEALGYVYEASGPREGQWTAQHPPCQ
LIILEELSAYRFSDDRPPSENSKLMAWGHRGILEELVNQAQVHDVLVGTVYAAFSSRFDART
GAPGVRCRRVPARFVGATVDDSLPLWLTEFLDKHRLDKNLLRPDDVIPTGEGEFLVSPCGEE
AARVRQVHADINAAQNLQRRLWQNFDITELRLRCDVKMGGEGTVLVPRVNNARAKQLFGKKV
LVSQDGVTFFERSQTGGKPHSEKQTDLTDKELELIAEADEARAKSVVLFRDPSGHIGKGHWI
RQREFWSLVKQRIESHTAERIRVRGVGSSLD

Cas12b coding sequence from *Alicyclobacillus macrosporangiidus* strain DSM 17980 (GeneBank ID: FPBV01000001.1)

ATGAACGTGGCCGTGAAATCCATCAAGGTCAAGCTGATGTTGGGTCACCTCCCGGAAATCCG
GGAAGGGCTGTGGCACCTGCACGAGGCAGTCAACTTGGGAGTTCGCTACTACACGGAATGGC
TGGCCCTCCTGCGCCAGGGAAACCTGTACCGGCGAGGCAAGGATGGTGCGCAGGAGTGCTAC
ATGACGGCGGAGCAGTGCCGGCAGGAGTTGCTCGTGCGGCTGCGGGATCGACAGAAGCGCAA
TGGGCATACCGGGGACCCGGGCACGGACGAGGAGTTGCTTGGCGTCGCCAGGCGGCTGTATG
AGCTGTTGGTGCCGCAGTCCGTCGGGAAAAAGGGGCAGGCGCAGATGCTGGCCAGCGGTTTC
CTGAGTCCATTGGCGGATCCGAAGTCCGAGGGAGGAAAGGGCACGTCCAAGTCTGGGCGCAA
GCCGGCGTGGATGGGCATGAAGGAGGCCGGGGATTCGCGTTGGGTGGAGGCGAAGGCCCGGT
ACGAAGCCAATAAAGCGAAGGATCCGACGAAGCAGGTGATTGCGTCTCTCGAGATGTACGGC
CTGAGGCCGTTGTTCGACGTGTTCACGGAGACGTACAAAACGATCCGCTGGATGCCGTTGGG
CAAACATCAAGGCGTGCGCGCCTGGGATCGCGACATGTTCCAACAGTCCTTGGAACGCCTGA
TGTCCTGGGAATCCTGGAACGAACGCGTCGGGGCGGAGTTTGCCCGGTTGGTGGACCGGCGG
GACAGGTTCCGGGAAAAGCACTTCACCGGGCAGGAGCACCTGGTCGCGCTGGCGCAACGGCT
GGAACAGGAGATGAAAGAAGCGTCGCCGGGGTTCGAGTCCAAATCCTCTCAGGCGCACCGGA
TCACAAAGCGCGCGCTGCGGGGGGCGGACGGCATCATCGATGACTGGTTGAAGCTGTCTGAA
GGGGAACCGGTGGATCGATTCGATGAGATCCTCCGGAAGCGTCAGGCCCAGAATCCTCGCAG
GTTCGGATCCCATGATCTCTTCTTGAAGTTGGCGGAACCTGTCTTTCAGCCGCTGTGGCGGG
AGGACCCAAGTTTCTTGTCGCGCTGGGCGTCGTATAACGAAGTGTTGAACAAACTCGAGGAC
GCGAAGCAGTTCGCCACCTTTACGCTGCCAAGTCCGTGCTCGAACCCGGTGTGGGCCCGGTT
CGAGAACGCGGAGGGTACGAACATCTTCAAATACGACTTTCTCTTCGACCACTTCGGAAAGG
GACGACACGGAGTTCGGTTTCAGCGGATGATCGTGATGCGGGACGGCGTGCCGACCGAGGTG
GAGGGGATTGTCGTGCCGATTGCACCCTCCCGGCAGTTGGACGCGCTCGCCCCCAACGACGC
CGCGTCGCCGATTGACGTTTTCGTCGGCGATCCCGCGGCCCCGGGCGCATTTCGCGGACAGT
TCGGGGGTGCGAAGATCCAGTACCGTCGATCGGCGCTGGTACGCAAAGGACGGCGCGAAGAG
AAGGCGTACCTTTGTGGATTCCGCTTGCCGTCGCAACGCCGGACGGGGACGCCTGCCGACGA

CGCGGGCGAGGTGTTTTTGAACCTCAGCCTGCGCGTGGAGAGCCAGTCGGAGCAGGCAGGAC
GTCGCAATCCTCCCTATGCGGCCGTGTTTCACATTTCCGACCAAACCCGCCGCGTGATCGTG
AGATATGGCGAAATTGAGCGGTATCTGGCGGAGCACCCGGACACCGGCATCCCGGGGTCACG
GGGGCTCACGAGCGGCCTGCGTGTGATGAGCGTAGACCTGGGATTGCGGACGTCCGCCGCTA
TCTCGGTGTTTCGCGTGGCGCACAGGGACGAGCTCACGCCCGATGCGCATGGACGCCAGCCC
TTCTTTTTCCCCATTCATGGTATGGACCATCTCGTCGCCCTCCATGAGCGCTCCCACCTCAT
CCGCCTGCCGGGTGAGACGGAGTCCAAGAAAGTTCGCAGCATCCGGGAGCAGAGGCTGGATC
GGTTGAACCGGCTGCGGTCCCAGATGGCGTCGTTGCGGCTACTCGTTCGAACCGGGGTCCTG
GATGAGCAGAAACGGGATCGCAACTGGGAACGCCTTCAGTCCTCCATGGAACGCGGCGGAGA
GCGGATGCCATCCGATTGGTGGGACTTGTTCCAAGCACAGGTGAGGTATCTGGCTCAGCATC
GGGATGCTTCCGGCGAGGCGTGGGGGCGGATGGTACAGGCGGCAGTCCGCACCTTATGGAGG
CAACTGGCCAAGCAGGTGCGCGACTGGCGAAAAGAGGTCCGTCGGAATGCAGACAAGGTGAA
GATCAGGGGAATTGCCCGGGACGTTCCAGGGGGCCATTCCCTTGCGCAGCTGGACTATCTCG
AACGACAGTACCGGTTCTTGCGGAGTTGGAGCGCCTTCTCCGTGCAGGCCGGCCAGGTGGTC
CGCGCCGAGCGGGATTCCCGCTTCGCCGTGGCGCTGCGGGAGCATATCGACAACGGCAAGAA
GGATCGGTTGAAGAAATTGGCGGATCGGATCCTCATGGAAGCCTTGGGATACGTGTACGTCA
CGGACGGCCGCCGCGCCGGACAGTGGCAGGCTGTGTATCCGCCTTGTCAGCTCGTGCTGTTG
GAGGAGTTGAGCGAGTACCGGTTCAGCAATGACCGCCCGCCGAGCGAAAACAGTCAATTGAT
GGTGTGGAGCCACCGCGGCGTGTTGGAGGAGTTGATCCACCAGGCGCAGGTTCACGACGTGC
TGGTGGGCACCATTCCGGCGGCGTTCTCGTCCCGGTTCGATGCTCGGACGGGCGCACCGGGC
ATCCGGTGCAGGAGAGTGCCGTCGATCCCGCTGAAAGATGCCCCGTCCATCCCCATTTGGCT
GTCTCACTATCTCAAGCAAACGGAACGGGACGCTGCTGCTCTGCGGCCGGGCGAGCTCATCC
CGACCGGTGACGGGGAGTTTCTGGTGACCCCCGCGGGCCGCGGGGCATCCGGTGTTCGCGTC
GTCCATGCGGACATCAACGCGGCGCACAATCTGCAGAGGCGGTTGTGGGAGAACTTCGACCT
CAGCGACATTCGGGTTCGGTGTGACCGGCGTGAAGGGAAGGATGGCACGGTCGTCCTCATCC
CGAGGCTGACGAACCAGCGCGTGAAGGAGAGGTACAGCGGGGTCATCTTCACGTCGGAGGAC
GGTGTCAGCTTTACAGTGGGAGACGCCAAAACCCGGCGCCGATCTTCGGCTTCGCAGGGGGA
AGGTGACGACCTGTCGGACGAAGAGCAGGAGTTGCTCGCTGAGGCGGATGACGCGCGCGAGA
GGTCTGTCGTCCTGTTCCGTGACCCGTCTGGTTTCGTCAATGGGGGGCGATGGACCGCGCAA
CGGGCGTTTTGGGGCATGGTCCACAACCGGATCGAAACCTTGTTGGCCGAGCGGTTTTCTGT
GTCGGGAGCGGCCGAAAAGGTGAGAGGA

Humanized AmCas12b coding sequence

ATGAACGTGGCCGTGAAGAGCATCAAGGTGAAGCTGATGCTGGGCCACCTGCCCGAGATCCG
CGAGGGCCTGTGGCACCTGCACGAGGCCGTGAACCTGGGCGTGCGCTACTACACCGAGTGGC
TGGCCCTGCTGCGCCAGGGCAACCTGTACCGCCGCGGCAAGGACGGCGCCCAGGAGTGCTAC
ATGACCGCCGAGCAGTGCCGCCAGGAGCTGCTGGTGCGCCTGCGCGACCGCCAGAAGCGCAA
CGGCCACACCGGCGACCCCGGCACCGACGAGGAGCTGCTGGGCGTGGCCCGCCGCCTGTACG
AGCTGCTGGTGCCCCAGAGCGTGGGCAAGAAGGGCCAGGCCCAGATGCTGGCCAGCGGCTTC
CTGAGCCCCCTGGCCGACCCCAAGAGCGAGGGCGGCAAGGGCACCAGCAAGAGCGGCCGCAA
GCCCGCCTGGATGGGCATGAAGGAGGCCGGCGACAGCCGCTGGGTGGAGGCCAAGGCCCGCT
ACGAGGCCAACAAGGCCAAGGACCCCACCAAGCAGGTGATCGCCAGCCTGGAGATGTACGGC
CTGCGCCCCCTGTTCGACGTGTTCACCGAGACCTACAAGACCATCCGCTGGATGCCCCTGGG
CAAGCACCAGGGCGTGCGCGCCTGGGACCGCGACATGTTCCAGCAGAGCCTGGAGCGCCTGA

TGAGCTGGGAGAGCTGGAACGAGCGCGTGGGCGCCGAGTTCGCCCGCCTGGTGGACCGCCGC
GACCGCTTCCGCGAGAAGCACTTCACCGGCCAGGAGCACCTGGTGGCCCTGGCCCAGCGCCT
GGAGCAGGAGATGAAGGAGGCCAGCCCCGGCTTCGAGAGCAAGAGCAGCCAGGCCCACCGCA
TCACCAAGCGCGCCCTGCGCGGCGCCGACGGCATCATCGACGACTGGCTGAAGCTGAGCGAG
GGCGAGCCCGTGGACCGCTTCGACGAGATCCTGCGCAAGCGCCAGGCCCAGAACCCCCGCCG
CTTCGGCAGCCACGACCTGTTCCTGAAGCTGGCCGAGCCCGTGTTCCAGCCCCTGTGGCGCG
AGGACCCCAGCTTCCTGAGCCGCTGGGCCAGCTACAACGAGGTGCTGAACAAGCTGGAGGAC
GCCAAGCAGTTCGCCACCTTCACCCTGCCCAGCCCCTGCAGCAACCCCGTGTGGGCCCGCTT
CGAGAACGCCGAGGGCACCAACATCTTCAAGTACGACTTCCTGTTCGACCACTTCGGCAAGG
GCCGCCACGGCGTGCGCTTCCAGCGCATGATCGTGATGCGCGACGGCGTGCCCACCGAGGTG
GAGGGCATCGTGGTGCCCATCGCCCCCAGCCGCCAGCTGGACGCCCTGGCCCCCAACGACGC
CGCCAGCCCCATCGACGTGTTCGTGGGCGACCCCGCCGCCCCCGGCGCCTTCCGCGGCCAGT
TCGGCGGCGCCAAGATCCAGTACCGCCGCAGCGCCCTGGTGCGCAAGGGCCGCCGCGAGGAG
AAGGCCTACCTGTGCGGCTTCCGCCTGCCCAGCCAGCGCCGCACCGGCACCCCCGCCGACGA
CGCCGGCGAGGTGTTCCTGAACCTGAGCCTGCGCGTGGAGAGCCAGAGCGAGCAGGCCGGCC
GCCGCAACCCCCCCTACGCCGCCGTGTTCCACATCAGCGACCAGACCCGCCGCGTGATCGTG
CGCTACGGCGAGATCGAGCGCTACCTGGCCGAGCACCCCGACACCGGCATCCCCGGCAGCCG
CGGCCTGACCAGCGGCCTGCGCGTGATGAGCGTGGACCTGGGCCTGCGCACCAGCGCCGCCA
TCAGCGTGTTCCGCGTGGCCCACCGCGACGAGCTGACCCCCGACGCCCACGGCCGCCAGCCC
TTCTTCTTCCCCATCCACGGCATGGACCACCTGGTGGCCCTGCACGAGCGCAGCCACCTGAT
CCGCCTGCCCGGCGAGACCGAGAGCAAGAAGGTGCGCAGCATCCGCGAGCAGCGCCTGGACC
GCCTGAACCGCCTGCGCAGCCAGATGGCCAGCCTGCGCCTGCTGGTGCGCACCGGCGTGCTG
GACGAGCAGAAGCGCGACCGCAACTGGGAGCGCCTGCAGAGCAGCATGGAGCGCGGCGGCGA
GCGCATGCCCAGCGACTGGTGGGACCTGTTCCAGGCCCAGGTGCGCTACCTGGCCCAGCACC
GCGACGCCAGCGGCGAGGCCTGGGGCCGCATGGTGCAGGCCGCCGTGCGCACCCTGTGGCGC
CAGCTGGCCAAGCAGGTGCGCGACTGGCGCAAGGAGGTGCGCCGCAACGCCGACAAGGTGAA
GATCCGCGGCATCGCCCGCGACGTGCCCGGCGGCCACAGCCTGGCCCAGCTGGACTACCTGG
AGCGCCAGTACCGCTTCCTGCGCAGCTGGAGCGCCTTCAGCGTGCAGGCCGGCCAGGTGGTG
CGCGCCGAGCGCGACAGCCGCTTCGCCGTGGCCCTGCGCGAGCACATCGACAACGGCAAGAA
GGACCGCCTGAAGAAGCTGGCCGACCGCATCCTGATGGAGGCCCTGGGCTACGTGTACGTGA
CCGACGGCCGCCGCGCCGGCCAGTGGCAGGCCGTGTACCCCCCCTGCCAGCTGGTGCTGCTG
GAGGAGCTGAGCGAGTACCGCTTCAGCAACGACCGCCCCCCCAGCGAGAACAGCCAGCTGAT
GGTGTGGAGCCACCGCGGCGTGCTGGAGGAGCTGATCCACCAGGCCCAGGTGCACGACGTGC
TGGTGGGCACCATCCCCGCCGCCTTCAGCAGCCGCTTCGACGCCCGCACCGGCGCCCCCGGC
ATCCGCTGCCGCCGCGTGCCCAGCATCCCCCTGAAGGACGCCCCCAGCATCCCCATCTGGCT
GAGCCACTACCTGAAGCAGACCGAGCGCGACGCCGCCGCCCTGCGCCCCGGCGAGCTGATCC
CCACCGGCGACGGCGAGTTCCTGGTGACCCCCGCCGGCCGCGGCGCCAGCGGCGTGCGCGTG
GTGCACGCCGACATCAACGCCGCCCACAACCTGCAGCGCCGCCTGTGGGAGAACTTCGACCT
GAGCGACATCCGCGTGCGCTGCGACCGCCGCGAGGGCAAGGACGGCACCGTGGTGCTGATCC
CCCGCCTGACCAACCAGCGCGTGAAGGAGCGCTACAGCGGCGTGATCTTCACCAGCGAGGAC
GGCGTGAGCTTCACCGTGGGCGACGCCAAGACCCGCCGCCGCAGCAGCGCCAGCCAGGGCGA
GGGCGACGACCTGAGCGACGAGGAGCAGGAGCTGCTGGCCGAGGCCGACGACGCCCGCGAGC
GCAGCGTGGTGCTGTTCCGCGACCCCAGCGGCTTCGTGAACGGCGGCCGCTGGACCGCCCAG
CGCGCCTTCTGGGGCATGGTGCACAACCGCATCGAGACCCTGCTGGCCGAGCGCTTCAGCGT

GAGCGGCGCCGCCGAGAAGGTGCGCGGC

AmCas12b protein sequence

MNVAVKSIKVKLMLGHLPEIREGLWHLHEAVNLGVRYYTEWLALLRQGNLYRRGKDGAQECY
MTAEQCRQELLVRLRDRQKRNGHTGDPGTDEELLGVARRLYELLVPQSVGKKGQAQMLASGF
LSPLADPKSEGGKGTSKSGRKPAWMGMKEAGDSRWVEAKARYEANKAKDPTKQVIASLEMYG
LRPLFDVFTETYKTIRWMPLGKHQGVRAWDRDMFQQSLERLMSWESWNERVGAEFARLVDRR
DRFREKHFTGQEHLVALAQRLEQEMKEASPGFESKSSQAHRITKRALRGADGIIDDWLKLSE
GEPVDRFDEILRKRQAQNPRRFGSHDLFLKLAEPVFQPLWREDPSFLSRWASYNEVLNKLED
AKQFATFTLPSPCSNPVWARFENAEGTNIFKYDFLFDHFGKGRHGVRFQRMIVMRDGVPTEV
EGIVVPIAPSRQLDALAPNDAASPIDVFVGDPAAPGAFRGQFGGAKIQYRRSALVRKGRREE
KAYLCGFRLPSQRRTGTPADDAGEVFLNLSLRVESQSEQAGRRNPPYAAVFHISDQTRRVIV
RYGEIERYLAEHPDTGIPGSRGLTSGLRVMSVDLGLRTSAAISVFRVAHRDELTPDAHGRQP
FFFPIHGMDHLVALHERSHLIRLPGETESKKVRSIREQRLDRLNRLRSQMASLRLLVRTGVL
DEQKRDRNWERLQSSMERGGERMPSDWWDLFQAQVRYLAQHRDASGEAWGRMVQAAVRTLWR
QLAKQVRDWRKEVRRNADKVKIRGIARDVPGGHSLAQLDYLERQYRFLRSWSAFSVQAGQVV
RAERDSRFAVALREHIDNGKKDRLKKLADRILMEALGYVYVTDGRRAGQWQAVYPPCQLVLL
EELSEYRFSNDRPPSENSQLMVWSHRGVLEELIHQAQVHDVLVGTIPAAFSSRFDARTGAPG
IRCRRVPSIPLKDAPSIPIWLSHYLKQTERDAAALRPGELIPTGDGEFLVTPAGRGASGVRV
VHADINAAHNLQRRLWENFDLSDIRVRCDRREGKDGTVVLIPRLTNQRVKERYSGVIFTSED
GVSFTVGDAKTRRRSSASQGEGDDLSDEEQELLAEADDARERSVVLFRDPSGFVNGGRWTAQ
RAFWGMVHNRIETLLAERFSVSGAAEKVRG

Cas12b coding sequence from *Bacillus sp.* NSP2.1 (GeneBank ID: NZ_KI301973.1)

ATGGCAATCCGTAGCATAAAACTAAAACTAAAAACCCACACAGGCCCGGAAGCGCAAAACCT
CCGAAAAGGAATATGGCGGACGCATCGGTTGTTAAATGAAGGCGTCGCCTATTACATGAAAA
TGCTCCTGCTCTTTCGTCAGGAAAGCACTGGTGAACGGCCAAAAGAAGAACTACAGGAAGAA
CTGATTTGTCACATACGCGAACAGCAACAACGAAATCAGGCAGATAAAAATACGCAAGCGCT
TCCGCTAGATAAGGCACTGGAAGCTTTGCGCCAACTATATGAACTGCTTGTCCCCTCCTCGG
TCGGACAAAGTGGCGACGCCCAGATCATCAGCCGAAAGTTTCTCAGCCCGCTCGTCGATCCG
AACAGCGAAGGCGGCAAAGGTACTTCGAAGGCAGGGGCAAAACCCACTTGGCAGAAGAAAAA
AGAAGCGAACGACCCAACCTGGGAACAGGATTACGAAAAATGGAAAAAAAGACGCGAGGAAG
ACCCAACCGCTTCTGTGATTACTACTTTGGAGGAATACGGCATTAGACCGATCTTTCCCCTG
TACACGAACACCGTAACAGATATCGCGTGGTTGCCACTTCAATCCAATCAGTTTGTGCGAAC
CTGGGACAGAGACATGCTTCAACAAGCGATTGAAAGACTGCTCAGTTGGGAGAGCTGGAACA
AACGTGTCCAGGAAGAGTATGCCAAGCTGAAAGAAAAAATGGCTCAACTGAACGAGCAACTC
GAAGGCGGTCAGGAATGGATCAGCTTGCTAGAGCAGTACGAAGAAAACCGAGAGCGAGAGCT
TAGGGAAAACATGACCGCTGCCAATGACAAGTATCGGATTACCAAGCGGCAAATGAAAGGCT
GGAACGAGCTGTACGAGCTATGGTCAACCTTTCCCGCCAGTGCCAGTCACGAGCAATACAAA
GAGGCGCTCAAGCGTGTGCAGCAGCGACTGAGAGGGCGGTTTGGGGATGCTCATTTCTTCCA
GTATCTGATGGAAGAGAAGAACCGCCTGATCTGGAAGGGGAATCCGCAGCGTATCCATTATT
TTGTCGCGCGCAACGAACTGACGAAACGGCTGGAGGAAGCCAAGCAAAGCGCCACGATGACG
TTGCCCAATGCCAGGAAGCATCCATTGTGGGTGCGCTTCGATGCACGGGGAGGAAATTTGCA

AGACTACTACTTGACGGCTGAAGCGGACAAACCGAGAAGCAGACGTTTTGTAACGTTTAGTC
AGTTGATATGGCCAAGCGAATCGGGATGGATGGAAAAGAAAGACGTCGAGGTCGAGCTAGCT
TTGTCCAGGCAGTTTTACCAGCAGGTGAAGTTGCTGAAAAATGACAAAGGCAAGCAGAAAAT
CGAGTTCAAGGATAAAGGTTCGGGCTCGACGTTTAACGGACACTTGGGGGGAGCAAAGCTAC
AACTGGAGCGGGGCGATTTGGAGAAGGAAGAAAAAAACTTCGAGGACGGGGAAATCGGCAGC
GTTTACCTTAACGTTGTCATTGATTTCGAACCTTTGCAAGAAGTGAAAAATGGCCGCGTGCA
GGCGCCGTATGGACAAGTACTGCAACTCATTCGTCGCCCCAACGAGTTTCCCAAGGTCACTA
CCTATAAGTCGGAGCAACTTGTTGAATGGATAAAAGCTTCGCCACAACACTCGGCTGGGGTG
GAGTCGCTGGCATCCGGTTTTCGTGTAATGAGCATAGACCTTGGGCTGCGCGCGGCTGCAGC
GACTTCTATTTTTTCTGTAGAAGAGAGTAGCGATAAAAATGCGGCTGATTTTTCCTACTGGA
TTGAAGGAACGCCGCTGGTCGCTGTCCATCAGCGGAGCTATATGCTCAGGTTGCCTGGTGAA
CAGGTAGAAAAACAGGTGATGGAAAAACGGGACGAGCGGTTCCAGCTACACCAACGTGTGAA
GTTTCAAATCAGAGTGCTCGCCCAAATCATGCGTATGGCAAATAAGCAGTATGGAGATCGCT
GGGATGAACTCGACAGCCTGAAACAAGCGGTTGAGCAGAAAAAGTCGCCGCTCGATCAAACA
GACCGGACATTTTGGGAGGGGATTGTCTGCGACTTAACAAAGGTTTTGCCTCGAAACGAAGC
GGACTGGGAACAAGCGGTAGTGCAAATACACCGAAAAGCAGAGGAATACGTCGGAAAAGCCG
TTCAGGCATGGCGCAAGCGCTTTGCTGCTGACGAGCGAAAAGGCATCGCAGGTCTGAGCATG
TGGAACATAGAAGAATTGGAGGGCTTGCGCAAGCTGTTGATTTCCTGGAGCCGCAGGACGAG
GAATCCGCAGGAGGTTAATCGCTTTGAGCGAGGCCATACCAGCCACCAGCGTCTGTTGACCC
ATATCCAAAACGTCAAAGAGGATCGCCTGAAGCAGTTAAGTCACGCCATTGTCATGACTGCC
TTGGGGTATGTTTACGACGAGCGGAAACAAGAGTGGTGCGCCGAATACCCGGCTTGCCAGGT
CATTCTGTTTGAAAATCTGAGCCAGTACCGTTCTAACCTGGATCGCTCGACCAAAGAAAACT
CCACCTTGATGAAGTGGGCGCATCGCAGCATTCCGAAATACGTCCACATGCAGGCGGAGCCA
TACGGGATTCAGATTGGCGATGTCCGGGCGGAATATTCCTCTCGTTTTTACGCCAAGACAGG
AACGCCAGGCATTCGTTGTAAAAAGGTGAGAGGCCAAGACCTGCAGGGCAGACGGTTTGAGA
ACTTGCAGAAGAGGTTAGTCAACGAGCAATTTTTGACGGAAGAACAAGTGAAACAGCTAAGG
CCCGGCGACATTGTCCCGGATGATAGCGGAGAACTGTTCATGACCTTGACAGACGGAAGCGG
AAGCAAGGAGGTCGTGTTTCTCCAGGCCGATATTAACGCGGCGCACAATCTGCAAAAACGTT
TTTGGCAGCGATACAATGAACTGTTCAAGGTTAGCTGCCGCGTCATCGTCCGAGACGAGGAA
GAGTATCTCGTTCCCAAGACAAAATCGGTGCAGGCAAAGCTGGGCAAAGGGCTTTTTGTGAA
AAAATCGGATACAGCCTGGAAAGATGTATATGTGTGGGACAGCCAGGCAAAGCTTAAAGGTA
AAACAACCTTTACAGAAGAGTCTGAGTCGCCCGAACAACTGGAAGACTTTCAGGAGATCATC
GAGGAAGCAGAAGAGGCGAAAGGAACATACCGTACACTGTTCCGCGATCCTAGCGGAGTCTT
TTTTCCCGAATCCGTATGGTATCCCCAAAAAGATTTTTGGGGCGAGGTGAAAAGGAAGCTGT
ACGGAAAATTGCGGGAACGGTTTTTGACAAAGGCTCGG

Humanized BsCas12b coding sequence

ATGGCCATCCGCAGCATCAAGCTGAAGCTGAAGACCCACACCGGCCCCGAGGCCCAGAACCT
GCGCAAGGGCATCTGGCGCACCCACCGCCTGCTGAACGAGGGCGTGGCCTACTACATGAAGA
TGCTGCTGCTGTTCCGCCAGGAGAGCACCGGCGAGCGCCCCAAGGAGGAGCTGCAGGAGGAG
CTGATCTGCCACATCCGCGAGCAGCAGCAGCGCAACCAGGCCGACAAGAACACCCAGGCCCT
GCCCCTGGACAAGGCCCTGGAGGCCCTGCGCCAGCTGTACGAGCTGCTGGTGCCCAGCAGCG
TGGGCCAGAGCGGCGACGCCCAGATCATCAGCCGCAAGTTCCTGAGCCCCCTGGTGGACCCC
AACAGCGAGGGCGGCAAGGGCACCAGCAAGGCCGGCGCCAAGCCCACCTGGCAGAAGAAGAA

GGAGGCCAACGACCCCACCTGGGAGCAGGACTACGAGAAGTGGAAGAAGCGCCGCGAGGAGG
ACCCCACCGCCAGCGTGATCACCACCCTGGAGGAGTACGGCATCCGCCCCATCTTCCCCCTG
TACACCAACACCGTGACCGACATCGCCTGGCTGCCCCTGCAGAGCAACCAGTTCGTGCGCAC
CTGGGACCGCGACATGCTGCAGCAGGCCATCGAGCGCCTGCTGAGCTGGGAGAGCTGGAACA
AGCGCGTGCAGGAGGAGTACGCCAAGCTGAAGGAGAAGATGGCCCAGCTGAACGAGCAGCTG
GAGGGCGGCCAGGAGTGGATCAGCCTGCTGGAGCAGTACGAGGAGAACCGCGAGCGCGAGCT
GCGCGAGAACATGACCGCCGCCAACGACAAGTACCGCATCACCAAGCGCCAGATGAAGGGCT
GGAACGAGCTGTACGAGCTGTGGAGCACCTTCCCCGCCAGCGCCAGCCACGAGCAGTACAAG
GAGGCCCTGAAGCGCGTGCAGCAGCGCCTGCGCGGCCGCTTCGGCGACGCCCACTTCTTCCA
GTACCTGATGGAGGAGAAGAACCGCCTGATCTGGAAGGGCAACCCCCAGCGCATCCACTACT
TCGTGGCCCGCAACGAGCTGACCAAGCGCCTGGAGGAGGCCAAGCAGAGCGCCACCATGACC
CTGCCCAACGCCCGCAAGCACCCCCTGTGGGTGCGCTTCGACGCCCGCGGCGGCAACCTGCA
GGACTACTACCTGACCGCCGAGGCCGACAAGCCCCGCAGCCGCCGCTTCGTGACCTTCAGCC
AGCTGATCTGGCCCAGCGAGAGCGGCTGGATGGAGAAGAAGGACGTGGAGGTGGAGCTGGCC
CTGAGCCGCCAGTTCTACCAGCAGGTGAAGCTGCTGAAGAACGACAAGGGCAAGCAGAAGAT
CGAGTTCAAGGACAAGGGCAGCGGCAGCACCTTCAACGGCCACCTGGGCGGCGCCAAGCTGC
AGCTGGAGCGCGGCGACCTGGAGAAGGAGGAGAAGAACTTCGAGGACGGCGAGATCGGCAGC
GTGTACCTGAACGTGGTGATCGACTTCGAGCCCCTGCAGGAGGTGAAGAACGGCCGCGTGCA
GGCCCCCTACGGCCAGGTGCTGCAGCTGATCCGCCGCCCCAACGAGTTCCCCAAGGTGACCA
CCTACAAGAGCGAGCAGCTGGTGGAGTGGATCAAGGCCAGCCCCCAGCACAGcgccggcgTG
GAGAGCCTGGCCAGCGGCTTCCGCGTGATGAGCATCGACCTGGGCCTGCGCGCCGCCGCCGC
CACCAGCATCTTCAGCGTGGAGGAGAGCAGCGACAAGAACGCCGCCGACTTCAGCTACTGGA
TCGAGGGCACCCCCCTGGTGGCCGTGCACCAGCGCAGCTACATGCTGCGCCTGCCCGGCGAG
CAGGTGGAGAAGCAGGTGATGGAGAAGCGCGACGAGCGCTTCCAGCTGCACCAGCGCGTGAA
GTTCCAGATCCGCGTGCTGGCCCAGATCATGCGCATGGCCAACAAGCAGTACGGCGACCGCT
GGGACGAGCTGGACAGCCTGAAGCAGGCCGTGGAGCAGAAGAAGAGCCCCCTGGACCAGACC
GACCGCACCTTCTGGGAGGGCATCGTGTGCGACCTGACCAAGGTGCTGCCCCGCAACGAGGC
CGACTGGGAGCAGGCCGTGGTGCAGATCCACCGCAAGGCCGAGGAGTACGTGGGCAAGGCCG
TGCAGGCCTGGCGCAAGCGCTTCGCCGCCGACGAGCGCAAGGGCATCGCCGGCCTGAGCATG
TGGAACATCGAGGAGCTGGAGGGCCTGCGCAAGCTGCTGATCAGCTGGAGCCGCCGCACCCG
CAACCCCCAGGAGGTGAACCGCTTCGAGCGCGGCCACACCAGCCACCAGCGCCTGCTGACCC
ACATCCAGAACGTGAAGGAGGACCGCCTGAAGCAGCTGAGCCACGCCATCGTGATGACCGCC
CTGGGCTACGTGTACGACGAGCGCAAGCAGGAGTGGTGCGCCGAGTACCCCGCCTGCCAGGT
GATCCTGTTCGAGAACCTGAGCCAGTACCGCAGCAACCTGGACCGCAGCACCAAGGAGAACA
GCACCCTGATGAAGTGGGCCCACCGCAGCATCCCCAAGTACGTGCACATGCAGGCCGAGCCC
TACGGCATCCAGATCGGCGACGTGCGCGCCGAGTACAGCAGCCGCTTCTACGCCAAGACCGG
CACCCCCGGCATCCGCTGCAAGAAGGTGCGCGGCCAGGACCTGCAGGGCCGCCGCTTCGAGA
ACCTGCAGAAGCGCCTGGTGAACGAGCAGTTCCTGACCGAGGAGCAGGTGAAGCAGCTGCGC
CCCGGCGACATCGTGCCCGACGACAGCGGCGAGCTGTTCATGACCCTGACCGACGGCAGCGG
CAGCAAGGAGGTGGTGTTCCTGCAGGCCGACATCAACGCCGCCCACAACCTGCAGAAGCGCT
TCTGGCAGCGCTACAACGAGCTGTTCAAGGTGAGCTGCCGCGTGATCGTGCGCGACGAGGAG
GAGTACCTGGTGCCCAAGACCAAGAGCGTGCAGGCCAAGCTGGGCAAGGGCCTGTTCGTGAA
GAAGAGCGACACCGCCTGGAAGGACGTGTACGTGTGGGACAGCCAGGCCAAGCTGAAGGGCA
AGACCACCTTCACCGAGGAGAGCGAGAGCCCCGAGCAGCTGGAGGACTTCCAGGAGATCATC

GAGGAGGCCGAGGAGGCCAAGGGCACCTACCGCACCCTGTTCCGCGACCCCAGCGGCGTGTT
CTTCCCCGAGAGCGTGTGGTACCCCCAGAAGGACTTCTGGGGCGAGGTGAAGCGCAAGCTGT
ACGGCAAGCTGCGCGAGCGCTTCCTGACCAAGGCCCGC

BsCas12b protein sequence

MAIRSIKLKLKTHTGPEAQNLRKGIWRTHRLLNEGVAYYMKMLLLFRQESTGERPKEELQEE
LICHIREQQQRNQADKNTQALPLDKALEALRQLYELLVPSSVGQSGDAQIISRKFLSPLVDP
NSEGGKGTSKAGAKPTWQKKKEANDPTWEQDYEKWKKRREEDPTASVITTLEEYGIRPIFPL
YTNTVTDIAWLPLQSNQFVRTWDRDMLQQAIERLLSWESWNKRVQEEYAKLKEKMAQLNEQL
EGGQEWISLLEQYEENRERELRENMTAANDKYRITKRQMKGWNELYELWSTFPASASHEQYK
EALKRVQQRLRGRFGDAHFFQYLMEEKNRLIWKGNPQRIHYFVARNELTKRLEEAKQSATMT
LPNARKHPLWVRFDARGGNLQDYYLTAEADKPRSRRFVTFSQLIWPSESGWMEKKDVEVELA
LSRQFYQQVKLLKNDKGKQKIEFKDKGSGSTFNGHLGGAKLQLERGDLEKEEKNFEDGEIGS
VYLNVVIDFEPLQEVKNGRVQAPYGQVLQLIRRPNEFPKVTTYKSEQLVEWIKASPQHSAGV
ESLASGFRVMSIDLGLRAAAATSIFSVEESSDKNAADFSYWIEGTPLVAVHQRSYMLRLPGE
QVEKQVMEKRDERFQLHQRVKFQIRVLAQIMRMANKQYGDRWDELDSLKQAVEQKKSPLDQT
DRTFWEGIVCDLTKVLPRNEADWEQAVVQIHRKAEEYVGKAVQAWRKRFAADERKGIAGLSM
WNIEELEGLRKLLISWSRRTRNPQEVNRFERGHTSHQRLLTHIQNVKEDRLKQLSHAIVMTA
LGYVYDERKQEWCAEYPACQVILFENLSQYRSNLDRSTKENSTLMKWAHRSIPKYVHMQAEP
YGIQIGDVRAEYSSRFYAKTGTPGIRCKKVRGQDLQGRRFENLQKRLVNEQFLTEEQVKQLR
PGDIVPDDSGELFMTLTDGSGSKEVVFLQADINAAHNLQKRFWQRYNELFKVSCRVIVRDEE
EYLVPKTKSVQAKLGKGLFVKKSDTAWKDVYVWDSQAKLKGKTTFTEESESPEQLEDFQEII
EEAEEAKGTYRTLFRDPSGVFFPESVWYPQKDFWGEVKRKLYGKLRERFLTKAR

pCAG-2AeGFP partial sequence

(CAG-NLS-*XmaI*-*NheI*-NLS-T2A-eGFP-SV40**)**

gacattgattattgactagttattaatagtaatcaattacggggtcattagttcatagccca
tatatggagttccGCGTTACATAACTTACGGTAAATGGCCCGCCTGGCTGACCGCCCAACGA
CCCCCGCCCATTGACGTCAATAATGACGTATGTTCCCATAGTAACGCCAATAGGGACTTTCC
ATTGACGTCAATGGGTGGACTATTTACGGTAAACTGCCCACTTGGCAGTACATCAAGTGTAT
CATATGCCAAGTACGCCCCCTATTGACGTCAATGACGGTAAATGGCCCGCCTGGCATTATGC
CCAGTACATGACCTTATGGGACTTTCCTACTTGGCAGTACATCTACGTATTAGTCATCGCTA
TTACCATGGGTCGAGGTGAGCCCCACGTTCTGCTTCACTCTCCCCATCTCCCCCCCCTCCCC
ACCCCCAATTTTGTATTTATTTATTTTTTAATTATTTTGTGCAGCGATGGGGGCGGGGGGGG
GGGGGGCGCGCGCCAGGCGGGGCGGGGCGGGGCGAGGGGCGGGGCGGGGCGAGGCGGAGAGG
TGCGGCGGCAGCCAATCAGAGCGGCGCGCTCCGAAAGTTTCCTTTTATGGCGAGGCGGCGGC
GGCGGCGGCCCTATAAAAAGCGAAGCGCGCGGCGGGCGGGAGTCGCTGCGTTGCCTTCGCCC
CGTGCCCCGCTCCGCGCCGCCTCGCGCCGCCCGCCCCGGCTCTGACTGACCGCGTTACTCCC
ACAGGTGAGCGGGCGGGACGGCCCTTCTCCTCCGGGCTGTAATTAGCGCTTGGTTTAATGAC
GGCTCGTTTCTTTTCTGTGGCTGCGTGAAAGCCTTAAAGGGCTCCGGGAGGGCCCTTTGTGC
GGGGGGGAGCGGCTCGGGGGGTGCGTGCGTGTGTGTGTGCGTGGGGAGCGCCGCGTGCGGCC
CGCGCTGCCCGGCGGCTGTGAGCGCTGCGGGCGCGGCGCGGGGCTTTGTGCGCTCCGCGTGT
GCGCGAGGGGAGCGCGGCCGGGGGCGGTGCCCCGCGGTGCGGGGGGGCTGCGAGGGGAACAA

AGGCTGCGTGCGGGGTGTGTGCGTGGGGGGTGAGCAGGGGGTGTGGGCGCGGCGGTCGGGC
TGTAACCCCCCCCTGCACCCCCCTCCCCGAGTTGCTGAGCACGGCCCGGCTTCGGGTGCGGG
GCTCCGTACGGGGCGTGGCGCGGGGCTCGCCGTGCCGGGCGGGGGGTGGCGGCAGGTGGGGG
TGCCGGGCGGGGCGGGGCCGCCTCGGGCCGGGGAGGGCTCGGGGGAGGGGCGCGGCGGCCCC
CGGAGCGCCGGCGGCTGTCGAGGCGCGGCGAGCCGCAGCCATTGCCTTTTATGGTAATCGTG
CGAGAGGGCGCAGGGACTTCCTTTGTCCCAAATCTGTGCGGAGCCGAAATCTGGGAGGCGCC
GCCGCACCCCCTCTAGCGGGCGCGGGGCGAAGCGGTGCGGCGCCGGCAGGAAGGAAATGGGC
GGGGAGGGCCTTCGTGCGTCGCCGCGCCGCCGTCCCCTTCTCCATCTCCAGCCTCGGGGCTG
TCCGCAGGGGGACGGCTGCCTTCGGGGGGGACGGGGCAGGGCGGGGTTCGGCTTCTGGCGTG
TGACCGGCGGCTCTAGcGCCTCTGCTAACCATGTTCATGCCTTCTTCTTTTTCCTACAGctc
ctgggcaacgtgctggttattgtgctgtctcatcattttggcaaaGCTAGTGAATTCTAATA
CGACTCACTATAGGCCGCCACCATGCCCAAGAAGAAGAGGAAGGTT*cccggggctagc*CCAA
AGAAGAAGAGGAAAGTCtctagaTACCCTTATGATGTTCCAGATTATGCCGGATACCCATAC
GATGTCCCTGACTATGCAGGCTCCTACCCTTATGACGTCCCAGACTACGCCggatccAGGTC
CGGCGGCGGAGAGGGCAGAGGAAGTCTTCTAACATGCGGTGACGTGGAGGAGAATCCCGGCC
CAATGGTGAGCAAGGGCGAGGAGCTGTTCACCGGGGTGGTGCCCATCCTGGTCGAGCTGGAC
GGCGACGTAAACGGCCACAAGTTCAGCGTGTCCGGCGAGGGCGAGGGCGATGCCACCTACGG
CAAGCTGACCCTGAAGTTCATCTGCACCACCGGCAAGCTGCCCGTGCCCTGGCCCACCCTCG
TGACCACCCTGACCTACGGCGTGCAGTGCTTCAGCCGCTACCCCGACCACATGAAGCAGCAC
GACTTCTTCAAGTCCGCCATGCCCGAAGGCTACGTCCAGGAGCGCACCATCTTCTTCAAGGA
CGACGGCAACTACAAGACCCGCGCCGAGGTGAAGTTCGAGGGCGACACCCTGGTGAACCGCA
TCGAGCTGAAGGGCATCGACTTCAAGGAGGACGGCAACATCCTGGGGCACAAGCTGGAGTAC
AACTACAACAGCCACAACGTCTATATCATGGCCGACAAGCAGAAGAACGGCATCAAGGTGAA
CTTCAAGATCCGCCACAACATCGAGGACGGCAGCGTGCAGCTCGCCGACCACTACCAGCAGA
ACACCCCCATCGGCGACGGCCCCGTGCTGCTGCCCGACAACCACTACCTGAGCACCCAGTCC
GCCCTGAGCAAAGACCCCAACGAGAAGCGCGATCACATGGTCCTGCTGGAGTTCGTGACCGC
CGCCGGGATCACTCTCGGCATGGACGAGCTGTACAAGtaactgcagcgcggggatctcatgc
tggagttcttcgcccaccccaacttgtttattgcagcttataatggttacaaataaagcaat
agcatcacaaatttcacaaataaagcatttttttcactgcattctagttgtggtttgtccaa
actcatcaatgtatctta

BPK2104-*ccdB* partial sequence

(*lac*I-T7-*lac*O-NLS-*XmaI*-*SpeI*-His$_{10}$-terminator)

TCACTGCCCGCTTTCCAGTCGGGAAACCTGTCGTGCCAGCTGCATTAATGAATCGGCCAACG
CGCGGGGAGAGGCGGTTTGCGTATTGGGCGCCAGGGTGGTTTTTCTTTTCACCAGTGAGACG
GGCAACAGCTGATTGCCCTTCACCGCCTGGCCCTGAGAGAGTTGCAGCAAGCGGTCCACGCT
GGTTTGCCCCAGCAGGCGAAAATCCTGTTTGATGGTGGTTAACGGCGGGATATAACATGAGC
TGTCTTCGGTATCGTCGTATCCCACTACCGAGATGTCCGCACCAACGCGCAGCCCGGACTCG
GTAATGGCGCGCATTGCGCCCAGCGCCATCTGATCGTTGGCAACCAGCATCGCAGTGGGAAC
GATGCCCTCATTCAGCATTTGCATGGTTTGTTGAAAACCGGACATGGCACTCCAGTCGCCTT
CCCGTTCCGCTATCGGCTGAATTTGATTGCGAGTGAGATATTTATGCCAGCCAGCCAGACGC
AGACGCGCCGAGACAGAACTTAATGGGCCCGCTAACAGCGCGATTTGCTGGTGACCCAATGC
GACCAGATGCTCCACGCCCAGTCGCGTACCGTCTTCATGGGAGAAAATAATACTGTTGATGG
GTGTCTGGTCAGAGACATCAAGAAATAACGCCGGAACATTAGTGCAGGCAGCTTCCACAGCA

ATGGCATCCTGGTCATCCAGCGGATAGTTAATGATCAGCCCACTGACGCGTTGCGCGAGAAG
ATTGTGCACCGCCGCTTTACAGGCTTCGACGCCGCTTCGTTCTACCATCGACACCACCACGC
TGGCACCCAGTTGATCGGCGCGAGATTTAATCGCCGCGACAATTTGCGACGGCGCGTGCAGG
GCCAGACTGGAGGTGGCAACGCCAATCAGCAACGACTGTTTGCCCGCCAGTTGTTGTGCCAC
GCGGTTGGGAATGTAATTCAGCTCCGCCATCGCCGCTTCCACTTTTTCCCGCGTTTTCGCAG
AAACGTGGCTGGCCTGGTTCACCACGCGGGAAACGGTCTGATAAGAGACACCGGCATACTCT
GCGACATCGTATAACGTTACTGGTTTCACATTCACCACCCTGAATTGACTCTCTTCCGGGCG
CTATCATGCCATACCGCGAAAGGTTTTGCGCCATTCGATGGTGTCCGGGATCTCGACGCTCT
CCCTTATGCGACTCCTGCATTAGGAAATTAATACGACTCACTATAGGGGAATTGTGAGCGGA
TAACAATTCCCCTGTAGAAATAATTTTGTTTAACTTTAATAAGGAGATATCATATGCCCAAG
AAGAAGAGGAAGGTT*cccgggctagt*CATCACCATCACCACCATCATCACCATCACTAGGC
GGCCGCATAATGCTTAAGTCGAACAGAAAGTAATCGTATTGTACACGGCCGCATAATCGAAA
TTAATacgactcactataggGAATTCGGTACCtgagaataactagcaTAACCCCTTGGGGCC
TCTAAACGGGTCTTGAGGGGTTTTTTGCTGAAACCTCAGGCATTT

pUC19-U6 partial sequence

(U6-*BasI*-*HindIII*)

TGTAAAACGACGGCCAGTGAATTCGAGGGCCTATTTCCCATGATTCCTTCATATTTGCATAT
ACGATACAAGGCTGTTAGAGAGATAATTGGAATTAATTTGACTGTAAACACAAAGATATTAG
TACAAAATACGTGACGTAGAAAGTAATAATTTCTTGGGTAGTTTGCAGTTTTAAAATTATGT
TTTAAAATGGACTATCATATGCTTACCGTAACTTGAAAGTATTTCGATTTCTTGGCTTTATA
TATCTTGTGGAAAGGACGAAACACCGGA*GAGACC*NNNNNNN*GGTCTC*ANNNNNNNNNNNNNN
NNNNNNNNNNNNNNNNNNNNNNNNNNNNNNNNNNNNNNNNNNNNNNNNNNNNNNNNNNNNNN
NNN*AAGCTT*GGCGTAATCATGGTCATAGCTGTTTCCTG

pUC19-U6-Aa_tracrRNA-tRNA-crRNA partial sequence

(U6-Aa_tracrRNA-tRNA-crRNA_scaffold-*BasI*-*BasI*-terminator)

TGTAAAACGACGGCCAGTGAATTCGAGGGCCTATTTCCCATGATTCCTTCATATTTGCATAT
ACGATACAAGGCTGTTAGAGAGATAATTGGAATTAATTTGACTGTAAACACAAAGATATTAG
TACAAAATACGTGACGTAGAAAGTAATAATTTCTTGGGTAGTTTGCAGTTTTAAAATTATGT
TTTAAAATGGACTATCATATGCTTACCGTAACTTGAAAGTATTTCGATTTCTTGGCTTTATA
TATCTTGTGGAAAGGACGAAACACCGGGTCTAAAGGACAGAATTTTTCAACGGGTGTGCCAA
TGGCCACTTTCCAGGTGGCAAAGCCCGTTGAACTTCTCAAAAAGAACGCTCGCTCAGTGTTC
TGACAACAAAGCACCAGTGGTCTAGTGGTGGAATAGTACCCTGCCACGGTACAGACCCGGGT
TCGATTCCCGGCTGGTGCAGTCGGATCACTGAGCGAGCGATCTGAGAAGTGGCACA*GAGACC*
GAGAGAG*GGTCTC*AtttttttAAGCTTGGCGTAATCATGGTCATAGCTGTTTCCTG

pUC19-U6-Ak_tracrRNA-tRNA-crRNA partial sequence

(U6-Ak_tracrRNA-tRNA-crRNA_scaffold-BasI-BasI-terminator)

TGTAAAACGACGGCCAGTGAATTCGAGGGCCTATTTCCCATGATTCCTTCATATTTGCATAT
ACGATACAAGGCTGTTAGAGAGATAATTGGAATTAATTTGACTGTAAACACAAAGATATTAG
TACAAAATACGTGACGTAGAAAGTAATAATTTCTTGGGTAGTTTGCAGTTTTAAAATTATGT
TTTAAAATGGACTATCATATGCTTACCGTAACTTGAAAGTATTTCGATTTCTTGGCTTTATA

TATCTTGTGGAAAGGACGAAACACCGGGTCGTCTATAGGACGGCGAGGACAACGGGAAGTGC
CAATGTGCTCTTTCCAAGAGCAAACACCCCGTTGGCTTCAAGATGACCGCTCGCTCAGCGAT
CTGACAACAAAGCACCAGTGGTCTAGTGGTGGAATAGTACCCTGCCACGGTACAGACCCGGG
TTCGATTCCCGGCTGGTGCAAACGGATCGCTGAGCGAGCGGTCTGAGAAGTGGCACAGAGAC
CGAGAGAGGGTCTCAttttttttAAGCTTGGCGTAATCATGGTCATAGCTGTTTCCTG

pUC19-U6-Am_tracrRNA-tRNA-crRNA partial sequence

(U6-Am_tracrRNA-tRNA-crRNA_scaffold-BasI-BasI-terminator)
TGTAAAACGACGGCCAGTGAATTCGAGGGCCTATTTCCCATGATTCCTTCATATTTGCATAT
ACGATACAAGGCTGTTAGAGAGATAATTGGAATTAATTTGACTGTAAACACAAAGATATTAG
TACAAAATACGTGACGTAGAAAGTAATAATTTCTTGGGTAGTTTGCAGTTTTAAAATTATGT
TTTAAAATGGACTATCATATGCTTACCGTAACTTGAAAGTATTTCGATTTCTTGGCTTTATA
TATCTTGTGGAAAGGACGAAACACCGGGGAATTGCCGATCTATAGGACGGCAGATTCAACGG
GATGTGCCAATGCACTCTTTCCAGGAGTGAACACCCCGTTGGCTTCAACATGATCGCCCGCT
CAACGGTCCGATAACAAAGCACCAGTGGTCTAGTGGTGGAATAGTACCCTGCCACGGTACAG
ACCCGGGTTCGATTCCCGGCTGGTGCAGTCGGATCACTGAGCGAGCGATCTGAGAAGTGGCA
CAGAGACCGAGAGAGGGTCTCAttttttttAAGCTTGGCGTAATCATGGTCATAGCTGTTTC
CTG

pUC19-U6-Bs_tracrRNA-tRNA-crRNA partial sequence

(U6-Bs_tracrRNA-tRNA-crRNA_scaffold-BasI-BasI-terminator)
TGTAAAACGACGGCCAGTGAATTCGAGGGCCTATTTCCCATGATTCCTTCATATTTGCATAT
ACGATACAAGGCTGTTAGAGAGATAATTGGAATTAATTTGACTGTAAACACAAAGATATTAG
TACAAAATACGTGACGTAGAAAGTAATAATTTCTTGGGTAGTTTGCAGTTTTAAAATTATGT
TTTAAAATGGACTATCATATGCTTACCGTAACTTGAAAGTATTTCGATTTCTTGGCTTTATA
TATCTTGTGGAAAGGACGAAACACCGGCCATAAGTCGACTTACATATCCGTGCGTGTGCATT
ATGGGCCCATCCACAGGTCTATTCCCACGGATAATCACGACTTTCCACTAAGCTTTCGAATA
ACAAAGCACCAGTGGTCTAGTGGTGGAATAGTACCCTGCCACGGTACAGACCCGGGTTCGAT
TCCCGGCTGGTGCAGTTCGAAAGCTTAGTGGAAAGCTTCGTGGTTAGCACAGAGACCGAGAG
AGGGTCTCAttttttttAAGCTTGGCGTAATCATGGTCATAGCTGTTTCCTG

pUC19-U6-AasgRNA partial sequence

(U6-AasgRNA_scaffold-*BasI*-*BasI*-terminator)
TGTAAAACGACGGCCAGTGAATTCGAGGGCCTATTTCCCATGATTCCTTCATATTTGCATAT
ACGATACAAGGCTGTTAGAGAGATAATTGGAATTAATTTGACTGTAAACACAAAGATATTAG
TACAAAATACGTGACGTAGAAAGTAATAATTTCTTGGGTAGTTTGCAGTTTTAAAATTATGT
TTTAAAATGGACTATCATATGCTTACCGTAACTTGAAAGTATTTCGATTTCTTGGCTTTATA
TATCTTGTGGAAAGGACGAAACACCGGGTCTAAAGGACAGAATTTTTCAACGGGTGTGCCAA
TGGCCACTTTCCAGGTGGCAAAGCCCGTTGAACTTCTCAAAAAGAACGCTCGCTCAGTGTTC
TGACGTCGGATCACTGAGCGAGCGATCTGAGAAGTGGCACA*GAGACC*GAGAGAG*GGTCTC*At
tttttttAAGCTTGGCGTAATCATGGTCATAGCTGTTTCCTG

pUC19-U6-AksgRNA partial sequence

(U6-AksgRNA_scaffold-*BasI*-*BasI*-terminator)

TGTAAAACGACGGCCAGTGAATTCGAGGGCCTATTTCCCATGATTCCTTCATATTTGCATAT
ACGATACAAGGCTGTTAGAGAGATAATTGGAATTAATTTGACTGTAAACACAAAGATATTAG
TACAAAATACGTGACGTAGAAAGTAATAATTTCTTGGGTAGTTTGCAGTTTTAAAATTATGT
TTTAAAATGGACTATCATATGCTTACCGTAACTTGAAAGTATTTCGATTTCTTGGCTTTATA
TATCTTGTGGAAAGGACGAAACACCGGtcgtctataGGACGGCGAGGACAACGGGAAGTGCC
AATGTGCTCTTTCCAAGAGCAAACACCCCGTTGGCTTCAAGATGACCGCTCGCTCAGCGATC
TGACAACGGATCGCTGAGCGAGCGGTCTGAGAAGTGGCACA*GAGACC*GAGAGAG*GGTCTC*At
tttttttAAGCTTGGCGTAATCATGGTCATAGCTGTTTCCTG

pUC19-U6-AmsgRNA partial sequence

(U6-AmsgRNA_scaffold-*BasI*-*BasI*-terminator)

TGTAAAACGACGGCCAGTGAATTCGAGGGCCTATTTCCCATGATTCCTTCATATTTGCATAT
ACGATACAAGGCTGTTAGAGAGATAATTGGAATTAATTTGACTGTAAACACAAAGATATTAG
TACAAAATACGTGACGTAGAAAGTAATAATTTCTTGGGTAGTTTGCAGTTTTAAAATTATGT
TTTAAAATGGACTATCATATGCTTACCGTAACTTGAAAGTATTTCGATTTCTTGGCTTTATA
TATCTTGTGGAAAGGACGAAACACCGGggaattgccgatctaTAGGACGGCAGATTCAACGG
GATGTGCCAATGCACTCTTTCCAGGAGTGAACACCCCGTTGGCTTCAACATGATCGCCCGCT
CAACGGTCCGATGTCGGATCGTTGAGCGGGCGATCTGAGAAGTGGCACA*GAGACC*GAGAGAG
*GGTCTC*AttttttttAAGCTTGGCGTAATCATGGTCATAGCTGTTTCCTG

pUC19-U6-BssgRNA partial sequence

(U6-BssgRNA_scaffold-*BasI*-*BasI*-terminator)

TGTAAAACGACGGCCAGTGAATTCGAGGGCCTATTTCCCATGATTCCTTCATATTTGCATAT
ACGATACAAGGCTGTTAGAGAGATAATTGGAATTAATTTGACTGTAAACACAAAGATATTAG
TACAAAATACGTGACGTAGAAAGTAATAATTTCTTGGGTAGTTTGCAGTTTTAAAATTATGT
TTTAAAATGGACTATCATATGCTTACCGTAACTTGAAAGTATTTCGATTTCTTGGCTTTATA
TATCTTGTGGAAAGGACGAAACACCGGCCATAAGTCGACTTACATATCCGTGCGTGTGCATT
ATGGGCCCATCCACAGGTCTATTCCCACGGATAATCACGACTTTCCACTAAGCTTTCGAATG
TTCGAAAGCTTAGTGGAAAGCTTCGTGGTTAGCACA*GAGACC*GAGAGAG*GGTCTC*Atttttt
ttAAGCTTGGCGTAATCATGGTCATAGCTGTTTCCTG
